# Stereochemical Behavior of Pyrrolo-Pyrazole Peptidomimetics Promoting Phase-Selective Supramolecular Organogels

**DOI:** 10.3390/gels10040263

**Published:** 2024-04-14

**Authors:** Enrica Chiesa, Francesco Anastasi, Francesca Clerici, Edoardo Mario Lumina, Ida Genta, Sara Pellegrino, Maria Luisa Gelmi

**Affiliations:** 1 Department of Drug Sciences, University of Pavia, Via Taramelli 12, 27100 Pavia, Italy; enrica.chiesa@unipv.it (E.C.);; 2 Department of Pharmaceutical Sciences (DISFARM), University of Milan, Via Venezian 21, 20133 Milano, Italy; francesco.anastasi@unimi.it (F.A.); edoardo.lumina@hotmail.com (E.M.L.)

**Keywords:** organogels, pyrrolo-pyrazole, peptidomimetic, phase-selective gelation, thermoreversibility

## Abstract

Supramolecular gels were developed by taking advantage of an assembly of small dipeptides containing pyrrolo-pyrazole scaffolds. The dipeptides were prepared through a robust and ecofriendly synthetic approach from the commercially available starting materials of diazoalkanes and maleimides. By playing with the functionalization of the scaffold, the choice of the natural amino acid, and the stereochemistry, we were able to obtain phase-selective gels. In particular, one peptidomimetic showed gelation ability and thermoreversibility in aromatic solvents at very low concentrations. Rheology tests showed a typical viscoelastic solid profile, indicating the formation of strong gels that were stable under high mechanical deformation. NMR studies were performed, allowing us to determine the conformational and stereochemical features at the base of the supramolecular interactions.

## 1. Introduction

The self-assembly of small peptides has received great attention in nanoscience and nanotechnology [1,2,3]. These molecules can be easily produced at a large scale and their spontaneous assembly can lead to the formation of ordered architectures, from nanotubes to spheres, micelles, and fibers. This hierarchical process is driven by complex, highly specific non-covalent interactions including hydrogen bonding, van der Waals and electrostatic forces, hydrophobic interactions, and the π-π stacking of aromatic rings [3]. Although several functional supramolecular structures have been reported in the last few decades, their design and preparation are still a key research interest. Furthermore, the possibility of introducing both natural and non-coded amino acids in the sequence could expand the scope of possible applications [4].

A particularly intriguing topic is the development of supramolecular gels, in which semi-solid materials are formed by entrapping large amounts of solvents in ordered 3D networks formed by the assembly of small molecules and peptides [5,6].

Due to the reversible nature of non-covalent interactions, the obtained supramolecular gels are highly sensitive and dynamic in nature [7,8]. When the entrapped solvent is an organic one, organogels are formed, and when 1% or less of the entrapping compound is needed for their formation, this compound is called a super-organogelator [9]. One profitable feature of an organogelator is the ability to induce the phase-selective gelation (PSG) of the organic phase from non-miscible solvents with water. The first report on an organic compound able to selectively gelate the oil phase from a two-phase mixture of water and oil was published by Bhattacharya and Ghosh in 2001 [10]. They exploited *N*-lauroyl alanine amphiphile, which selectively assembled into a fibrous network within the oil phase, leading to PSG. Afterwards, several other molecules with PSG properties were developed, finding various applications such dye absorption and oil-spill recovery [11]. In particular, PSGs showing gelation ability in aromatic solvents have been actively investigated, as they could find application in minimizing environmental pollution [12,13,14]. In this context, a challenging task is the development of PSGs enabled to work at room temperature, as the majority of them require a high-temperature trigger or the presence of an organic co-solvent to be efficient.

Only a few examples of the use of small peptides and peptidomimetics for PSG are reported in the literature. Their use is becoming of large interest due to their ability to generate well-defined secondary structures [15,16], as well as the possibility of introducing specific substituents to stabilize π,π interactions [17,18,19] or induce pH responsiveness [20]. All of these features allow the provision of molecules characterized by the structural diversity necessary to finely control the physical properties of products. On the other hand, the exploitation of peptidomimetics as supramolecular gelators could be hampered by their sometimes-difficult synthesis. As a result, there is a growing interest in obtaining gelator molecules through few synthetic steps, and in using non-expensive starting materials [21,22].

**Scheme 1:**
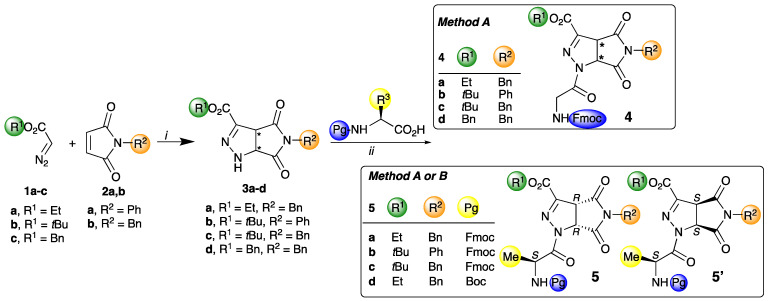
Synthesis of pyrrolo-pyrazole derivatives **3,** peptidomimetics **4,** and **5/5′** reagents and conditions. (*i*) Et_2_O, 25 °C, 12 h; (*ii*) *Method A*: Fmoc-GlyOH (1.2 mmol.; 0.05 M in CH_2_Cl_2_)/EDC (1.3 mmol)/HOBt (1.3 mmol)/1 h, 0 °C, then DIPEA (pH 7–8)/**3** (1 mmol) 25 °C, overnight; *Method B*: Fmoc-AlaOH (1.9 mmol; 0.01 M in CH_2_Cl_2_)/DIC (2.1 mmol)/OXIMA (2.1 mmol)/1 h, 0 °C, then **3** (1.5 mmol)/DIPEA (pH 7–8), 25 °C, overnight.

Continuing our research on non-coded amino acids able to generate specific secondary structures when inserted into peptides [23] and enhancing the spectrum of fundamental materials [24], we recently synthesized 3-carboxylic-pyrrolo-pyrazole derivatives **3** (Scheme 1), which can be considered as mimetics of γ-amino acid [25]. This versatile scaffold can be obtained in one step from commercially available compounds and can be easily decorated at the carboxylic function, at the-nitrogen of the imide function and of pyrazoline, affording a small library of compounds. Of relevance, their functionalization with Gly makes peptidomimetics able to form a homogeneous fibrous matrix by electrospinning.

Taking advantage of the above feature of pyrrolo-pyrazole scaffold **3**, here we aimed to expand the scope of its application by developing PSG containing this scaffold. Accordingly, a small library of peptidomimetics **4** (Scheme 1) characterized by a wide chemical variability was synthesized, allowing shifting from electrospun fibers to supramolecular gelators able to entrap aromatic solvents. 

Pyrrolo-pyrazole-containing gels were fully characterized by studying their molecular and supramolecular structures, morphology, and chemical–physical behavior. Rheological experiments were performed to assess the mechanical and thermal stability of organogels formulated with different organic solvents. NMR studies were performed to understand the conformational and stereochemical features at the base of supramolecular interactions. Transmission and scanning electron microscopy were used to emphasize the presence of certain conformations. 

Finally, the gelators’ efficiency at inducing the selective gelation of the organic phase into an aqueous/organic mixture was evaluated. Interestingly, compound **5b** (Scheme 1) underwent gelation at room temperature without changes in the water phase amount. This is an added value of our gelators, since one of the main limitations of some PSGs described in the literature is related to the need for heating and cooling cycles to induce the gelation, making this protocol troublesome for the removal of high flammability in most oil phases [17,26]. 

## 2. Results and Discussion

### 2.1. Synthesis 

Compounds **3a**–**d**, characterized by different ester functionalities (i.e., Et, *t*Bu, Bn) and *N*-phenyl or -benzyl groups in imide function, were prepared by exploiting the known 1,3-dipolar cycloaddition reaction from commercially available diazoalkanes **1a**–**c** (toluene solution) and compounds **2a,b**, operating in Et_2_O at 25 °C (12 h) [25,27]. As expected, single ∆^2^-pyrazoline regioisomers **3a**–**d** were obtained in good-to-excellent yields (Scheme 1). 

The coupling of compound **3** with Gly and Ala was then performed (Scheme 1). In particular, Fmoc-GlyOH was first activated with 1-ethyl-3-(3-dimethylaminopropyl)carbodiimide (EDC) and 1-hydroxybenzotriazole (HOBt), operating in dichloromethane (0.05 M, 1 h, 0 °C). Then, compound **3** was added together with diisopropylethylamine (DIPEA; pH 7–8). Pure peptides **4a**–**d** (50–73%) were isolated after purification on silica gel (*Method A*, Scheme 1). 

Scaffolds **3a**–**c** were then made to react with Fmoc- or Boc-Ala, affording a mixture of diastereoisomeric compounds **5** and **5′**. Operating in the above-described conditions (*Method A*) and starting from **3a** and **3d**, compounds **5a,d/5′a,d** were isolated in good yields (75 and 78%, respectively%). In addition, a 1,3-disopropylcarbodiimide (DIC)/ethyl cyanohydroxyiminoacetate (OXIMA)/DIPEA (pH 7–8) coupling system was used (*Method B*) in the case of the more hindered *t*Bu esters **3b,c** (1 equiv.), giving a mixture of the corresponding diastereoisomers **5b/b’** and **5c/c’** (85%). Diastereoisomers **5/5′** were successfully separated by silica gel chromatography. 

All compounds were characterized by NMR. Specifically, diastereoisomers **5/5′** (CDCl_3_, 400 MHz) showed significant differences: the ∆δ values of H*_3a_*/H*_6a_* were found to be ≥1 for **5** and <1 for the **5′** series. NH_Ala_ resonates to a higher field for **5** with respect to **5′**. A different trend was detected for the Me group of the Ala side chain. 

### 2.2. Gelation Studies 

The gelation phenomenon is the equilibrium state between dissolution and phase separation. In a gel system, the intermolecular forces are balanced. None of the compounds formed gels in water, while different results were obtained in organic solvents depending on the substitution patterns on the scaffold. The gelation results in different solvents at room temperature are reported in the Supporting Information: compound **4** (Table S1), diastereoisomers **5a,c,d/5′a,c,d** (Table S2), **5b** (Table S3), and **5′b** (Table S4). Compounds **4a**–**d**, all containing Gly, moiety failed in giving a gel (Table S1). On the other hand, diastereoisomers **5/5′** containing Ala afforded promising results. The most interesting compound was **5b,** containing the Fmoc group on Ala, the *t*Bu ester, and the *N-*phenyl as substituents on the pyrrolo-pyrazole core, whose gelation ability was deeply investigated (Table S3). Concerning aliphatic solvents, gels were formed only in mixtures of hexane/AcOEt (6:4) or *t*BuOMe (2% *w*/*v*), while **5b** in Et_2_O (1% *w*/*v*) provided a weak gel not suitable for any purpose. When the amount of **5b** increased, a partial dissolution was observed. On the other hand, all the tested aromatic solvents provided gels except for chloro-benzene, where **5b** was not fully soluble at the tested concentrations. The best results were achieved in toluene/dioxane (10:1), benzene, and *m*-xylene, using a substrate concentration of 2% *w*/*v* that led to a gel. 

Curiously, the diastereoisomer **5′b** was soluble in all the tested conditions except for hexane (Table S4), without giving a gel, indicating that the stereochemistry of the bicyclic ring plays a crucial role in gel formation. 

### 2.3. Rheology Experiments and Thermo-Reversibility 

**Figure 1:**
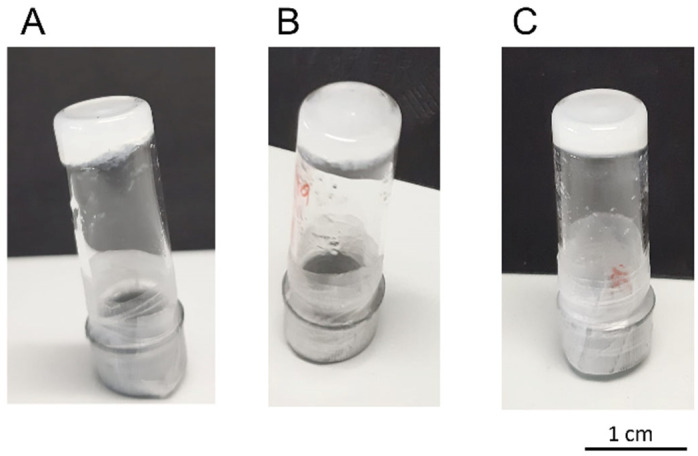
Visual inspections of **5b**-based organogels from (**A**) toluene/dioxane (10:1, 2% *w*/*v*), (**B**) *m-*xylene (2% *w*/*v*), and (**C**) benzene (2% *w*/*v*).

Rheology experiments were performed on **5b**-based supramolecular gels obtained from toluene/dioxane 10:1, xylene, and benzene (2% *w*/*v* of substrate for all solvents; Figure 1).

**Figure 2:**
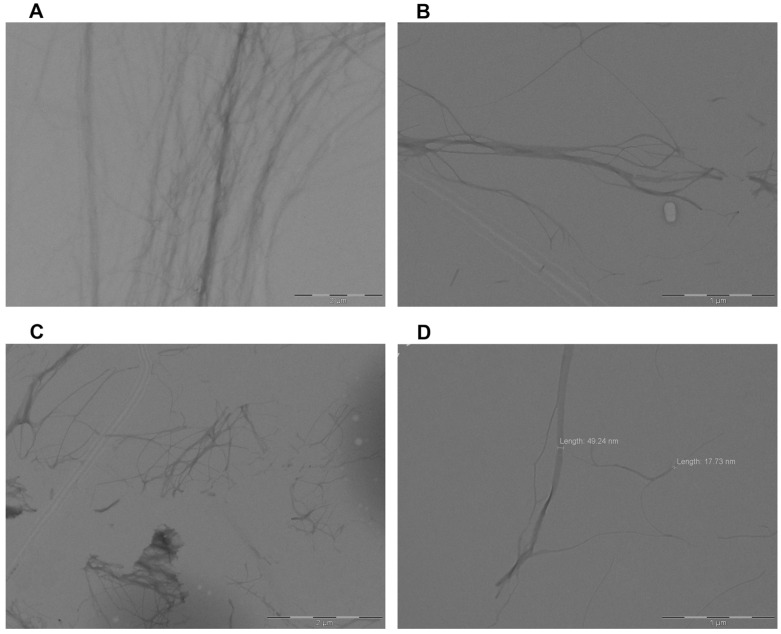
TEM images and fiber size distribution for **5b** self-assembly in (**A**) benzene, magnification 15,000× and (**B**) magnification 40,000×; and (**C**) toluene/dioxane, magnification 20,000× and (**D**) magnification 40,000×.

To elucidate the self-assembly mechanism of **5b** responsible for gelation, the organogel was observed by transmission electron microscopy (TEM). A two-fold dilution was carried out to facilitate the visualization. Figure 2 shows the formation of self-assembled interconnected fibrillar networks. The variable thickness of the fibrils was determined from 15 to 50 nm with variable length. Similar nanotubular bundles were observed for **5b** organogels in benzene (Figure 2A,B) and in toluene/dioxane (Figure 2C,D).

**Figure 3:**
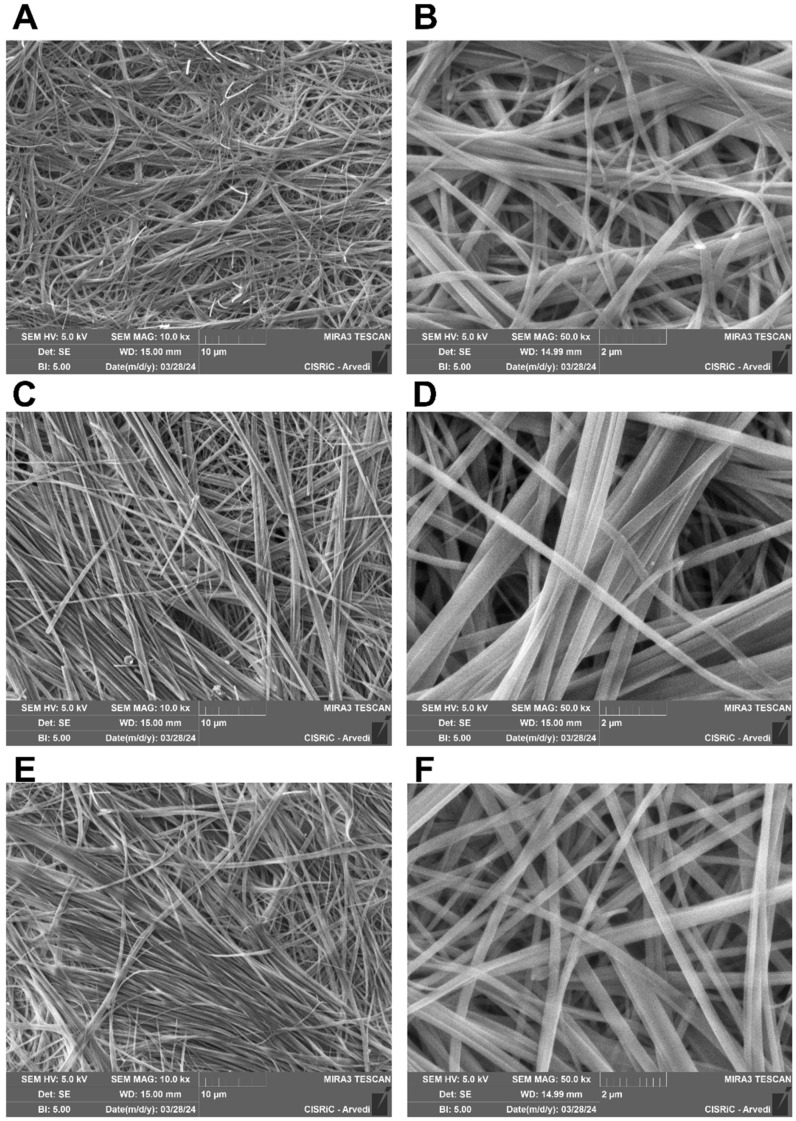
SEM images of **5b**-based organogels 2% *w*/*v* in benzene: (**A**) 10,000× magnification and (**B**) 50,000× magnification; toluene/dioxane (10:1): (**C**) 10,000× magnification and (**D**) 50,000× magnification; and *m*-xylene: (**E**) 10,000× magnification and (**F**) 50,000× magnification.

SEM images (Figure 3) of the dried organogels revealed a closely knit network of fibers, each exhibiting different thicknesses owing to the self-assembly ability of **5b**. Both TEM and SEM analyses underscored the gelation process of **5b**, demonstrating its ability, mediated by the pyrrole–pyrazole scaffold, to form fiber bundles with varying orientations facilitated by non-covalent interactions.

Amplitude sweeps are generally used to measure the linear viscoelastic region of a sample (LVER). In the LVER, the storage and loss moduli are independent of the applied deformation. The tested samples confirmed the gel-like behavior, since the storage modulus (G′) referring to the elastic properties of the material was always higher than the loss modulus (G″) correlated to the viscous characteristics. 

**Figure 4:**
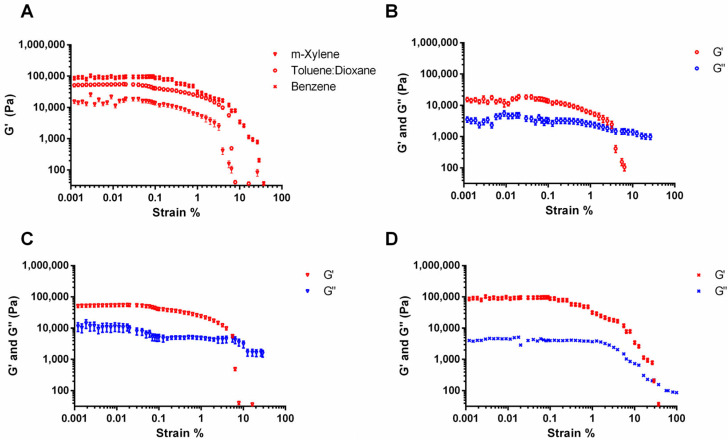
Amplitude sweep test: (**A**) storage modulus of **5b** in toluene/dioxane 10:1 (2% *w*/*v*; Δ), in *m*-xylene (2% *w*/*v*; ◯), and in benzene (2% *w*/*v*; x). Storage modulus (red symbols) and loss modulus (blue symbols) of **5b** organogels (**B**) in m-xylene, (**C**) in toluene/dioxane, and (**D**) in benzene.

The amplitude sweep for **5b** showed that the viscoelastic region was between 0.1 and 1 Pa stress and 0.001 and 0.5% strain (Figure 4). Strain/stress from within the linear region was used for all subsequent dynamic tests. Considering **5b**-based organogels, their mechanical strength was evaluated by comparing the values of G′ in the LVER. All the samples were gel since G′ differs from G″ by one order of magnitude. The results were influenced by the organic solvent used. Organogels with benzene (x) had higher G′ values compared to **5b** dissolved in xylene (◯) and in the mixture of toluene/dioxane (Δ), indicating a higher mechanical strength. Figure 4B–D show that with a gradual increase in strain, the values of both G′ and G″ are unchanged and get deviated from the linearity beyond a certain point (from 0.5 to 2% strain). The presence of a cross-point of G′ and G″ at high strain indicates that the organogel is broken. Close cross-points were observed for **5b**-based gels in xylene and toluene/dioxane, with 4–6% and 7–12% strains, respectively. Higher cross-points were observed for organogel in benzene (28–37% strains). A high cross-point is commonly observed for other supramolecular gels in the literature [28,29], which is attributed to the highly dynamic nature of these self-assembled systems. 

**Figure 5:**
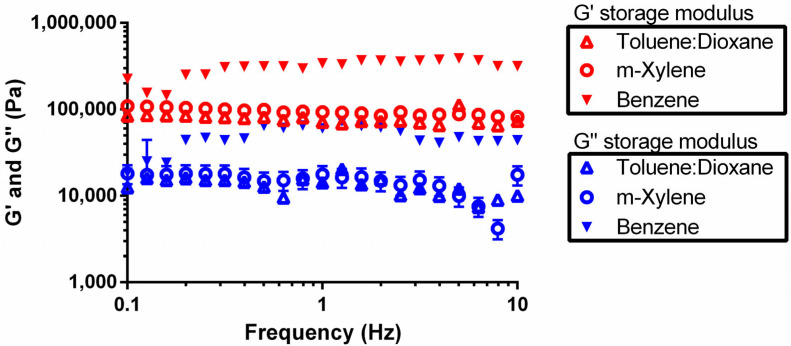
Frequency sweep test of **5b** in toluene/dioxane (10:1; Δ), *m-*xylene (◯), and benzene (▼). Red symbols correspond to storage modulus (G′), while blue symbols are loss modulus (G″).

The oscillation frequency sweep test involves oscillating the sample at fixed stress (0.5 Pa) while varying the frequency. Since this test is performed in the linear viscoelastic region, where structural integrity is not affected, a frequency sweep is considered the fingerprint spectrum of the material and is a very characteristic measurement of a sample. Depending on the type of material, three typical frequency responses can be revealed: visco-elastic solid, gel-like, and visco-elastic liquid [30]. As observed in Figure 5, the storage modulus for **5b** was always higher than the loss modulus for all the investigated solvents, denoting that the elastic character is always dominant when a load is applied. Considering all this evidence, **5b** shows a gel-like behavior. This type of sample can be classified as strong or weak depending on the relationship between the elastic modulus and the applied frequency [31]. A low dependence of elastic modulus on frequency was found for all the gels. This trend is commonly observed for strong gels that manifest the typical behavior of viscoelastic solids that rupture rather than flow above a critical deformation value [32]. This was also confirmed by the visual examination of the gels; at large deformations, **5b**-based organogels ruptured and failed without becoming liquid, and they were not able to recover. As observed before, **5b**-based organogels derived from benzene showed the highest strength with G′ values higher than 140 kPa, regardless of the frequency.

We then investigated the thermoreversibility of the gels obtained by **5b** (2% *w*/*v*; Table 1). Different melting temperatures and behaviors were observed depending on the solvent. Gel in benzene (entry 1) becomes a solution at 96 °C. Likewise, gels in *m*-xylene at 136 start to change to a viscous liquid (entry 2); neither are thermoreversible. Operating in toluene/dioxane (entry 3), a transition from gel to solution was observed at 60 °C. As the system cooled to room temperature, the solution became a gel within 10 min. Accordingly, compound **5b** is able to give a thermoreversible gel upon heating and returns to gel upon cooling.

**Figure 6:**
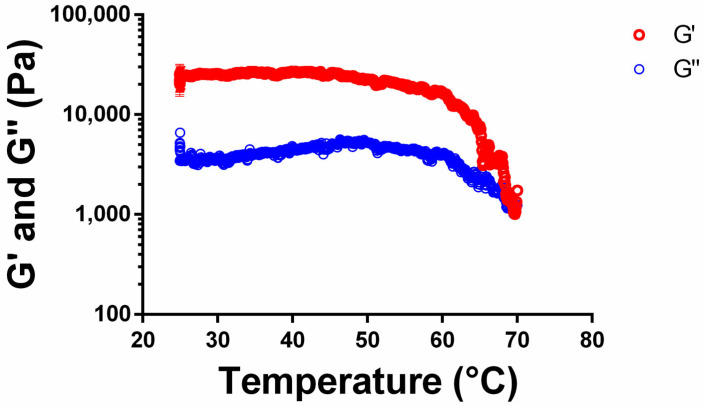
Temperature sweep test of **5b** organogel in toluene/dioxane (10:1; 2%*v*/*w*). Temperature was increased from RT to 70 °C at 2 °C/min.

Rheological changes in **5b** gels in toluene/dioxane (10:1; 2% *v*/*w*) across a temperature ramp were evaluated by a temperature sweep test (Figure 6). Temperature was increased from RT to 70 °C at a rate of 2 °C/min, and a constant frequency of 1 Hz and a strain percentage of 0.01% were used. As shown in Figure 5, the **5b** organogel in toluene/dioxane underwent gel–sol transition at 65–68 °C.

### 2.4. Phase-Selective Gelation (PSG)

An ideal phase-selective gelator (PSG) should exhibit organogelator skill at room temperature, water insolubility, and high mechanical rigidity in a gelled organic solvent. The gelation features of **5b** in various aromatic solvents motivated us to investigate whether it is a good candidate as a PSG for realistic applications in the separation and recovery of aromatic solvents. The PSG ability of compound **5b** was investigated by adding different percentages of compound **5b** to a mixture of organic solvent/water with (*Method A*) or without (*Method B*) shaking, and then keeping the mixture at 25 °C overnight (Table 2). At the organic-to-water volume ratio of 1:1, weak gel was formed at the interface of the water/organic layers. A residual solvent was observed and measured (Table 2, entries 1, 2, and 6) and the content of the organic phase in the gel was higher than 50% (*v*/*v*). The obtained organogel showed different morphological features compared to the organogel prepared in pure organic solvent. However, a complete PSG was achieved by adding 10 mg of **5b** in a biphasic mixture of benzene/water and *m*-xylene/water using a volume ratio of 1:5 (*v*/*v*) (Table 2, entries 5 and 8). No alteration in the water phase was observed. It can be speculated that the presence of water can interfere with gel formation and that a higher concentration of **5b** was needed. This mismatch has already been observed elsewhere in the literature, albeit with inconsistent findings [20].

**Figure 7:**
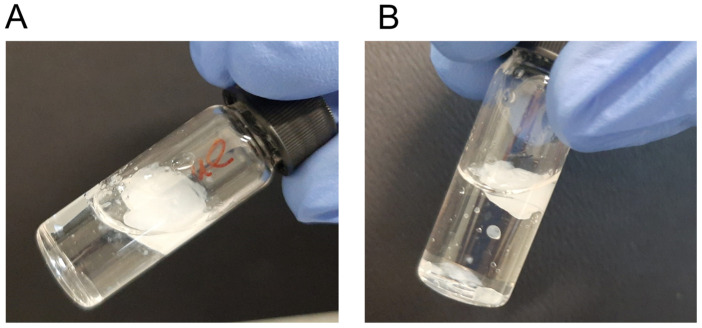
Visualization of phase-selective gelation of **5b** (10% *w*/*v*) in a mixture of benzene (400 µL) and water (2000 µL): (**A**) without shaking; (**B**) with shaking.

To visualize the PSG, **5b** (40 mg) was added to benzene (400 µL) and water (2000 µL). The gelation of the organic solvent compared to a biphasic system of water and organic solvents is shown in Figure 7. Interestingly, without shaking, **5b** underwent gelation at RT, forming a more compact organogel at RT without any residual organic solvent (Figure 7A).

### 2.5. NMR Studies

To gain more structural information on the aggregation process, **5b** was fully investigated with NMR experiments (^1^H, ^13^C, COSY, NOESY) in CDCl_3_ (1%), CDCl_3_/C_6_D_6_ (1:1; 1%), C_6_D_6_ (1%), and toluene-*_d8_*/dioxane-*_d8_* (10:1; 1%). The NMR technique is indeed very useful to understand the forces that influence self-assembly [11,33].

**Figure 8:**
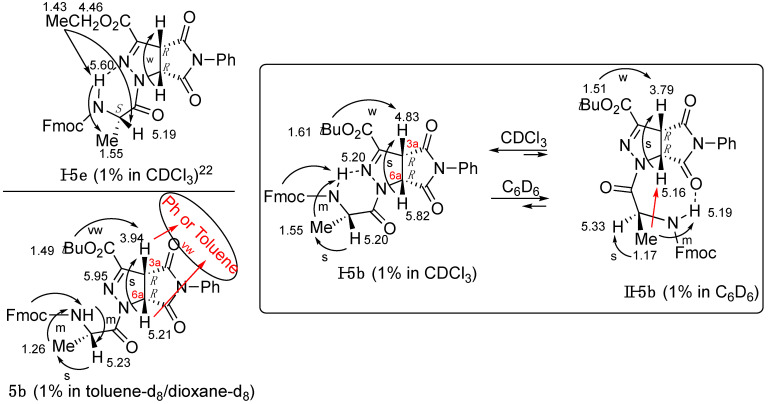
NOEs (arrow) and H-bonds (dash) of known **5e** and **5b**. Proposed conformers **I-5b** in CDCl_3_ and **II-5b** in C_6_D_6_. NOEs of **5b** in toluene-d_8_/dioxane-d_8_.

As shown in Table 3, a strong up-shift in all proton resonances was found by changing the solvent at 298 K from CDCl_3_ to a CDCl_3_/C_6_D_6_ mixture and C_6_D_6_ alone, except for CH_Ala_ and CH_2Fmoc_, which were down-shifted. Of relevance, it was observed that H-1 and H-8 of the symmetric fluorenyl scaffold were split in C_6_D_6_ (Figure S1), but not in CDCl_3_, indicating a different environment for these two protons. Furthermore, Ph-protons were also strongly up-shifted in benzene. These results suggest that a different conformation is adopted by **5b** in the two solvents. Indeed, two possible conformers can be present in solution due to the presence of a H-bond between NH and the nitrogen of C=N in the pyrazoline ring (six-member ring, conformer **I**, Figure 6) or the C=O of imide (δ-turn, conformer **II**, Figure 8). Interestingly, by changing the concentration of **5b** from 1% to 2% *w*/*v* in C_6_D_6_ (298 K), an up-shift of all protons was detected. This result, together with temperature-dependence experiments (see below), is of relevance when studying the driving force of the sol–gel process [34]. 

The NMR of **5b** in the toluene*_d8_*/dioxane*_d8_* (10:1; 1%) mixture showed resonances in between those observed in CDCl_3_ and C_6_D_6_, except for a strong down-shift in NH_Ala_ (Table 3). No significant changes were detected by increasing the concentration of **5b** (2%; Table 3).

Variable-temperature NMR experiments were then performed to investigate the potential NH involvement in H-bonds [35]. Low ∆δ/∆T values (1% CDCl_3_; 273–328 K; Figure S2) were detected for the NH_Ala_ of isomer **5b** (−2.1 ppb/K), suggesting the formation of an intramolecular H-bond. Focusing on the spectrum at 273 K, it should be pointed out that only a trace amount of a second conformer was detectable, indicating the formation of a stable conformer.

Considering the possibility of **5b** forming a gel in C_6_D_6_ at 2%, we focused on a systematic temperature-dependent NMR study, since it is well known that H-bonding is one of the major driving forces in organogelation for low-molecular-weight gelators [14]. In the first experiment, a clear solution of the fresh prepared sample of **5b** (2%) was recorded (Figure S3). A single conformer was detectable at all temperatures. No significant changes in chemical shifts were detected at the different temperatures. Clear broad signals were those of aromatic protons at 298 K that became sharp after heating. A similar behavior was observed for aliphatic protons, except for CH_Ala_, which was broad at all temperatures. The very low ∆δ/∆T value for NH_Ala_ (−0.67 ppb/K) has to be pointed out, indicating the formation of a strong intramolecular H-bond. 

In the second experiment, the sample was prepared the day before to ensure the formation of a gel. The spectra of **5b** in C_6_D_6_ were recorded from 298 to 348 K (Figure S4, Table S5), where the dissolution of the gel was observed. Then, the sample was cooled from 348 to 283 K, where the gel was obtained again (Figure S5, Table S5). Finally, the ^1^H NMR of this last sample was recorded after 24 h at 298 K (Figure S6).

Compared to the freshly prepared sample, in which the chemical shift of H_Ala_, NH_Ala_, and H_6a_ were well defined (δ 5.24–5.26 region; Figure S3), a different behavior was detected when starting from the gel (Figures S4 and S5). In this last case, NH and H_6a_ signals were very close, resonating in the δ 5.16–5.06 region. The H_Ala_ signal was isolated at 298 K but overlapped with the other signals when heating. Even if it is impossible to exactly define the NH resonance at 348 K, overall, the ∆δ/∆T value cannot be more than 1.4 ppb/K, indicating the formation of a strong intramolecular H-bond. The presence of intermolecular H-bonds is excluded since the example in the literature showed values higher than 5 ppb/K when intermolecular H-bonds were at the base of the formation of the gel [11,14]. 

As for the signal shape, a loss of resolution was found when lowering the temperature, as shown in Figures S5 and S6, but mostly in Figure S7. Focusing on the aromatic region when heating, not only was a shift in aromatic protons detected (Table S5), but so was a strong change in their resolution, mostly for the Fmoc group. All the above data indicate the transition of the gel–sol process, confirming the trend reported in the literature for gels [14,33,34].

Focusing on the experiment in toluene*_d8_*/dioxane*_d8_* at variable temperatures (from 298 to 363 K; Figure S7) of a sample of **5b** recorded 24 h after its preparation, as expected, a change in the state of the sample and an increase in the resolution of the signals, as well as their resonances, were detected. A ∆δ/∆T value of −7.5 ppb/K was detected, excluding the formation of an intramolecular H-bond. On the other hand, the formation of an intermolecular H-bond cannot be excluded.

In a second step, NOESY analyses were performed, helping us to obtain information on the conformation of compound **5b** according to the intramolecular H-bond formation detected with the experiment at variable temperatures in different solvents, and to define the absolute stereochemistry at C_3a_ and C_6a_ (Figures S8–S12). The complete set of chemical shifts and NOEs for **5b** in the different solvents is reported in Figure 6 and Figure S1. Operating in CDCl_3_ (1%, 293 K; Figure S8), the following spatial proximities were found: (i) Me_Ala_ with both NHAla and HAla, and (ii) H_3a_ with both *t*Bu and H_6a_, and those in between the protons of the Fmoc group. NH_Ala_ showed a weak NOE with aromatic H_Fmoc_ (δ 7.62). In a recent work, recording the spectrum of compound **5e** (CDCl_3_; [25] Figure 5), an analogous form of **5b**, we found that NH_Ala_ showed spatial proximity with the ethyl ester (not detected for **5b,** in which a *t*Bu*-*ester is present). Accordingly, we propose the formation of a six-member ring due to a H-bond of NH with C=N in the triazoline ring (conformer **I**, Figure 5). The involvement of C=N in the isooxazoline ring as a H-bond acceptor has already been observed [34]. Similar NOEs were found for compound **5b** in 1% CDCl_3_/C_6_D_6_ (1:1; Figure S9). Performing the NOESY experiment in C_6_D_6_ (1%; Figure S10A), similar results were achieved as in CDCl_3_, except for the spatial proximity between Me_Ala_ and H_6a_ (Figure S10B). This last result is consistent with the formation of a δ-turn stabilized by a H-bond between NH and the carbonyl of imide (conformer **II**, Figure 6). Of relevance, this NOE allowed us to define the absolute *R,R-*stereochemistry for diastereomer **5b**, first eluted in column chromatography (EtOAc/hexane).

The NOESY experiment in toluene/dioxane gave similar results as in CDCl_3_ (Figure S10A), except for the medium spatial proximity of both H_6a_ and H_3a_ with aromatic protons in the 7.07 region (Figure S10B). Since there is no intramolecular spatial proximity between H_6a_ and H_3a_ and the Ph ring, resonating in the above region, it must be assumed that intermolecular NOEs occurred with toluene or the Ph-group of a second molecule of **5b**. These spatial proximities were not observed in CDCl_3_ and C_6_D_6_, confirming again the different behavior of **5b** in the two solvents.

Focusing on *S,S-***5′b**, similar results to **5b** were observed: the change in the solvent from CDCl_3_ to C_6_D_6_ strongly affects the chemical shifts, as reported in Table S6. Furthermore, differently from **5b** in the same solvent, two main conformers were detected in C_6_D_6_. 

The experiment at variable temperature in CDCl_3_ (1%, 273–328 K; Figure S13) showed that ∆δ/∆T = −3.2 ppb/K, a slightly higher value with respect to *R,R*-**5b**. The same experiment in C_6_D_6_ (2%, 273–323 K; Figure S14) gave a lower ∆δ/∆T value (−2.8 ppb/K), but higher than **5b** (−0.67 ppb/K) in the same solvent.

The NOESY experiment of *S,S-***5′b** in CDCl_3_ (293 K; Figure S15) showed similar results to those observed for **5b,** but with stronger spatial proximities between the fluorenyl group and both CH_Ala_ and NH_Ala_ (Figure S15A). Furthermore, a very weak NOE between the *t*Bu group and CH_Ala_ was detected (Figure S15B), confirming the formation of the NH-bond with C=N, i.e., conformer **I** in Figure S12, as well as the behavior of these compounds in CDCl_3_ in forming the six-pseudo ring by H-bond stabilization.

A more complex NOE set was found in C_6_D_6_ (293 K; Figure S16) due to the presence of a couple of conformers, also at 293 K, and several overlapped signals. Similar spatial proximities observed for **II-5b** were detected for the main conformer **II-5′b**. As expected, no spatial proximity was found between Me_Ala_ and H_6a_. Due to the similar chemical shift of H_Ala/_H_6a_, we cannot define possible spatial proximities. The second isomer is probably consistent with **I-5′b**. Experiments at 283 K were also performed, giving a better definition of the two conformers (see COSY, Figure S17). Unfortunately, the NOESY experiment showed an equilibrium between the two conformers, preventing information on the spatial proximity between the aliphatic protons (Figure S18).

In summary, the conformation of peptidomimetics **5** and **5′** is strongly affected by the solvent, with the stability of conformer **I** confirmed for both isomers *R,R*-**5b** and *S,S*-**5′b** in CD_3_Cl. On the other hand, conformer **II** is stable in C_6_D_6_ for *R,R*-**5b**, but an equilibrium between **I** and **II** for *S,S*-**5′b** was observed in the same solvent. 

## 3. Conclusions

A small library of peptidomimetics **4** and **5/5′,** containing a pyrrolo-pyrazole scaffold and, respectively, Gly and Ala natural amino acids, was prepared in two steps from easily available compounds. Compound **5b** was found to be a low-molecular-weight organogelator, giving good performances mostly in aromatic solvents, and being a thermoreversible gel in toluene/dioxane. It was also able to induce phase-selective gelation. The gelation process reflects the self-assembly ability of the peptidomimetics into fibers, forming a network where the organic solvent is entrapped.

The rheological properties of **5b** in different solvents were analyzed. An amplitude sweep test was carried out to determine the LVER of the organogels, as well as to evaluate the gels’ strengths. Frequency sweep tests confirmed that the strength of the organogels was affected by the organic solvent. 

Moreover, **5b** organogels showed a typical viscoelastic solid profile and they were found to be strong gels since, upon high mechanical deformation, they collapsed without becoming a liquid. Based on the organic solvent used for the gelation, a different stress point was revealed: gel in benzene breaks at the highest strain percentage (28–37% strain), indicating a highly dynamic self-assembly. Gels made in toluene/dioxane lose their structure at 7–12% strain, showing a gel–sol transition at 65–70 °C, as confirmed by rheological measurements. 

NMR studies supported the mechanism of gel formation in aromatic solvents through supramolecular interactions, as the resonances shift by changing the concentration of the sample, as well as broadening and decreasing the intensity of aromatic peaks at low temperature. Several considerations can be made on the effect of the functionalization pattern of the scaffold toward gel formation. First, it was revealed that the presence of Ala, as well as the *R,R* stereochemistry of the pyrrolo-pyrazole scaffold in combination with aromatic solvents, are important for gel formation. The contribution of the aromatic groups, giving π-π interactions, is fundamental to trigger self-assembly. It was indeed found that the Ph group is better than the benzyl one, probably due to its coplanarity with the pyrrolidindione system, as well as the Fmoc group, with respect to Boc. Furthermore, bulky lipophilic alkyl substituents (*t*Bu vs. Et and Me_Ala_ vs. H_Gly_) were favorable in the formation of the gels. 

The aromatic solvent plays a key role in the formation of the gels: a stable δ-turn structure, due to the formation of a very strong intramolecular H-bond in benzene affording conformer **II** (Figure 6), is formed. This formation allows the orientation of the lipophilic groups (alkyl group of the ester and Me_Ala_) involved in the Van der Waals interactions on the same side and the aromatic ones (Fmoc and imide substituent) on the opposite side. On the other hand, no intramolecular H-bonds were detected for **5b** in toluene/dioxane, but intermolecular interactions of the bicyclic ring with aromatic moieties were detected. 

Finally, **5b** was demonstrated as an organogelator, and it could be of potential interest in several applications, such as environmental ones [36,37].

## 4. Materials and Methods

### 4.1. General

NMR spectroscopic experiments were carried out, either on Varian MERCURY 300 MHz (300 and 75 MHz for ^1^H and ^13^C, respectively) or Bruker Avance I 400 MHz spectrometers (400 and 101 MHz for ^1^H and ^13^C, respectively). Chemical shifts (δ) are given in ppm relative to the CHCl_3_ internal standard, and the coupling constants *J* are reported in Hertz (Hz). Mass spectra were recorded on an LCQESI MS, on a LCQ Advantage spectrometer from Thermo Finningan, and a LCQ Fleet spectrometer from Thermo Scientific. Optical rotations were measured on a PerkinElmer 343 polarimeter (concentration in g/100 mL). Diazoalkanes **1a**–**c**, maleimides **2a,b,** and protected amino acids are commercially available compounds. 

### 4.2. General Procedure for the Cycloaddition Reaction: Synthesis of Compound **3**

Maleimide **2** (1 equiv.) was dissolved in Et_2_O (50 mL) and diazoacetate **1** (**1a**: 1.1 equiv., 13% in CH_2_Cl_2_; **1b:** 1.1 equiv., 15% in toluene; **1c:** 1.1 equiv., 10% in toluene) was added. The reaction mixture was stirred at room temperature overnight and monitored by TLC (AcOEt/hexane, 4:6). A solid was formed that was filtered and washed with a few drops of Et_2_O. Cycloadducts **3a**,**b**,**d** were recently prepared by our group [25].

*ter-Butyl 4,6-dioxo-5-benzyl-1,3a,4,5,6,6a-hexahydropyrrolo [3,4-c]pyrazole-3-carboxylate (**3c**):* white solid. Yields: 93%. Mp 143.8 °C dec. (Et_2_O); ^1^H NMR (300 MHz, CDCl_3_): δ 7.37–7.26 (m, 5H), 6.80 (s, 1H), 4.83, 4.49 (AX system, *J* = 10.7 Hz, 2H), 4.64 (s, 2H), 1.56 (s, 9H); ^13^C NMR (75 MHz, CDCl_3_) δ 173.8, 170.9, 159.5, 138.6, 134.8, 128.7, 128.2, 82.9, 63.5, 51.7, 42.9, 27.9; MS (ESI+): *m*/*z* calcd for [C_17_H_19_N_3_NaO_4_]: 352.35; found: *m*/*z* 352.83.

### 4.3. General Procedure for Amino Acid Functionalization of Scaffold 3: Synthesis of Compounds **4** and 5/5′

*Method A:* Fmoc-Glycine-OH or Fmoc-Alanine-OH (1.2 mmol) was dissolved in CH_2_Cl_2_ (0.05 M solution) at 0 °C under magnetic stirring. EDC (202 mg, 1.3 mmol) and HOBt (176 mg, 1.3 mmol) were added and the solution was stirred for 1 h at 0 °C. Then, compound **3** (1 mmol) was added and the pH was corrected to 7–8 with DIPEA (364 μL). The reaction, monitored by TLC, was stirred at 25 °C overnight. The mixture was washed with a solution of KHSO_4_ (5% *w*/*v*), then with a saturated solution of NaHCO_3_, and finally with a saturated solution of NaCl. The organic layer was dried over anhydrous Na_2_SO_4_, filtered, and evaporated under reduced pressure. Pure products **4a**–**d** were obtained by Biotage Isolera One Flash Chromatography System purification, affording white solids. Diastereoisomers **5a/5′a** or **5d/5′d** were efficiently separated using the same system. *Method B:* Fmoc-Alanine-OH (614 mg, 1.9 mmol) was dissolved in CH_2_Cl_2_ (0.01 M solution) at 0 °C under magnetic stirring. DIC (333 μL, 2.1 mmol) and OXIMA (302 mg, 2.1 mmol) were added and the solution was stirred for 1 h at 0 °C. Then, compound **3** (1.5 mmol) was added and the pH was corrected to 7–8 with DIPEA (530 μL). The reaction, monitored by TLC, was stirred at 25 °C overnight. The solution was washed with a solution of KHSO_4_ (5% *w*/*v*), then with a saturated solution of NaHCO_3_ and a saturated solution of NaCl. The organic layer was dried over anhydrous Na_2_SO_4_, filtered, and evaporated under reduced pressure. Pure diastereoisomers **5b/5′b** and **5c/5′c** were separated with the Biotage Isolera One Flash Chromatography System, affording white solids.

Yields and spectroscopic analyses for **4a,b,d** are in agreement with the data recently reported by our group [25].

tButyl 3aR*,6aR*-1-(Fmoc-glycyl)-4,6-dioxo-5-benzyl-1,3a,4,5,6,6a-hexahydropyrrolo [3,4-c]pyrazole-3-carboxylate (**4c**): Method A; column chromatography: hexane, 100% to hexane/AcOEt, 1:1). Yield: 73%. Mp 164.2 °C, dec.; ^1^H NMR (300 MHz, DMSO-d_6_) δ 7.88 (d, *J* = 7.8 Hz, 2H), 7.75–7.65 (m, 2H), 7.44–7.14 (m, 9H), 5.55, 4.69 (AX system, *J* = 10.1 Hz, 2H), 4.51 (s, 2H), 4.33–4.18 (m, 4H), 4.07–4.01 (m, 2H), 1.48 (s, 9H); ^13^C NMR (75 MHz, CDCl_3_): δ 172.6, 170.7, 166.2, 159.0, 157.0, 144.9, 144.3, 141.2, 135.9, 128.9, 128.1, 128.0, 127.6, 125.7, 120.6, 83.6, 66.3, 62.5, 54.0, 47.0, 43.1, 28.0; MS (ESI+): *m*/*z* calcd for [C_34_H_32_N_4_NaO_7_]: 631.64; found: *m*/*z* 631.77

Ethyl 3aR*,6aR*-1-(Fmoc-alanyl)-4,6-dioxo-5-benzyl-1,3a,4,5,6,6a-hexahydropyrrolo [3,4-c]pyrazole-3-carboxylate (**5a/5′a**): Method A; column chromatography: hexane 100% to hexane/AcOEt, 1:1.

**3a*R*,6a*R-*5a:** 41%. Mp 165.1 °C, dec.; [α]_D_^20^ −1.6 (*c* 1.00, CHCl_3_). ^1^H NMR (400 MHz, CDCl_3_) δ 7.78 (d, *J* = 7.3 Hz, 2H), 7.65–7.59 (m, 2H), 7.45–7.30 (m, 9H), 5.64, 4.69 (AX system, *J* = 10.4 Hz, 2H), 5.52 (d, *J* = 8.2 Hz, 1H), 5.20–5.10 (m, 1H), 4.67 (s, 2H), 4.49–4.32 (m, 4H), 4.27–4.20 (m, 1H), 1.49 (d, *J* = 7.7, 3H), 1.42 (t, *J* = 6.7 Hz, 3H); ^13^C NMR (100.7 MHz, CDCl_3_) δ 172.5, 169.9, 168.8, 159.5, 155.7, 143.9, 143.7, 143.1, 141.32, 141.29, 134.5, 129.1, 128.9, 128.5, 127.7, 127.1, 125.2, 125.1, 120.0, 67.2, 62.9, 61.0, 52.5, 48.8, 47.1, 43.5, 18.7, 14.1; MS (ESI): *m*/*z* calcd for [C_33_H_31_N_4_O_7_] 595.22; found: *m*/*z* 595.32, 617.76 [M+Na].

**3a*S*,6a*S-*5′a:** 37%. Mp 148.8 °C, dec.; [α]_D_^20^ +1.5 (*c* 1.00, CHCl_3_). ^1^H NMR (400 MHz, CDCl_3_) δ 7.77 (d, *J* = 7.2 Hz, 2H), 7.65–7.55 (m, 2H), 7.45–7.26 (m, 9H), 5.66 (d, *J* = 7.7 Hz, 1H), 5.55, 4.71 (AX system, *J* = 10.4 Hz, 2H), 5.23–5.09 (m, 1H), 4.65 (s, 2H), 4.47–4.31 (m, 4H), 4.26–4.17 (m, 1H), 1.46–1.31 (m, 6H); ^13^C NMR (100.7 MHz, CDCl_3_) δ 172.0, 169.2, 168.6, 159.5, 155.3, 143.9, 143.8, 141.3, 134.3, 129.3, 128.9, 128.5, 127.7, 127.1, 125.2, 120.0, 67.1, 62.9, 61.0, 52.6, 49.1, 47.1, 43.7, 19.0, 14.1; MS (ESI): *m*/*z* calcd for [C_33_H_34_N_4_O_7_] 594.62; found: *m*/*z* 595.63.

tButyl 3aR*,6aR*-1-(Fmoc-alanyl)-4,6-dioxo-5-phenyl-1,3a,4,5,6,6a-hexahydropyrrolo[3,4-c] pyrazole-3-carboxylate (**5b/5′b**): Method B; column chromatography: hexane 100% to hexane/AcOEt, 6:4.

**3a*R*,6a*R-*5b**: 41%. Mp 129.6 °C, dec.; [α]_D_^20^ −2.0 (*c* 1.00, CH_2_Cl_2_). ^1^H NMR (400 MHz, CDCl_3_) δ 7.78 (d, *J* = 7.8 Hz, 2H), 7.66–7.58 (m, 2H), 7.53–7.26 (m, 9H), 5.82, 4.83 (AX system, *J* = 10.5 Hz, 2H), 5.54 (d, *J* = 8.3 Hz, 1H), 5.25–5.13 (m, 1H), 4.46–4.29 (m, 2H), 4.29–4.21 (m, 1H), 1.61 (s, 9H), 1.55 (d, *J* = 7.2, 3H); ^13^C NMR (100.7 MHz, CDCl_3_) δ 172.5, 169.5, 168.3, 158.2, 155.8, 144.8, 143.8, 143.7, 141.3, 141.2, 130.8, 129.3, 129.2, 127.7, 127.1, 126.1, 125.2, 125.1, 119.9, 84.9, 67.1, 60.9, 52.7, 48.9, 47.1, 27.9, 18.6. ^1^H NMR (400 MHz, 1% in C_6_D_6_) δ 7.70 (d, *J* = 7.2 Hz, 2H), 7.65 (d, *J* = 7.2 Hz, 1H), 7.57 (d, *J* = 7.2 Hz, 1H), 7.42–7.28 (m, 4H), 7.15–6.97 (m, 5H), 5.40–5.26 (m, 1H), 5.18 (d, *J* = 7.7 Hz, 1H), 5.16, 3.79 (AX system, *J* = 10.3 Hz, 2H), 4.57, 4.46, 4,22 (ABX system, *J =* 16.4, 7.1, 6,7, 3H), 1.54 (s, 9H), 1.17 (d, *J* = 6.9, 3H); ^1^H NMR (400 MHz, 1% in toluene-d_8_/Dioxane-d_8_) δ 7.61–7.56 (m, 3H), 7.51 (d, *J* = 7.7 Hz, 1H), 7.28–7.17 (m, 4H), 7.10–6.96 (m, 5H), 5.95 (d, *J* = 8.3 Hz, 1H), 5,21, 3.94 (AX system, *J* = 10.8 Hz, 2H), 5.27–5.19 (m, 1H), 4.45, 4.36, 4,11 (ABX system, *J =* 17.5, 7.0, 6,7, 3H), 1.54 (s, 9H), 1.17 (d, *J* = 6.5, 3H); ^13^C NMR (100.7 MHz, 1% in toluene-d_8_/Dioxane-d_8_) δ 172.1, 169.2, 168.1, 158.1, 155.9, 144.3, 144.1, 141.47, 141.44, 131.5, 128.6–125.0, 119.8, 83.2, 66.4, 60.8, 52.5, 48.9, 47.3, 27.5, 17.5; MS (ESI+): *m*/*z* calcd for [C_34_H_32_N_4_NaO_7_]: 631.64; found: *m*/*z* 631.20.

Fare anche questo

**3a*S*,6a*S-*5′b**: 44%. Mp 116.3 °C, dec.; [α]_D_^20^ +0.9 (*c* 1.00, CH_2_Cl_2_). ^1^H NMR (400 MHz, CDCl_3_) δ 7. 78 (d, *J* = 7.9 Hz, 2H), 7.75–7.59 (m, 2H), 7.51–7.26 (m, 9H), 5.76, 4.89 (AX system, *J* = 10.6 Hz, 2H), 5.69 (d, *J* = 8.2 Hz, 1H), 5.28–5.14 (m, 1H), 4.44–4.35 (m, 2H), 4.28–4.19 (m, 1H), 1.61 (s, 9H), 1.46 (d, *J* = 6.6, 3H); ^13^C NMR (100.7 MHz, CDCl_3_): δ 172.1, 168.7, 168.1, 158.3, 155.3, 144.4, 143.9, 143.8, 141.3, 141.28, 130.9, 129.3, 129.2, 127.7, 127.1, 126.3, 125.2, 119.9, 85.0, 67.1, 61.1, 52.9, 49.2, 47.2, 28.0, 19.0. ^1^H NMR (400 MHz, 1% in C_6_D_6_) δ 7.51 (d, *J* = 7.7 Hz, 2H), 7.41 (d, *J* = 6.3 Hz, 1H), 7.36 (d, *J* = 6.3 Hz, 1H),7.20–7.12 (m, 4H), 7.05–6.85 (m, 5H), 5.28–5.12 (m, 1 H), 5.21 (d, *J* = 8.0 Hz, 1H), 5.04, 3.84 (AX system, *J* = 10.2 Hz, 2H), 4.37–4.25 (m, 2H), 4.05–3.98 (m, 1H), 1.54 (s, 9H), 1.17 (d, *J* = 6.9, 3H); MS (ESI+): *m*/*z* calcd for [C_34_H_32_N_4_NaO_7_]: 631.64; found: *m*/*z* 631.24.

tButyl 3aR*,6aR*-1-(Fmoc-alanyl)-4,6-dioxo-5-benzyl-1,3a,4,5,6,6a-hexahydropyrrolo [3,4-c]pyrazole-3-carboxylate (**5c/5′c**): Method B; column chromatography: hexane 100% to hexane/AcOEt, 7:3.

**3a*R*,6a*R-*5c:** 42%. Mp 159.4 °C, dec.; [α]_D_^20^ −1.9 (*c* 1.00, CHCl_3_). ^1^H NMR (300 MHz, CDCl_3_) δ 7.75 (d, *J* = 7.3 Hz, 2H), 7.64–7.58 (m, 2H), 7.43–7.26 (m, 9H), 5.58, 4.61 (AX system, *J* = 10.5 Hz, 2H), 5.53 (d, *J* = 8.1 Hz, 1H), 5.17–5.05 (m, 1H), 4.63 (s, 2H), 4.42–4.26 (m, 2H), 4.42–4.26 (m, 1H), 1.58 (s, 9H), 1.47 (d, *J* = 7.4 Hz, 3H); ^13^C NMR (75 MHz, CDCl_3_) δ 172.5, 170.0, 168.8, 158.3, 155.7, 144.6, 143.9, 143.7, 141.3, 141.2, 134.5, 129.1, 128.8, 128.5, 127.7, 127.1, 125.2, 125.1, 120.0, 84.8, 67.1, 60.8, 52.7, 48.8, 47.1, 43.5, 27.9, 18.6; MS (ESI): *m*/*z* calcd for [C_35_H_35_N_4_O_7_] 623.69; found: *m*/*z* 623.03, 645.78 [M+Na].

**3a*S*,6a*S-*5c’:** 44%. Mp 138.6°C, dec.; [α]_D_^20^ +1.5 (*c* 1.00, CHCl_3_). ^1^H NMR (300 MHz, CDCl_3_) δ 7.76 (d, *J* = 7.4 Hz, 2H), 7.64–7.57 (m, 2H), 7.44–7.26 (m, 9H), 5.68 (d, *J* = 7.8 Hz, 1H), 5.52, 4.65 (AX system, *J* = 10.3 Hz, 2H), 5.20–5.06 (m, 3H), 4.63 (s, 2H), 4.40–4.30 (m, 2H), 4.26–4.17 (m, 1H) 1.58 (s, 9H), 1.40 (d, *J* = 6.7 Hz, 3H); ^13^C NMR (100.7 MHz, CDCl_3_): δ 171.9, 169.4, 168.6, 158.3, 157.2, 155.3, 144.2, 143.8, 141.28, 141.26, 137.5, 129.2, 128.8, 128.5, 127.7, 127.1, 125.2, 119.9, 84.9, 67.1, 60.9, 52.8, 49.1, 47.1, 43.5, 27.9, 18.9; MS (ESI+): *m*/*z* calcd for [C_35_H_34_N_4_NaO_7_]: 645.67; found: *m*/*z* 645.42.

Ethyl 3aR*,6aR*-1-(Boc-alanyl)-4,6-dioxo-5-benzyl-1,3a,4,5,6,6a-hexahydropyrrolo [3,4-c]pyrazole-3-carboxylate (**5d/5′d**): Method A; column chromatography: hexane 100% to hexane/AcOEt, 1:1.

**3a*R*,6a*R-*5d:** 38%; Mp 139.6 °C, dec.; [α]_D_^20^ −2.1 (*c* 1.00, CHCl_3_). ^1^H NMR (300 MHz, CDCl_3_) δ 7.36–7.23 (m, 5H), 5.60, 4.67 (AX system, *J* = 10.4 Hz, 2H), 5.17 (d, *J* = 8.4 Hz, 1H), 5.10–4.98 (m, 1H), 4.63 (s, 2H), 4.38 (q, *J* = 7.0 Hz, 2H), 1.42 (s, 9H), 1.40 (partially overl., d, 3H), 1.38 (t, *J* = 7.0 Hz, 3H); ^13^C NMR (75 MHz, CDCl_3_): δ 172.8, 169.9, 168.9, 159.5, 155.2, 134.5, 129.1, 128.8, 128.4, 79.9, 62.7, 60.9, 52.4, 48.2, 43.1, 28.3, 18.5, 14.0; MS (ESI+): *m*/*z* calcd for [C_23_H_28_N_4_NaO_7_]: 495.49; found: *m*/*z* 495.32.

**3a*S*,6a*S-*5d’:** 37%; Mp 140.5 °C, dec.; [α]_D_^20^ +1.9 (*c* 1.00, CHCl_3_). ^1^H NMR (300 MHz, CDCl_3_) δ 7.38–7.26 (m, 5H), 5.52, 4.69 (AX system, *J* = 10.3 Hz, 2H), 5.36 (d, *J* = 7.5 Hz, 1H), 5.16–5.01 (m, 1H), 4.62 (s, 2H), 4.38 (q, *J* = 7.1 Hz, 2H), 1.42 (s, 9H), 1.37 (t, *J* = 7.1 Hz, 3H); 1.33 (d, *J =* 7.25, 3H); ^13^C NMR (75 MHz, CDCl_3_): δ 172.4, 169.3, 168.8, 159.6, 142.4, 134.5, 129.2, 128.8, 128.5, 79.9, 62.8, 61.1, 52.6, 48.6, 43.7, 29.7, 28.3, 14.1; MS (ESI+): *m*/*z* calcd for [C_23_H_28_N_4_O_7_]: 472.50; found: *m*/*z* 472.20.

### 4.4. Gelation Test

Peptide powders were first weighed into a screw-capped glass vial (4 cm length and 1 cm diameter), as described in the literature [17]. An aliquot (0.5 mL) of different solvents was directly added onto the peptide powder to reach the desired concentration listed in Table 1 and Tables S1–S4 (Supporting Information). The mixture was vortexed for 2 min, sonicated for 20 min, and then heated in order to obtain a homogenous solution, before being left overnight at 25 °C. If not otherwise stated, the inversion test was carried out. The as-prepared material was preliminary classified as a “gel” if it did not exhibit gravitational flow upon turning the vial upside-down at room temperature. This state was further confirmed by oscillatory rheological measurements.

The morphological study of the gels was investigated by transmission electron microscopy at an accelerating voltage of 200 kV (JEOL-JEM-1200-EXII). Gel samples were diluted 2-fold, and they were deposited onto a TEM grid and then dried before imaging. First, **5b**-based gels were prepared using the method described above with benzene, *m*-xylene, and the toluene/dioxane mixture. To gain a visual insight into the gels’ structures, high-resolution SEM analysis (Mira3 Tescan, Czech Republic) was carried out. Small samples of gels were dried at high temperatures of 80–85 °C under vacuum. Samples were then mounted on the support and coated with graphite, and images were taken at a low voltage of 5 kV.

### 4.5. Rheology

The rheological behavior of organogels, prepared from **5b** (2% *w*/*v* of substrate) in toluene/dioxane (10:1), *m-*xylene, and benzene, was analyzed with the Rheometer Kinexus Plus (Malvern, Alfatest, Milano, Italy) with a circulated environmental system for temperature control.

The gel prepared from **5b** (20 mg) in the solvent (1 mL) was loaded (around half the amount) with a spatula with a plate–plate geometry of PU20:PL61 (20 mm upper-plate diameter) and a gap of 1 mm. A solvent trap was used to minimize solvent evaporation during all tests. Data processing was recorded with rSpace software version 1.50 (Malvern Panalytical, Monza, Italy)..

The viscoelastic properties were assessed by measuring the storage modulus (G′) and loss modulus (G″), representing the elastic and viscous behaviors of the material, respectively. The amplitude sweep test was carried out at a constant frequency of 1 Hz with a shear strain (%) from 0.001 to 100% at 25 °C to determine if the material had a linear viscoelastic region, and for which stress values the structure began to break down.

Frequency sweep tests were performed to measure the viscoelastic responses of the samples at 25 °C in an oscillation mode (10–0.1 Hz) at constant stress (0.5 Pa). The results show the viscoelastic responses of the samples at different frequencies.

All tests were performed in triplicate, and mean values are reported in logarithmic graphs (sd < 5%).

### 4.6. Determination of the Thermal Gel-to-Sol Transition Temperature (Tgel)

T_gel_ values were usually determined by the inverse flow method. A sealed vial containing the organogel was soaked into an oil bath, which was heated up at 2 °C/min. Tgel was defined as the temperature at which the gel started to break. Each measurement was made in duplicate and the average values are reported. The thermal gel-to-sol transition was evaluated by rheological analysis (PU20:PL61; 20 mm upper-plate diameter with a gap of 1 mm). Then, the 5b organogel (0.5 mL) was transferred with a spatula onto the lower plate at RT. The temperature was increased from 25 to 70 °C at 2 °C/min. Oscillation frequency and strain percentage were kept constant at 1 Hz and 0.01% strain (within the LVER). The mean values of G′ and G″ are reported in logarithmic graphs against the temperature (sd < 5%).

### 4.7. Phase-Selective Gelation (PSG)

A weighed amount of the corresponding peptide (20 mg) was added to a biphasic mixture of water and an appropriate organic solvent, changing the concentration according to Table 3. The selective gelation of the organic phase was tested in situ with shaking (*Method A*) or without shaking (*Method B*). The presence of residual aromatic solvent not undergoing gelation was assessed by pipetting the liquid, and the relative volume was measured. Then, gelation was verified by the complete absence of gravitational flow upon turning the vial upside-down.

## Figures and Tables

**Table 1:** Thermoreversibility of **5b** gels (2%, *w*/*v*) in different aromatic solvents.

Entry	Solvent	T_Gel_ (°C)
1	C_6_D_6_	92–96
2	*m-*Xylene	135–140
3	toluene/dioxane, 10:1	60–65

**Table 2:** PSG studies of **5b** in water with different aromatic solvents and concentrations.

Entry	Mixture	5b (%)	μL of Residual Organic Solvent		Observations
			A *^a^*	B *^b^*	
1	Toluene/Dioxane (10:1) (500 μL)	2%	150 μL	220 μL	Organic phase: partially gelled Water phase: solution
	H_2_O (500 μL)				
2	Benzene (500 μL)	2%	230 μL	250 μL	Organic phase: partially gelled Water phase: solution
	H_2_O (500 μL)				
4	Benzene (250 μL)	4%	20 μL	*^c^*	Organic phase: partially gelled (A)/gelled (B) Water phase layer: solution
	H_2_O (500 μL)				
5	Benzene (100 μL)	10%	*^c^*	*^c^*	Organic phase: completely gelled Water phase: solution
	H_2_O (500 μL)				
6	*m*-Xylene (500 μL)	2%	230 μL	250 μL	Organic phase: partially gelled Water phase: solution
	H_2_O (500 μL)				
7	*m*-Xylene (250 μL)	4%	20 μL	*^c^*	Organic phase: partially gelled (A)/gelled (B) Water phase: intact in liquid state
	H_2_O (500 μL)				
8	*m*-Xylene (100 μL)	10%	*^c^*	*^c^*	Organic phase: gelled Water phase: solution
	H_2_O (500 μL)				

*^a^ Method A:* without shaking; *^b^ Method B:* with shaking. *^c^*Organic solvent completely trapped in the gel.

**Table 3:** ^1^H NMR chemical shifts of **5b** (1% *w*/*v*) in different solvents at 298 K.

Solvent	H*_3a_*	H_6*a*_	NH_Ala_	CH_Ala_	Me_Ala_	CH_Fmoc_	CH_2Fmoc_	*t*Bu	Aromatic Protons
CDCl_3_	4.83	5.82	5.55	5.20	1.55	4.25	4.45–4.30	1.61	Fmoc: 7.75, 7.60, 7.42, 7.57; Ph: 7.63–7.26
CDCl_3_/ C_6_D_6_ *^a^*	4.00	5.17	5.33	5.14	1.32	4.18	4.45–4.30	1.53	Fmoc: 7.67, 7.56, 7.31, 7.27; Ph: 7.28–7.10
C_6_D_6_*^b^*	3.79	5.16	5.18	5.33	1.17	4.22	4.62–4.41	1.54	Fmoc: 7.70 (2H), 7.65 (1H) 7.57 (1H), 7.37–7.24 (4H); Ph: 7.17–6.98
C_6_D_6_ *^c^*	3.69	5.06	5.10	5.22	1.07	4.10	4.44/4.35	1.54	Fmoc: 7.57 (2H), 7.54 (1H), 7.46 (1H), 7.25–7.10 (4H); Ph: 7.03–6.88
C_6_D_6_ *^d^*	3.82	5.09	5.13	5.18	1.13	4.11	4.44/4.36	1.60	Fmoc: 7.58 (2H), 7.52 (1H), 7.48 (1H), 7.23–7.13 (4H); Ph: 7.06–6.92
toluene*_d8_*/ dioxane*_d8_ ^e^*	3.94	5.21	5.95	5.23	1.26	4.11	4.45/4.36	1.49	Fmoc: 7.61–7.56 (3H), 7.51 (1H), 7.28–7.17 (4H); Ph: 7.10–6.96
toluene*_d8_*/ dioxane-*_d8_ ^f^*	3.91	5.18	6.02	5.23	1.26	4.11	4.45/4.36	1.48	Fmoc: 7.60–7.55 (3H), 7.51 (1H), 7.27–7.15 (4H); Ph: 7.11–6.95
toluene*_d8_*/ dioxane*_d8_ ^g^*	4.02	5.19	5.54	5.15	1.26	4.11	4.42/4.35	1.47	Fmoc: 7.60–7.56 (2H), 7.54 (1H), 7.51 (1H), 7.20–7.14 (4H); Ph: 7.11–6.97

*^a^* 1:1. *^b^* 1% *w*/*v* (25 °C). *^c^* 2% *w*/*v* (25 °C). *^d^* 2% *w*/*v* (70 °C). *^e^* (10:1), 1% *w*/*v* (25 °C). *^f^* (10:1), 2% *w*/*v* (25 °C). *^g^* (10:1), 2% *w*/*v* (60 °C).

## Data Availability

The data presented in this study are openly available in article.

## Supplementary Materials

The following supporting information can be downloaded at: https://www.mdpi.com/article/10.3390/gels10040263/s1. Figure S1: Chemical shifts, NOEs (arrow) and H-bond (dash) for 5b (400 MHz; 298 K) in different solvents; Figure S2: 1H NMR of compound 5b in CDCl3 (1%, 400 MHz) at variable temperature (273–328 K); Figure S3: 1H NMR of a freshly prepared sample of 5b in C6D6 (2%, 400 MHz) at variable temperature (298–343K); Figure S4: 1H NMR of a sample of 5b prepared the day before in C6D6 (2%, 500 MHz) at variable temperature (298–343K); Figure S5: 1H NMR of a sample of 5b prepared the day before in C6D6 (2%, 500 MHz), heated (as reported in the legend of FS4), then cooled from 343 to 283 K; Figure S6: 1H NMR of a sample of 5b prepared for experiments as reported in the legends of FS4 and FS5, then let it at 298 for 24 h; Figure S7: 1H NMR spectra at variable temperature (298–363K) of a sample of 5b in toluene-d8/diossane-d8 (10:1; 2%, 500 MHz), recorded after 48 h after the sample preparation.; Figure S8: Noesy of compound 5b (1% CDCl3, 293 K, 400 MHz, 600 ms); Figure S9: Noesy of compound 5b (1% CDCl3/C6D6, 1:1; 400 MHz) 600 ms; Figure S10: Noesy of compound 5b (1% C6D6, 400 MHz) (A) 800 ms and B) zoom of MeAla region (500 ms); Figure S11: A) Noesy of compound 5b in 1% tolene-d8/dioxane-d8 (10:1; 400 MHz; 900 ms); (B) zoom of CH/NH region; Figure S12: Chemical shifts, NOEs (arrow) and H-bond (dash) for compound 5′b: main conformer I-5′b in CDCl3 and the mixture of conformers II-5′b (main conformer) and I-5′b (minor conformer) in C6D6 (400 MHz; 283 K). The Noesy experiment in C6D6 was detected at 298 K (500 ms); Figure S13: 1H NMR of compound 5′b in CDCl3 (1%, 400 MHz) at variable temperature (273–328 K); Figure S14: 1H NMR of compound 5′b in C6D6 (2% 400 MHz) at variable temperature (273-323K); Figure S15: (A) Noesy of compound 5′b (1% CDCl3, 400 MHz, 300 ms); (B) zoom of Me/CH,NH region; Figure S16: Noesy of compound 5′b (1% C6D6, 400 MHz) at 500 ms and 300 K; Figure S17: Cosy of compound 5′b (1% C6D6, 400 MHz) at 283 K; Figure S18: NOESY of compound 5′b (1% C6D6, 500 MHz) at 500 ms and 283 K. Table S1: Gelation test on compound 4a–d (2%) at 25 °C (overnight) in different solvents; Table S2: Gelation test on compound 5a,c,d and 5′a,c,d (2%) at 25 °C (overnight) in different solvents; Table S3: Gelation test on compound 5b at 25 °C (overnight) in different solvents and concentrations; Table S4: Gelation test on compound 5′b (2%) at 25 °C (overnight) in different solvents; Table S5: Chemical shifts of a sample of 5b prepared the day before in C6D6 (2%, 500 MHz) at variable temperature: from 298 to 348 and from 348 to 283; Table S6: 1H NMR chemical shifts of 5′b (1% *w*/*v*) in different solvents at 298 K.
