# Supplementary material for: Stereochemical Behavior of Pyrrolo-Pyrazole Peptidomimetics Promoting Phase-Selective Supramolecular Organogels
Source: Gels. 2024 Apr 14;10(4):263. doi: 10.3390/gels10040263 (PMC11049432; doi:10.3390/gels10040263)

Stereochemical Behavior of Pyrrolo-pyrazole Peptidomimetics Promoting Phase-Selective Supramolecular Organogels

Enrica Chiesa [1], Francesco Anastasi [2], Francesca Clerici [2], Edoardo Mario Lumina [2], Ida Genta [1], Sara Pellegrino [2,*] and Maria Luisa Gelmi [2,*]

1. GELATION STUDIES **S2-S3**
2. NMR STUDIES ON 5b AND 5'b **S4-S15**
3. NMR SPECTRA OF COMPOUNDS 3, 4 AND 5/5' **S16-S38**

1. GELATION STUDIES

Table TS1. Gelation test on compound **4a-d** (2%) at 25 °C (overnight) in different solvents.

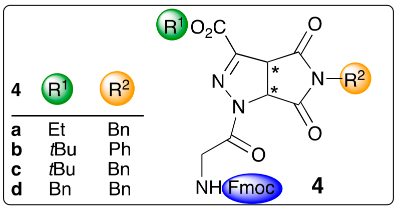

**Compound**	Hexane/AcOEt (6:4)	*t*-BuOMe	Toluene/Dioxane (10:1)	Toluene
**4a**	S	G with P	S	S
**4b**	S	S	S	S
**4c**	S/P	S/P	S/P	S
**4d**	S	I	S	S

G: gel, S: (viscous) solution, P: precipitate; I: insoluble

Table TS2. Gelation test on compound **5a,c,d** and **5’a,c,d** (2%) at 25 °C (overnight) in different solvents.

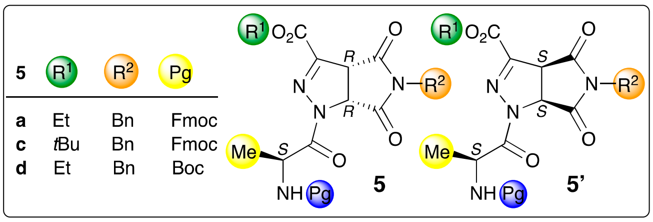

**Compound**	Hexane/AcOEt (6:4)	*t*-BuOMe	Toluene/Dioxane (10:1)	Toluene
**5a**	S/P	S/P	S/P	S/P
**5’a**	S/P	S/P	S/P	S/P
**5c**	S/P	S/P	S/P	S/P
**5’c**	S	I	S	S
**5d**	S/P	S/P	S	S/P
**5’d**	S/P	S/P	S/P	S/P

S: (viscous) solution, P: precipitate; I: insoluble

Table TS3. Gelation test on compound **5b** at 25 °C (overnight) in different solvents and concentrations.

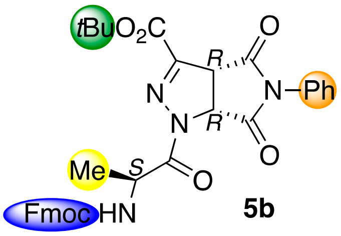

**Solvent (T)**	**% (w/v)**	**Status**	**% (w/v)**	**Status**	**% (w/v)**	**Status**
Hexane	2	PS				
C_6H_{12}	2	I				
Hexane/AcOEt (6:4)	2	G	2.5	PS	3	PS
Et_2O	1	G	2	G/PS	-	-
t-BuOMe	1	G	2	G	-	
AcOEt	2	S	-	-	-	-
THF	2	I	-	-	-	-
CH_2Cl_2	2	S	-	-	-	-
$CHCl_3$	2	S	-	-		
AcOEt	2	S/P	-	-	-	-
MeOH	2	I	-	-	-	-
Tetraline	2	P	2.5	P	3	PS

Toluene	2	S	-	-	-	-
Toluene/Dioxane (9:1)	1	G	2	G	2.5	P
Toluene/Dioxane (10:1)	1	S	2	G	-	-
Benzene	1.75	PS	1.9	PS	2	G
m-Xylene	1.5	S	1.75	S	2	G
Chloro-benzene	2	P	2.5	P	3	P

G: gel, S: (viscous) solution, PS partially soluble, P: precipitate; I: insoluble

Table TS4. Gelation test on compound **5'b** (2%) at 25 °C (overnight) in different solvents.

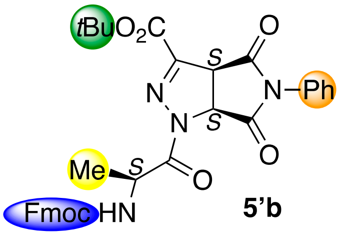

5'b

Solvent	Status
Hexane	I
Hexane/Acetat (6:4)	S
Diethyl ether	S
CH_2Cl_2	S
AcOEt	S
MeOH	S
Toluene	S
Toluene/Dioxane (10:1)	S
t-BuOMe	S
THF	S

S: (viscous) solution, I: insoluble

2. NMR STUDIES ON 5b AND 5'b

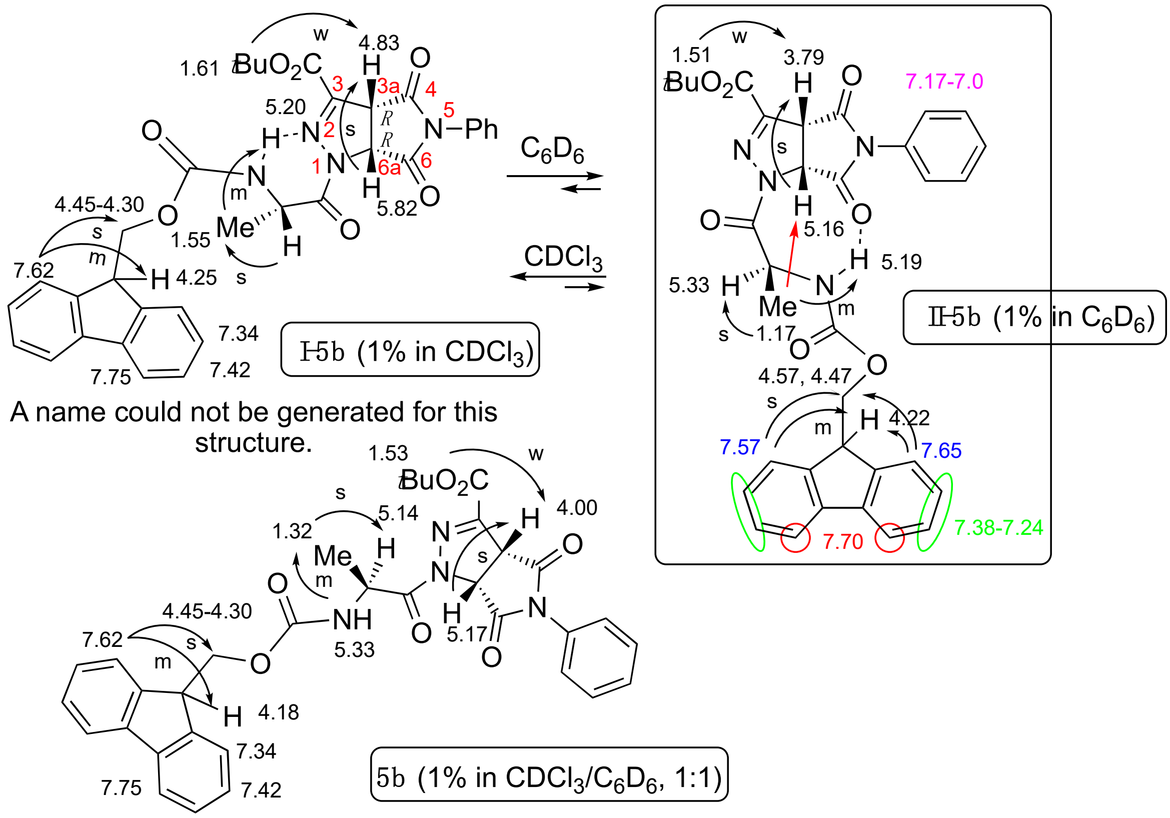

Figure S1. Chemical shifts, NOEs (arrow) and H-bond (dash) for **5b** (400 MHz; 298 K) in different solvents.

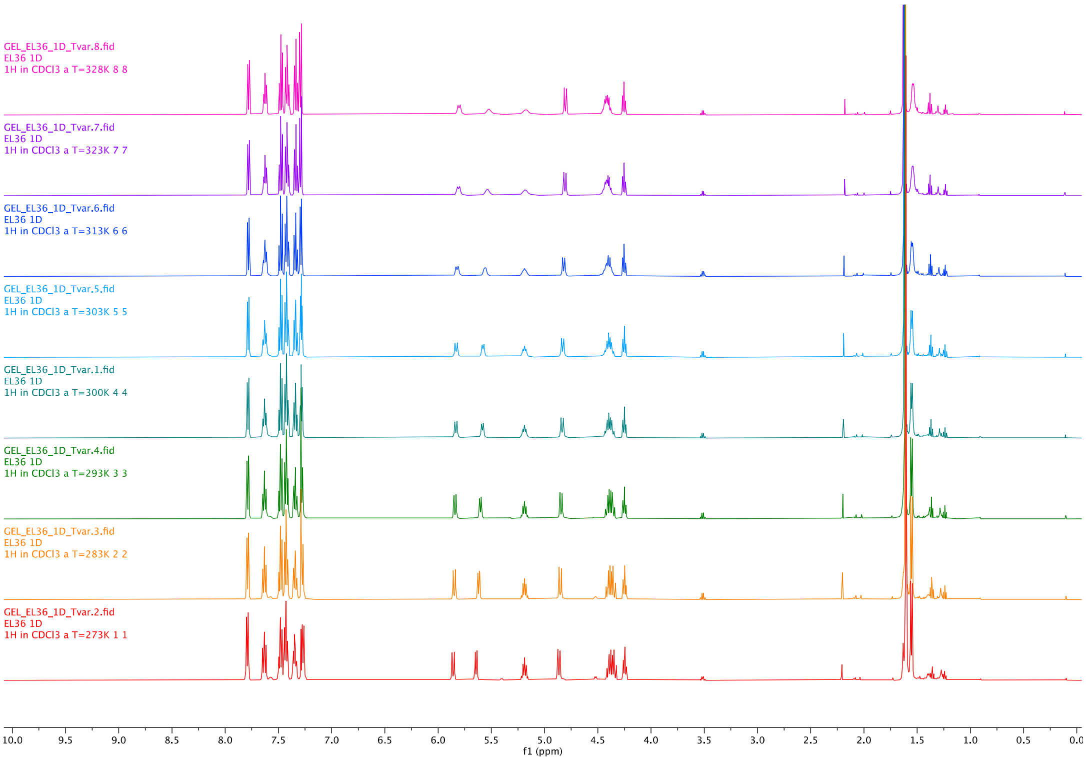

Figure S2. ^{1}H NMR of compound **5b** in $CDCl_3$ (1%, 400 MHz) at variable temperature (273-328 K)

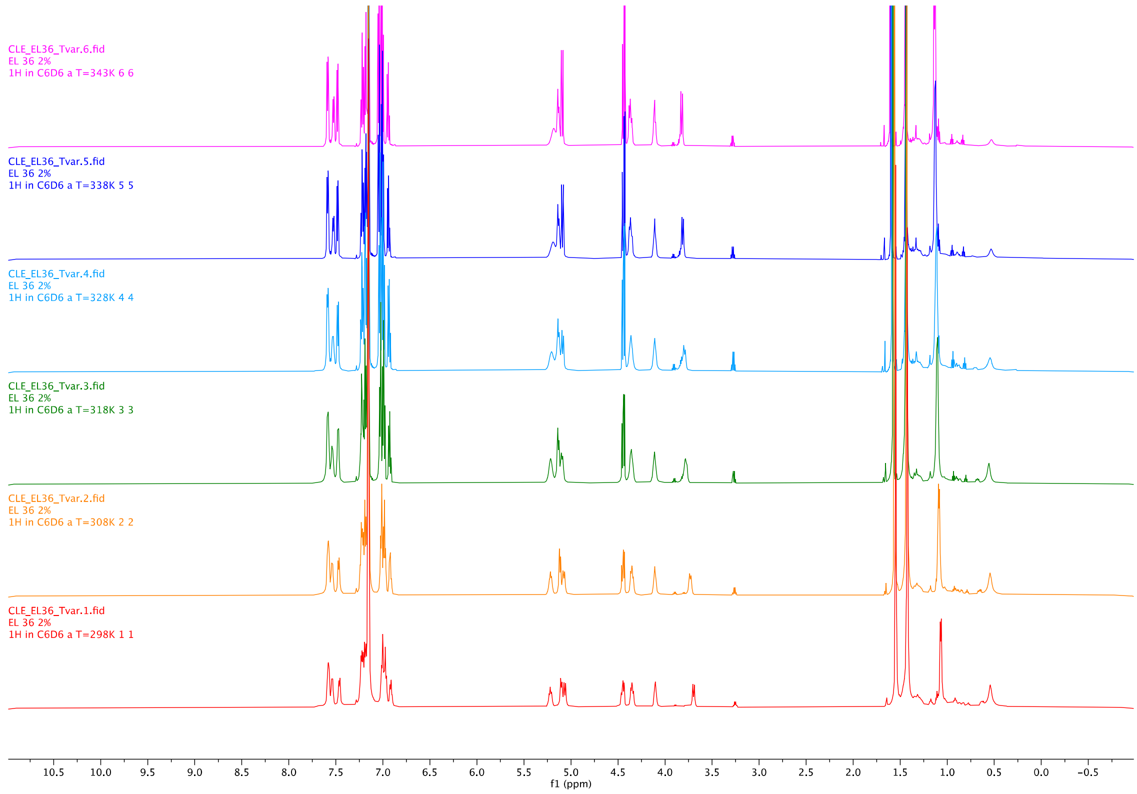

Figure S3. ^{1}H NMR of a freshly prepared sample of **5b** in C_6D_6 (2%, 400 MHz) at variable temperature (298-343K)

Table TS5. Chemical shifts of a sample of **5b** prepared the day before in C_6D_6 (2%, 500 MHz) at variable temperature: from 298 to 348 and from 348 to 283

T	H_{3a}	H_{6a}	NH_{Ala}	CH_{Ala}	Me_{Ala}	CH_{Fmoc}	CH_{2Fmoc}	*t*Bu	Aromatic protons
298	3.73 *J* 10.4	5.085 *J* 10.4	5.1 Overl.	5.21	1.07 *J* 6.6	4.09 (br t)	4.45 (dd) 4.35 (dd)	1.42	Fmoc: 7.57 (br t, 2H), 7.53 (d, 1H), 7.476 (d, 1H), 7.25-7.15 (4H); Ph: 7.02-6.97 (2H), 6.98-6.94 (2H), 6.92-6.88 (1H)
333	3.81 *J* 10.4	5.09 *J* 10.2	5.13 *J* 7.7	5.19	1.11 (br d)	4.10 (br)	4.44 (dd) 4.36 (br t)	1.43	Fmoc: 7.58 (br d, 2H), 7.52 (d, 1H), 7.48 (d, 1H), 7.24-7.15 (4H); Ph: 7.06-7.01b(2H), 7.01-6.97 (2H), 6.95-6.92 (1H)
343	3.86	5.09 *J* 10.	5.14 *J* 7.1	5.18	1.13 *J* 6.5	4.10 (t)	4.43 (dd) 4.36 (br dd)	1.44	Fmoc: 7.58 (br d, 2H), 7.52 (d, 1H), 7.48 (d, 1H), 7.24-7.15 (4H); Ph: 7.06-7.02 (2H), 7.01-6.97 (2H), 6.96-6.91 (1H)
348	3.87 *J* 10.4	—5.20-5.10—			1.14 *J* 6.3	4.10 (t)	4.43 (dd) 4.36 (br dd)	1.44	Fmoc: 7.57 (br d, 2H), 7.55 (d, 1H), 7.47 (d, 1H), 7.23-7.15 (4H); Ph: 7.03-6.98 (2H), 6.98-6.93(2H), 6.92-6.87 (1H)
333	3.85 *J* 10.8	5.12 *J* 10.4	5.14 *J* 8.1	5.19 brs	1.12 *J* 6.3	4.10 (brs)	4.43 (dd) 4.36 (brs)	1.43	Fmoc: 7.58 (br d, 2H), 7.52 (d, 1H), 7.48 (d, 1H), 7.23-7.15 (4H); Ph: 7.06-7.02(2H), 7.02-6.98 (2H), 6.95-6.91 (1H)
293	3.86 *J* 10.5	5.20 *J* 10.7	5.16 *J* 8.3	5.21 Overl.	1.08 *J* 6.7	4.09 (t)	4.44 (dd) 4.36 (dd)	1.42	Fmoc: 7.57 (br t, 2H), 7.55 (d, 1H), 7.47 (d, 1H), 7.25-7.15 (4H); Ph: 7.03-6.99 (2H), 6.99-6.94 (2H), 6.92-6.88 (1H)
283	3.89 (two d *J* 10.3)	5.24 *J* 10.7	5.18 *J* 7.5	5.23	1.07 *J* 5.6	4.09 (brs)	4.44 (t) 4.37 (t)	1.42	Fmoc: 7.57 (br t, 2H), 7.65 (d, 1H), 7.47 (d), 1H), 7.26,7.16 (m, 4H); Ph: 7.04-6.99 (2H), 6.99-6.94 (2H), 6.92-6.87 (1H)

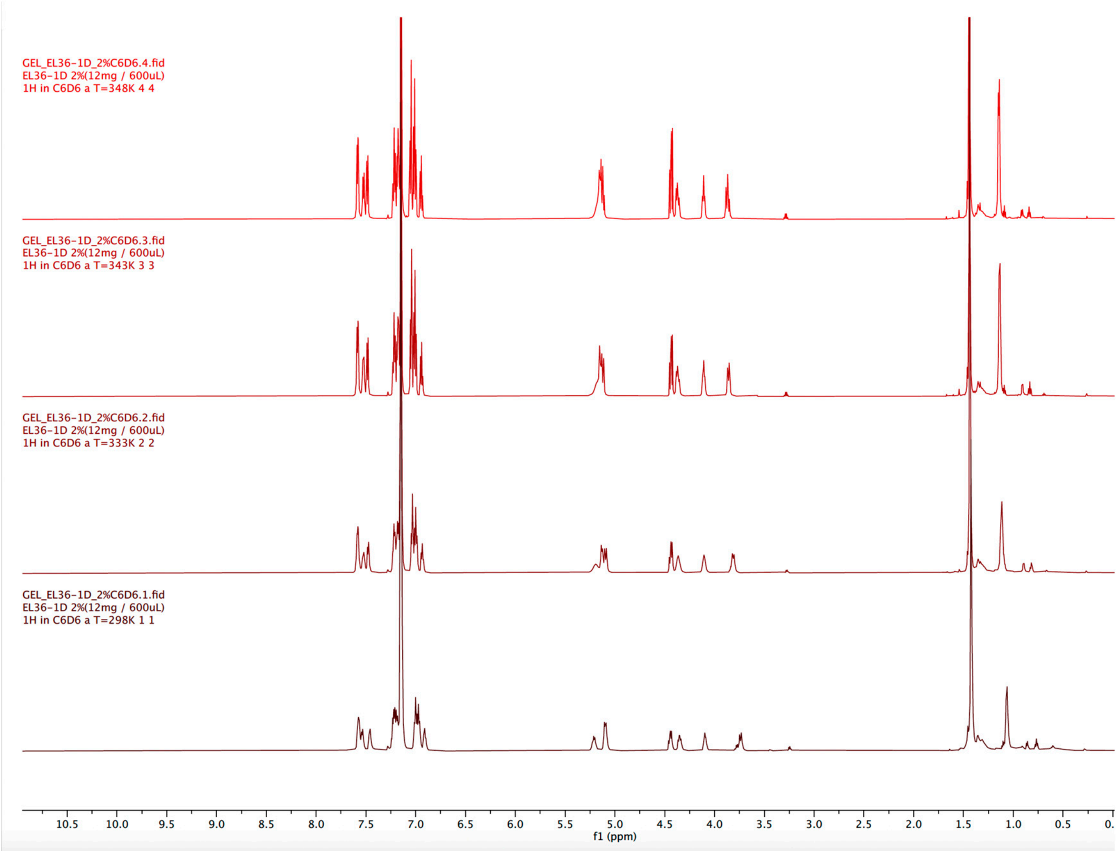

Figure S4. ^{1}H NMR of a sample of **5b** prepared the day before in C_6D_6 (2%, 500 MHz) at variable temperature (298-343K)

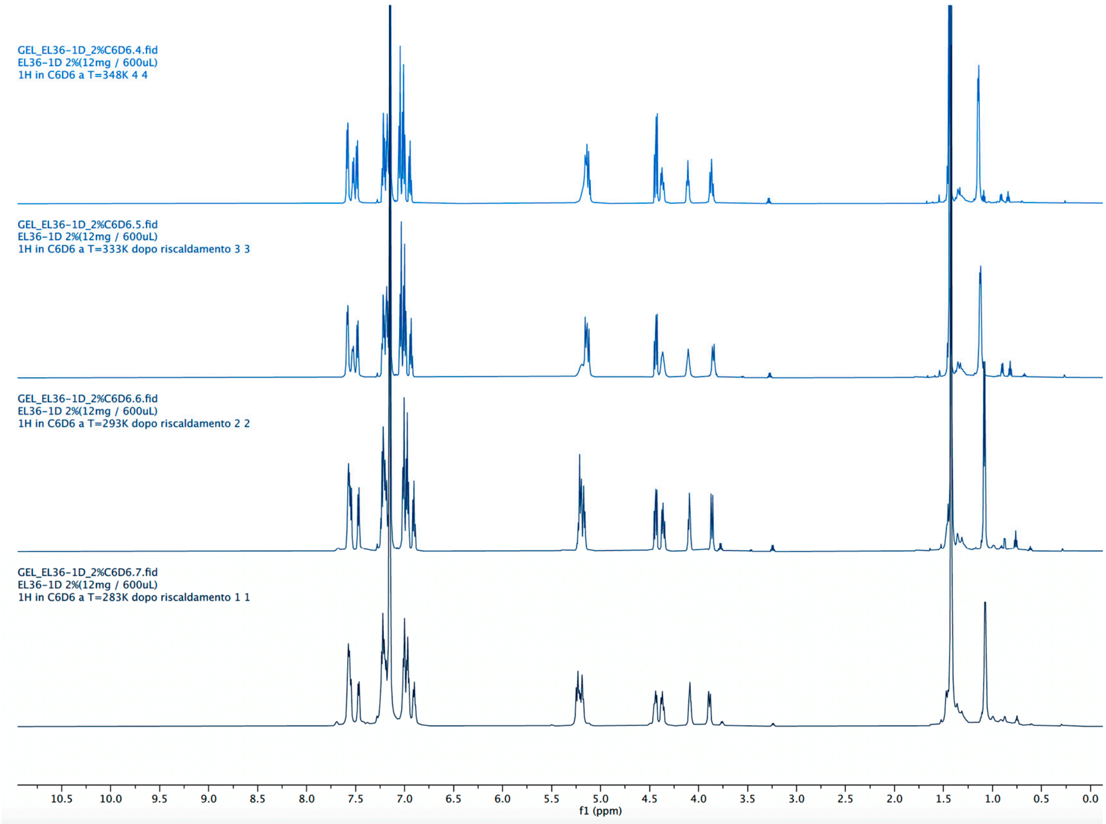

Figure S5. ^{1}H NMR of a sample of **5b** prepared the day before in C_6D_6 (2%, 500 MHz), heated (as reported in the legend of FS4), then cooled from 343 to 283 K.

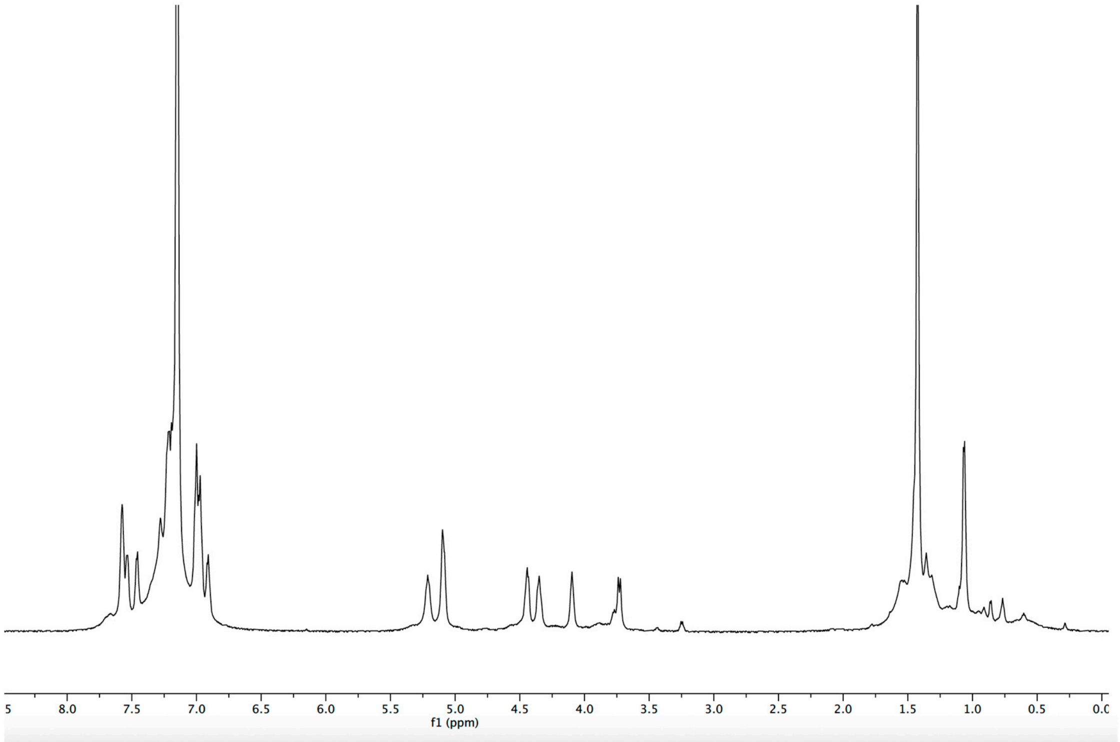

Figure S6. ^{1}H NMR of a sample of **5b** prepared for experiments as reported in the legends of FS4 and FS5, then let it at 298 for 24 h.

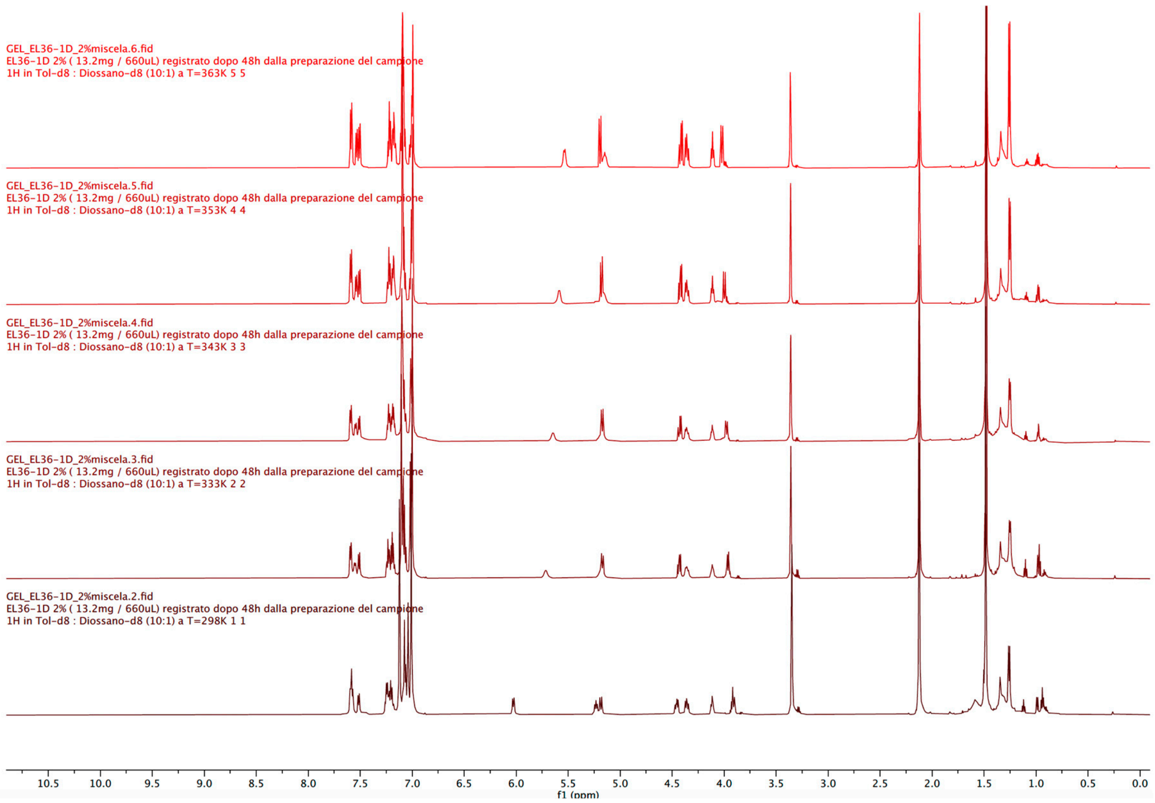

Figure S7. ^{1}H NMR spectra at variable temperature (298-363K) of a sample of **5b** in toluene-d_8/diossane-d_8 (10:1; 2%, 500 MHz), recorded after 48 h after the sample preparation.

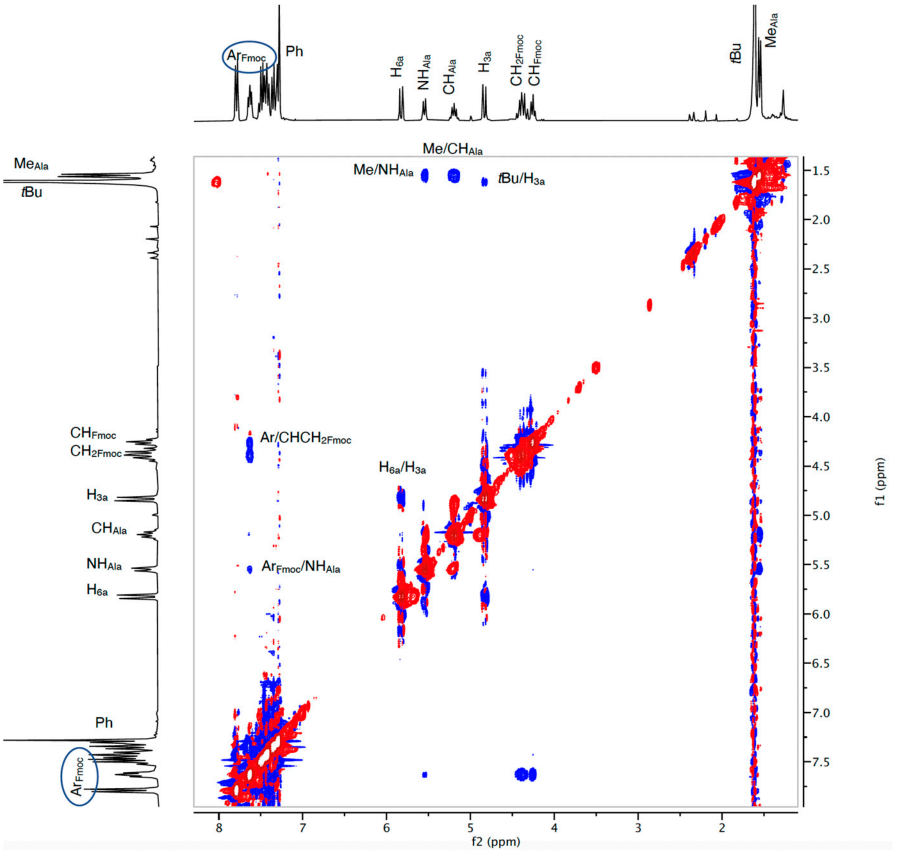

Figure S8. Noesy of compound **5b** (1% $CDCl_3$, 293 K, 400 MHz, 600 ms)

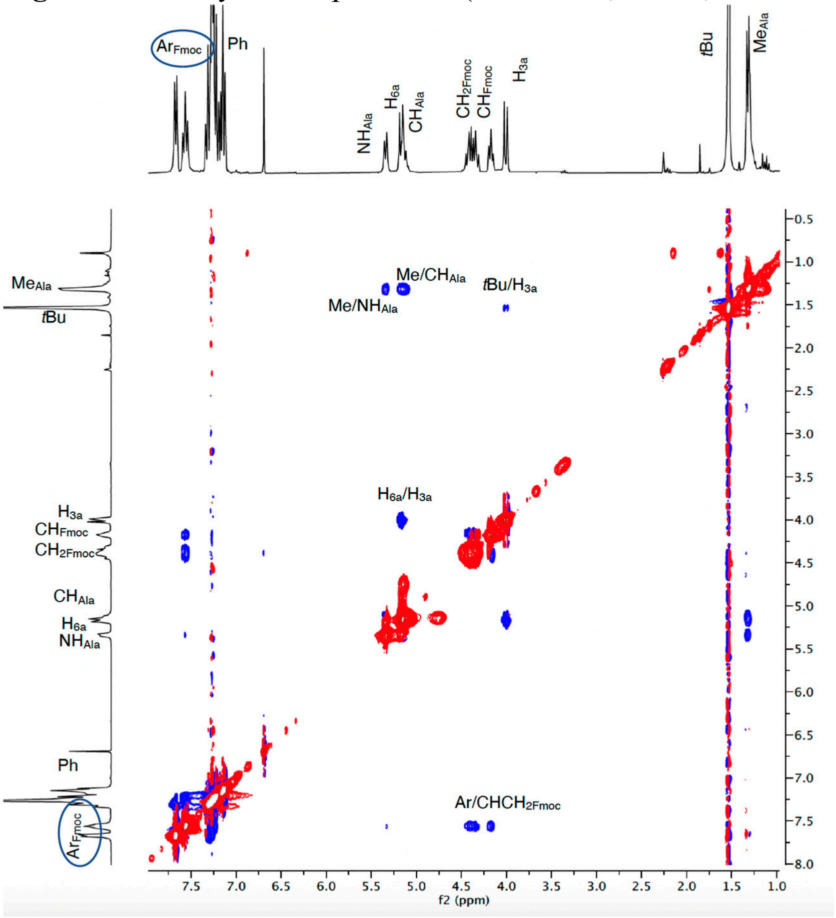

Figure S9. Noesy of compound **5b** (1% $CDCl_3/C_6D_6$, 1:1; 400 MHz) 600 ms

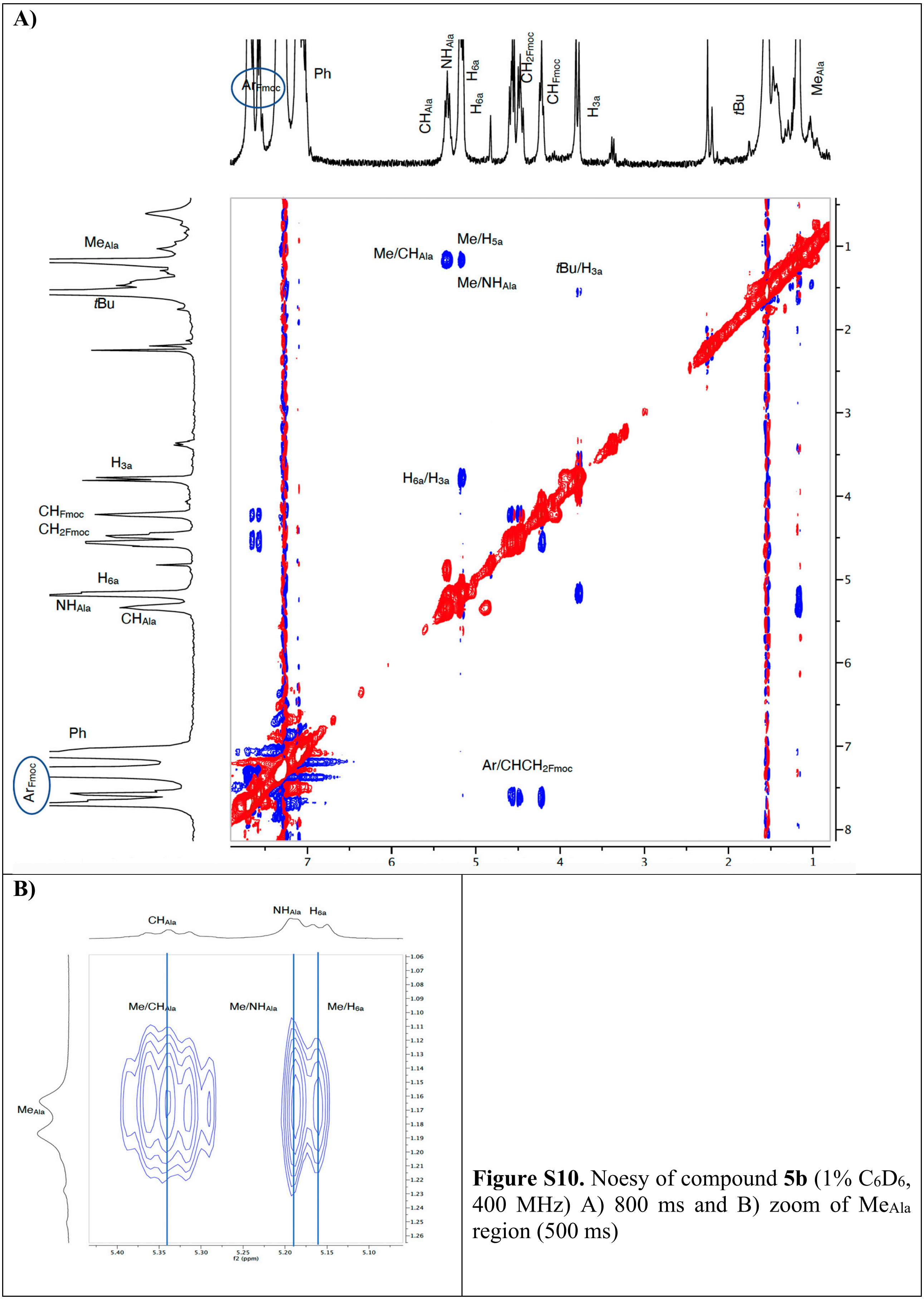

Figure S10. Noesy of compound **5b** (1% C_6D_6, 400 MHz) A) 800 ms and B) zoom of Me_{Ala} region (500 ms)

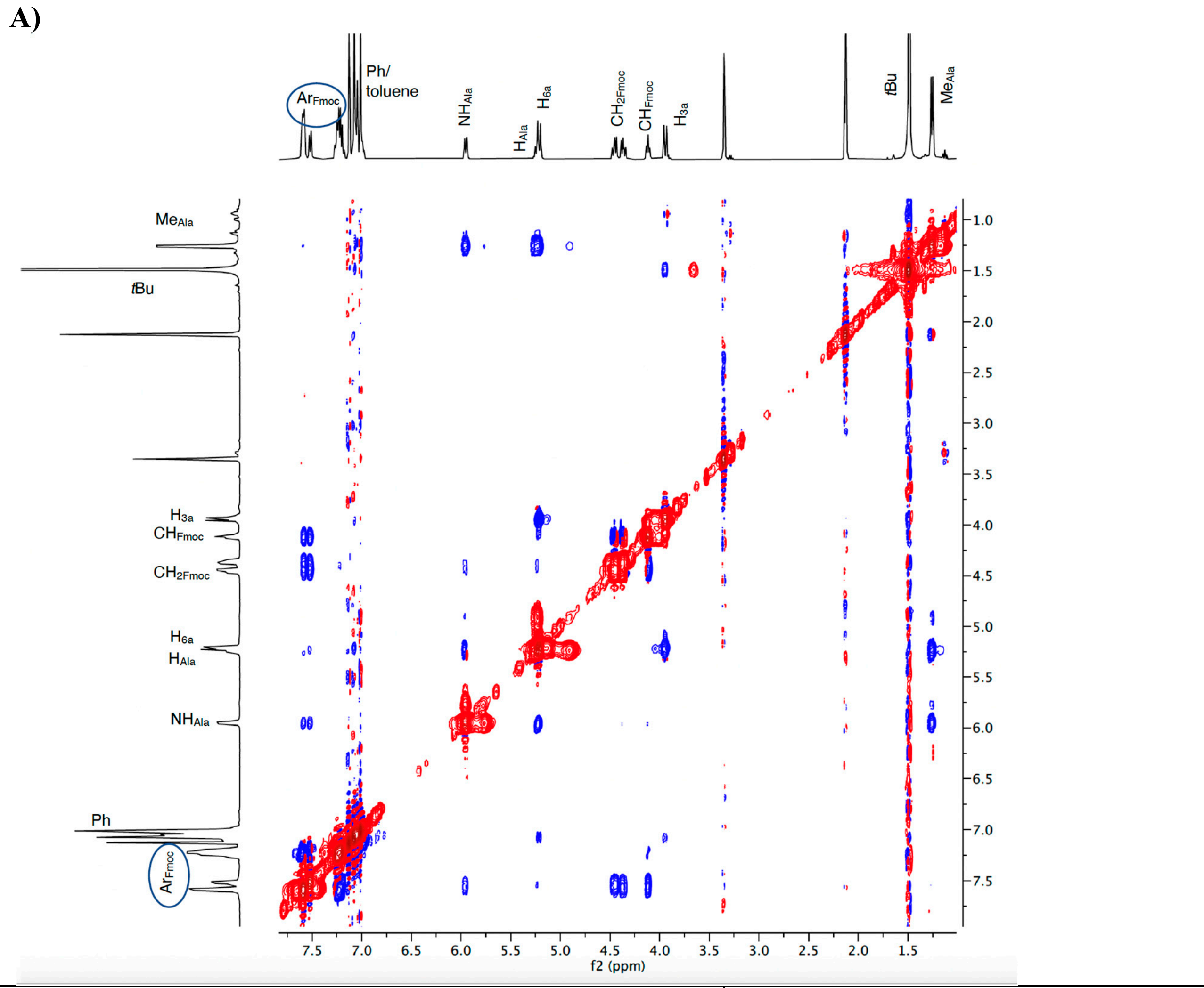

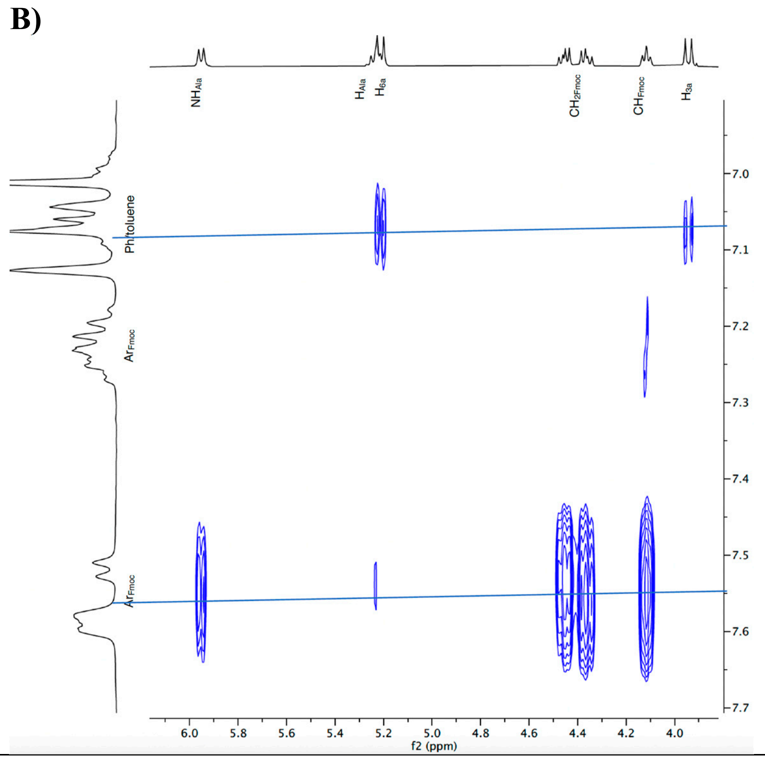

Figure S11. A) Noesy of compound **5b** in 1% tolene-d8/dioxane-d8 (10:1; 400 MHz; 900 ms); B) zoom of CH/NH region

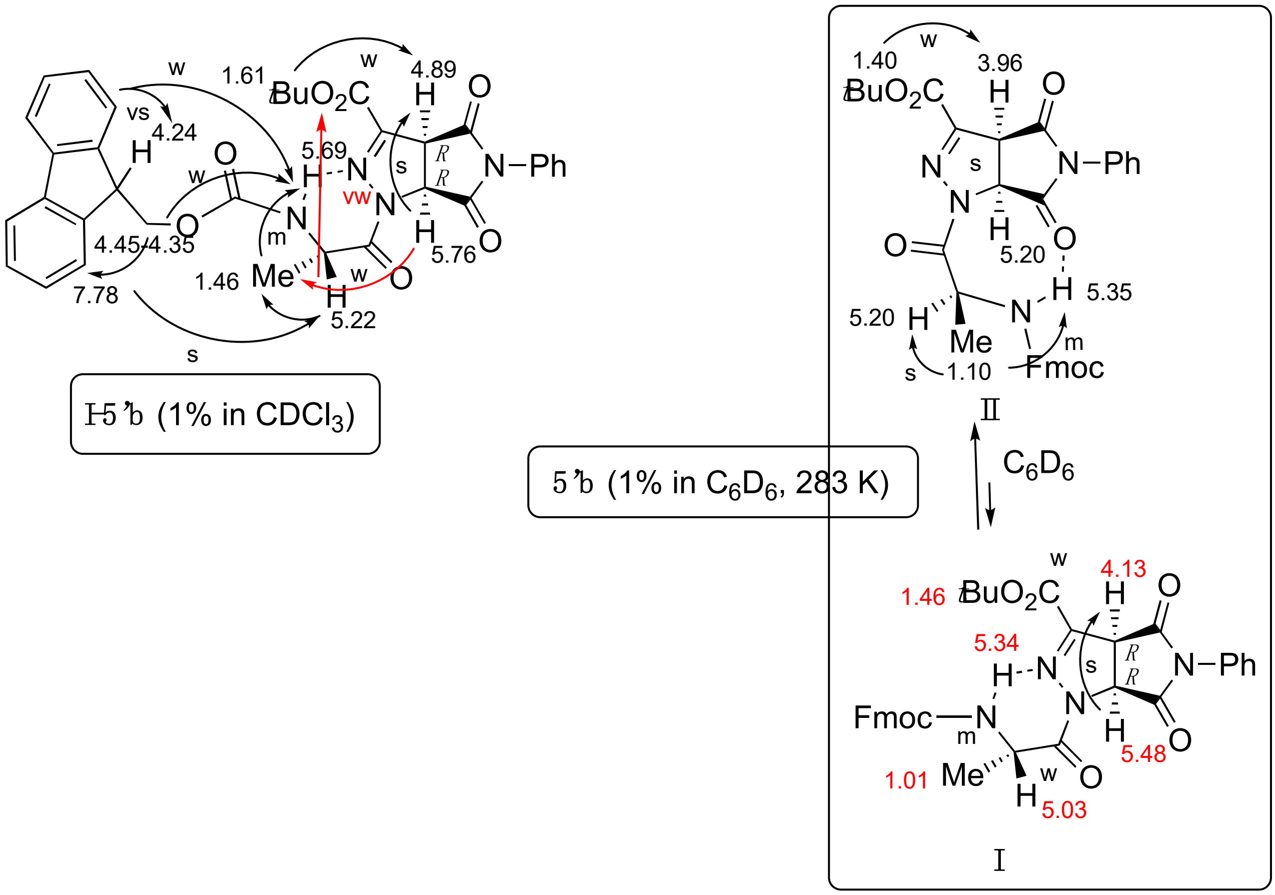

Figure S12. Chemical shifts, NOEs (arrow) and H-bond (dash) for compound **5'b**: main conformer **I-5'b** in $CDCl_3$ and the mixture of conformers **II-5'b** (main conformer) and **I-5'b** (minor conformer) in C_6D_6 (400 MHz; 283 K). The Noesy experiment in C_6D_6 was detected at 298 K (500 ms)

Table TS6. ^{1}H NMR chemical shifts of **5'b** (1% w/v) in different solvents at 298 K.

**Solvent**	H_{3a}	$H6_a$	NH_{Ala}	CH_{Ala}	Me_{Ala}	CH_{Fmoc}	CH_{2Fmoc}	*t*Bu	Aromatic protons
$CDCl_3$	4.89	5.76	5.69	5.22	1.46	4.24	4.45-4.35	1.61	Fmoc: 7.78, 7.62, 7.40, 7.34; Ph: 7.52-7.26
$C_6D_6{}^a$	3.90	5.09	5.27	5.21	1.13	4.01	4.36-4.22	1.41	Fmoc: 7.54 (2H), 7.45 (1H) 7.40 (1H), 7.24-7.06 (4H); Ph: 7.06-6.87

^{a}Conformer **I** at 298 K

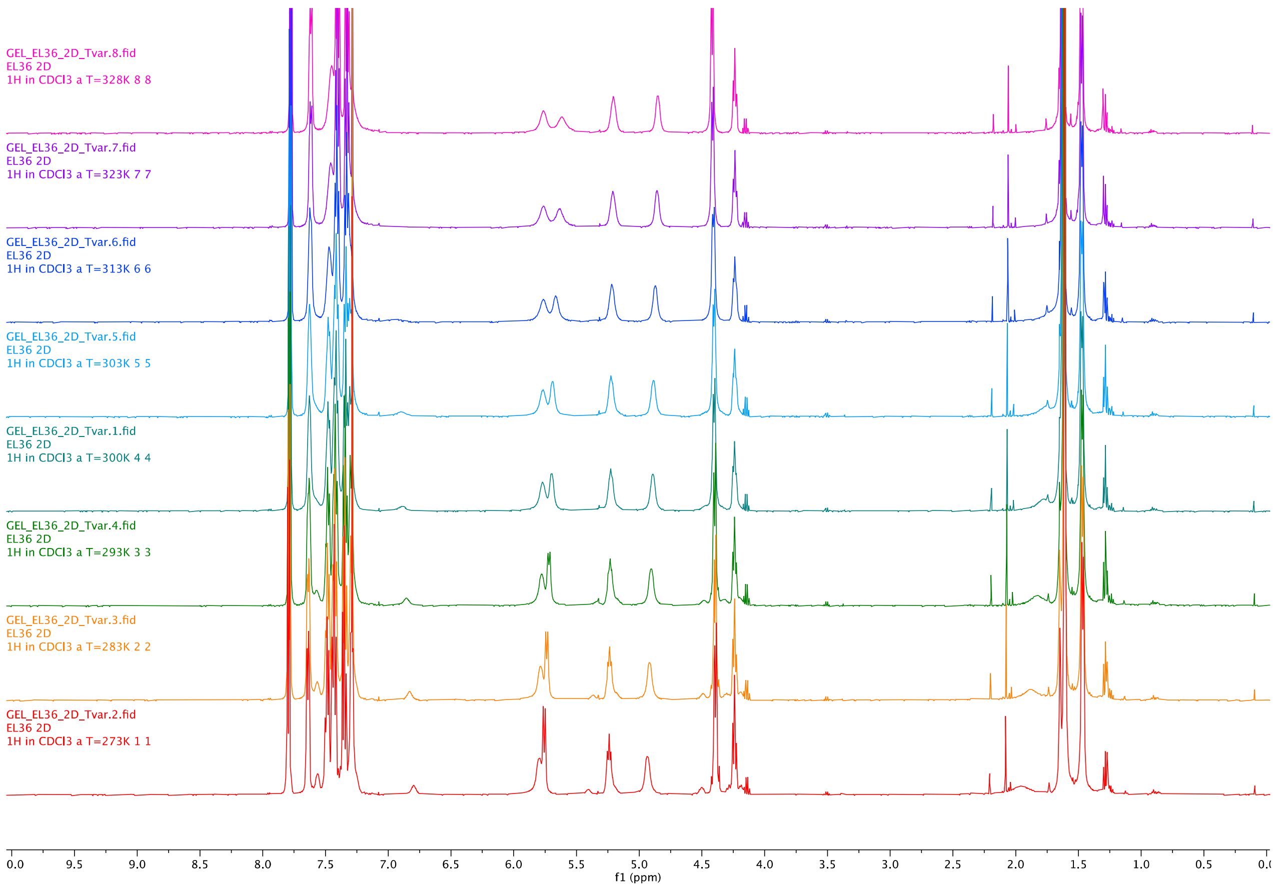

Figure S13. ^{1}H NMR of compound **5'b** in $CDCl_3$ (1%, 400 MHz) at variable temperature (273-328 K)

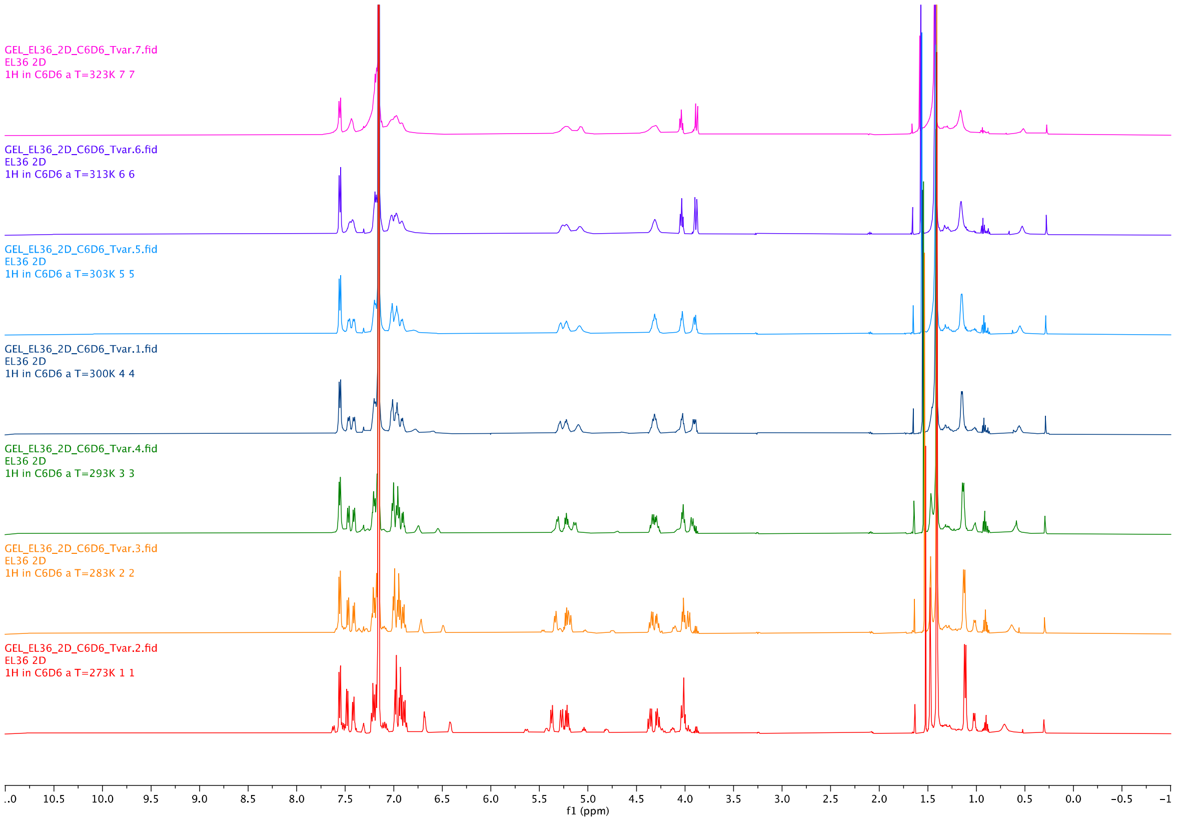

Figure S14. ^{1}H NMR of compound **5'b** in C_6D_6 (2% 400 MHz) at variable temperature (273-323K)

A)

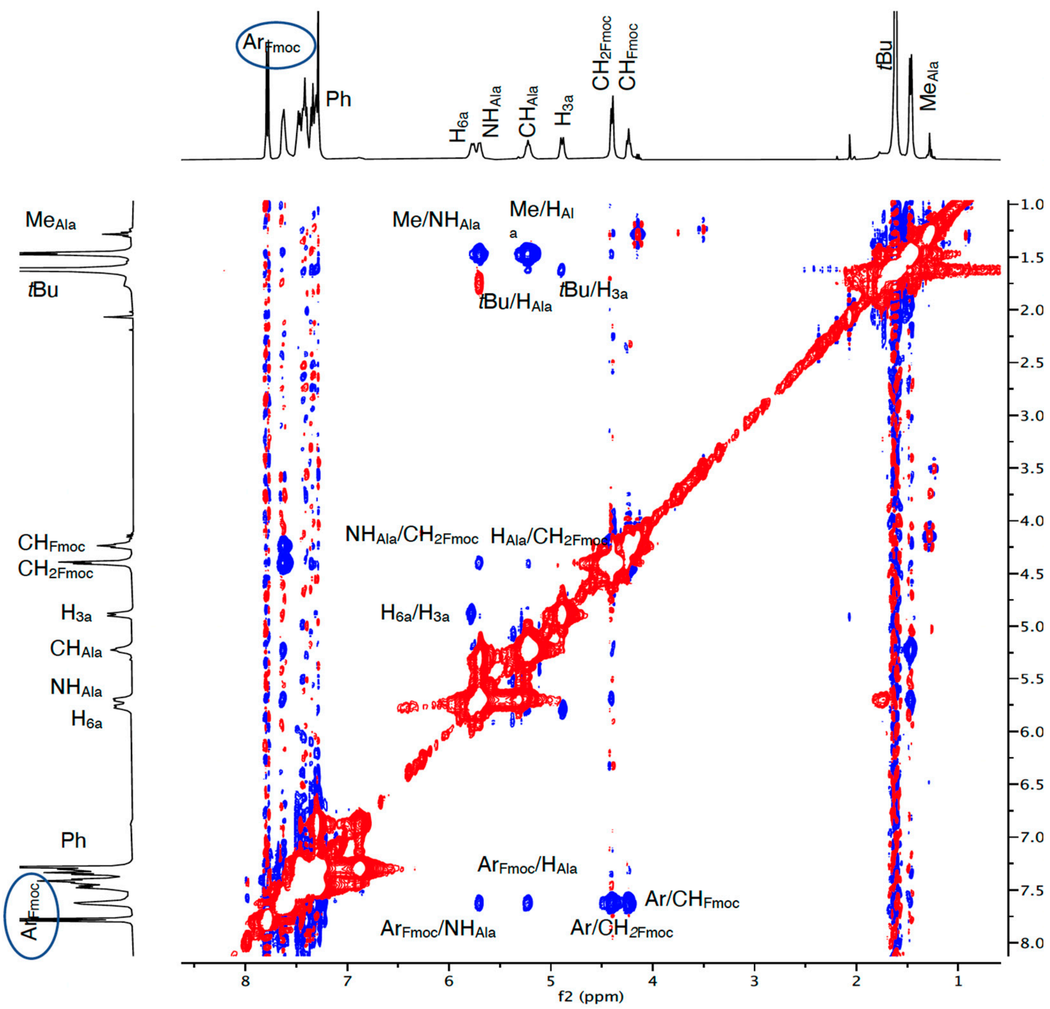

B)

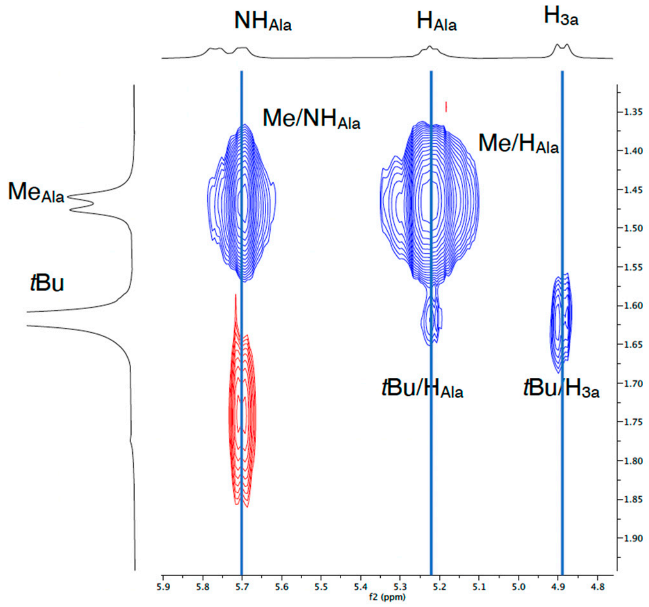

Figure S15. A) Noesy of compound **5'b** (1% $CDCl_3$, 400 MHz, 300 ms); B) zoom of Me/CH,NH region

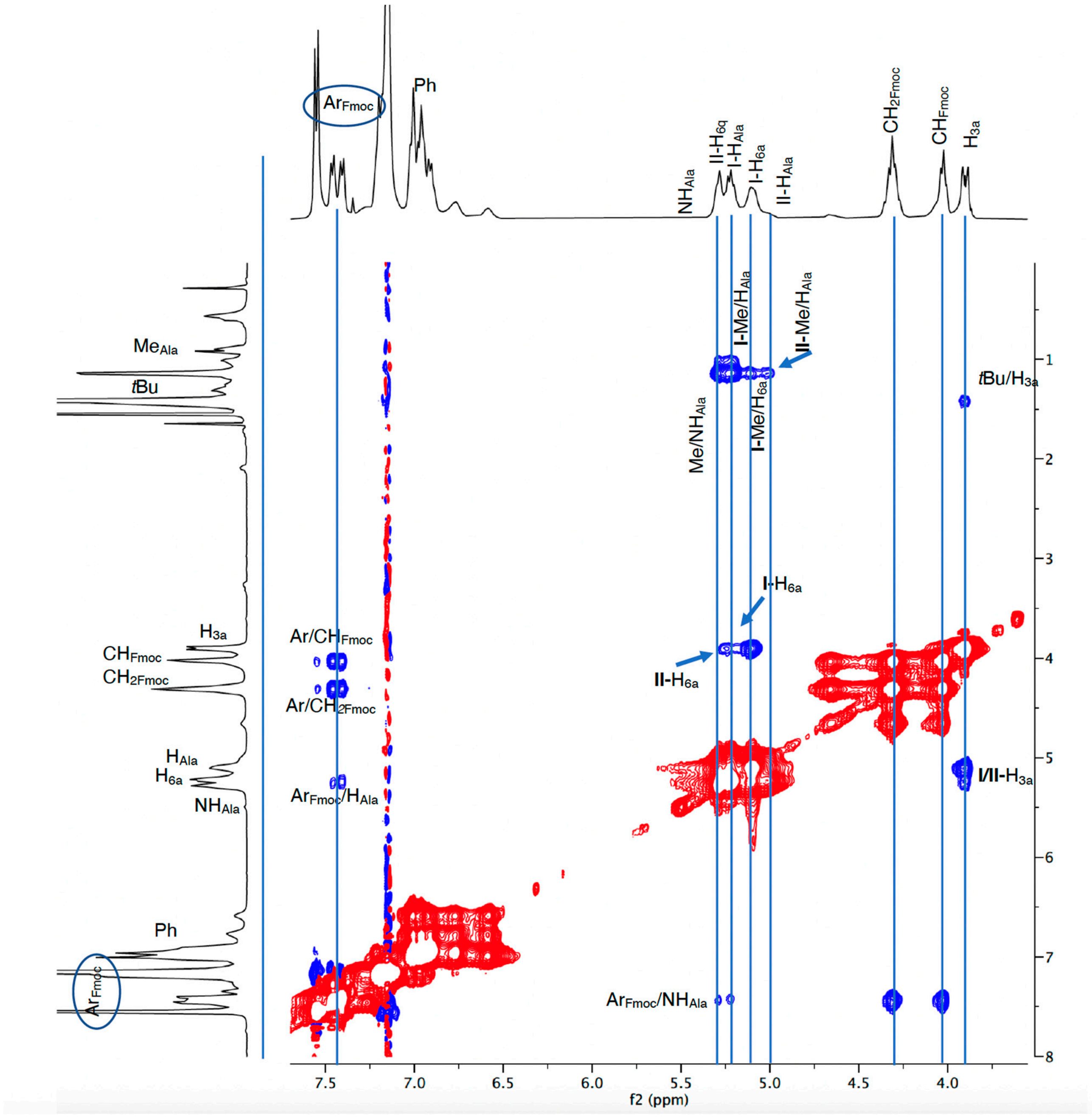

Figure S16. Noesy of compound **5'b** (1% C_6D_6, 400 MHz) at 500 ms and 300 K.

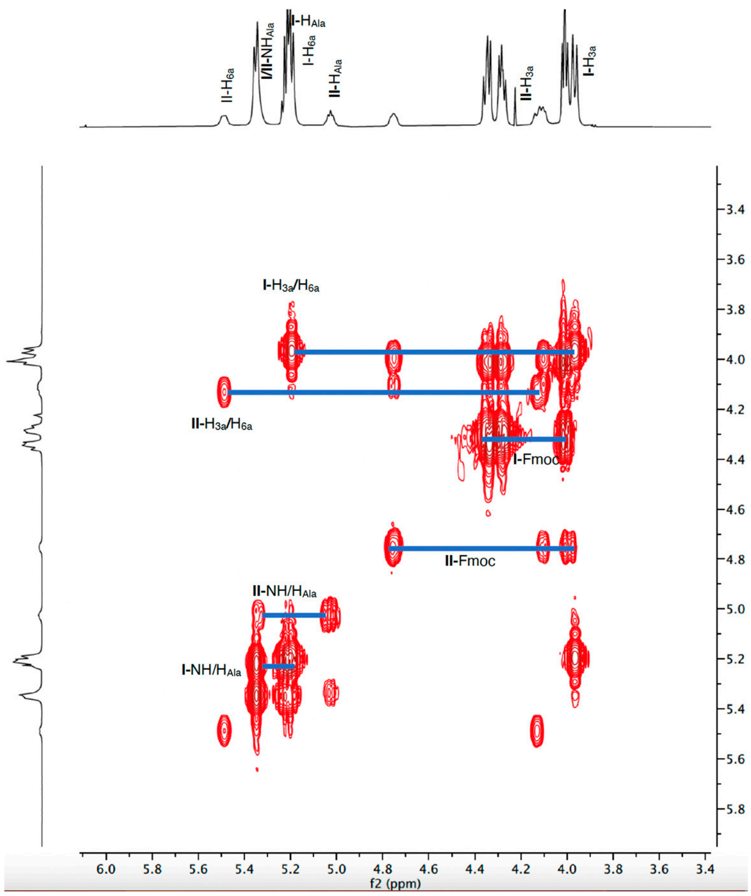

Figure S17. Cosy of compound **5’b** (1% C_6D_6, 400 MHz) at 283 K.

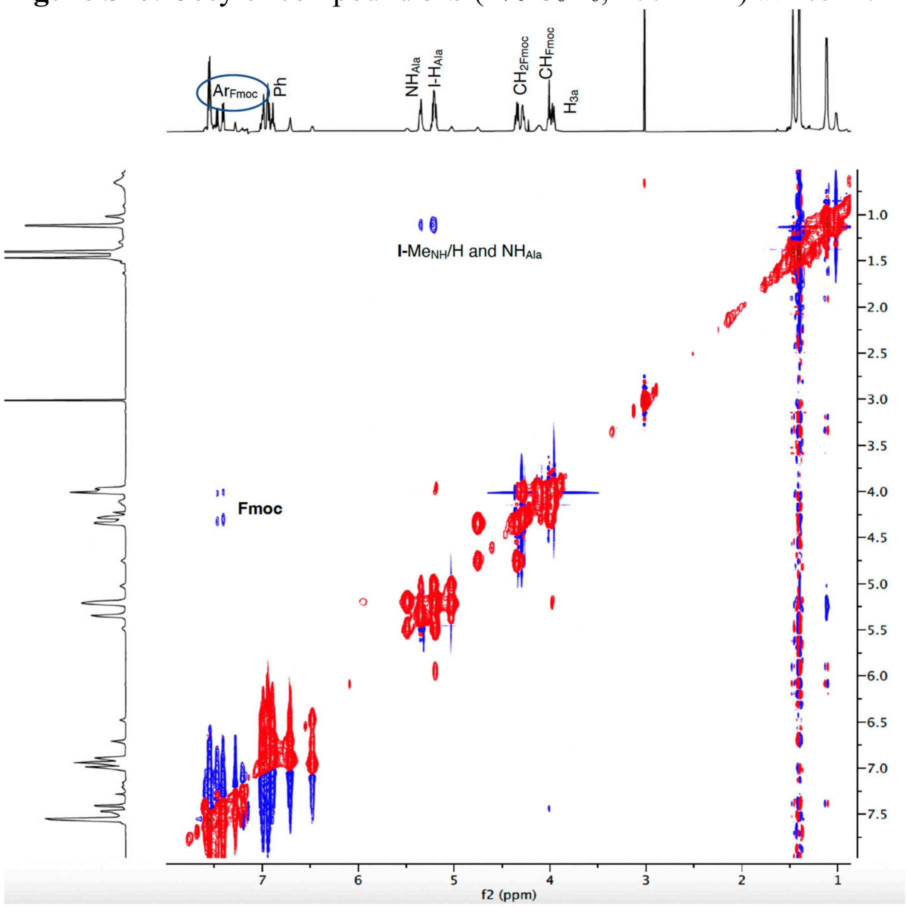

Figure S18. NOESY of compound **5’b** (1% C_6D_6, 500 MHz) at 500 ms and 283 K.

4. NMR SPECTRA OF COMPOUNDS 3, 4 AND 5/5’

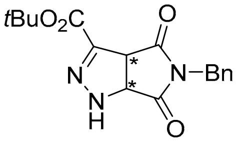

3c

^{1}H NMR ($CDCl_3$, 300 MHz, 300 K)

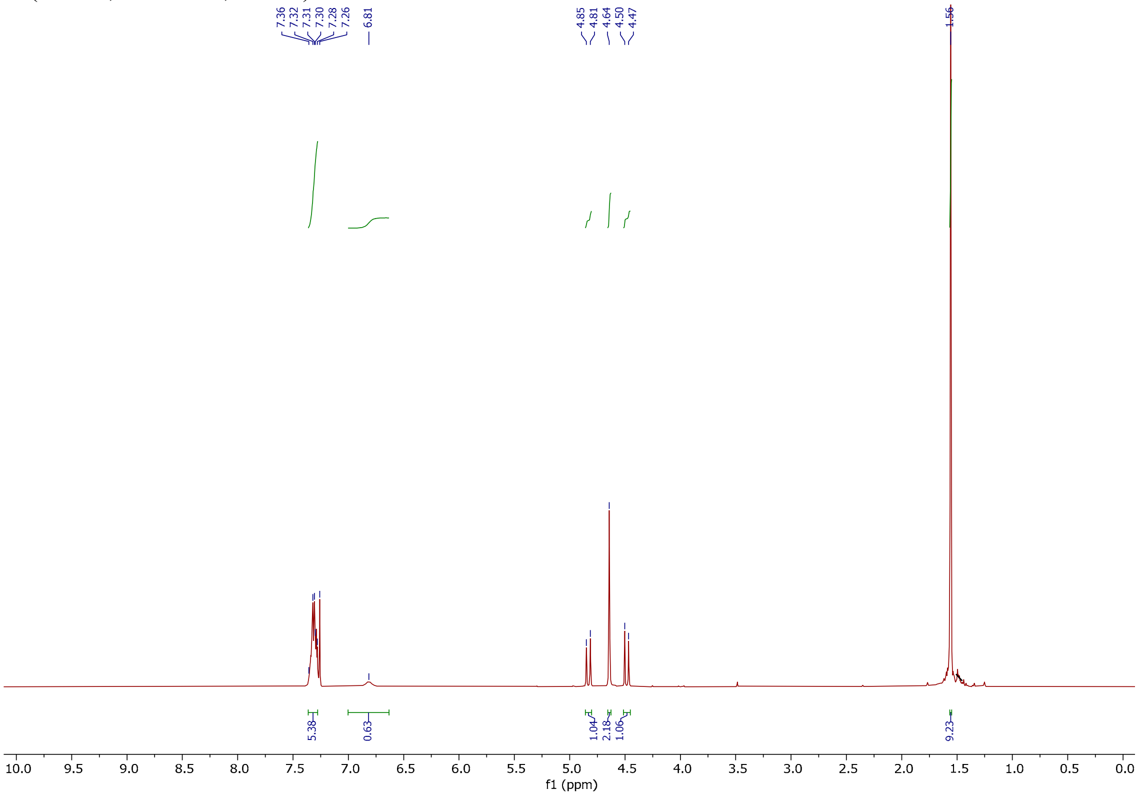

^{13}C NMR ($CDCl_3$, 75 MHz, 300 K)

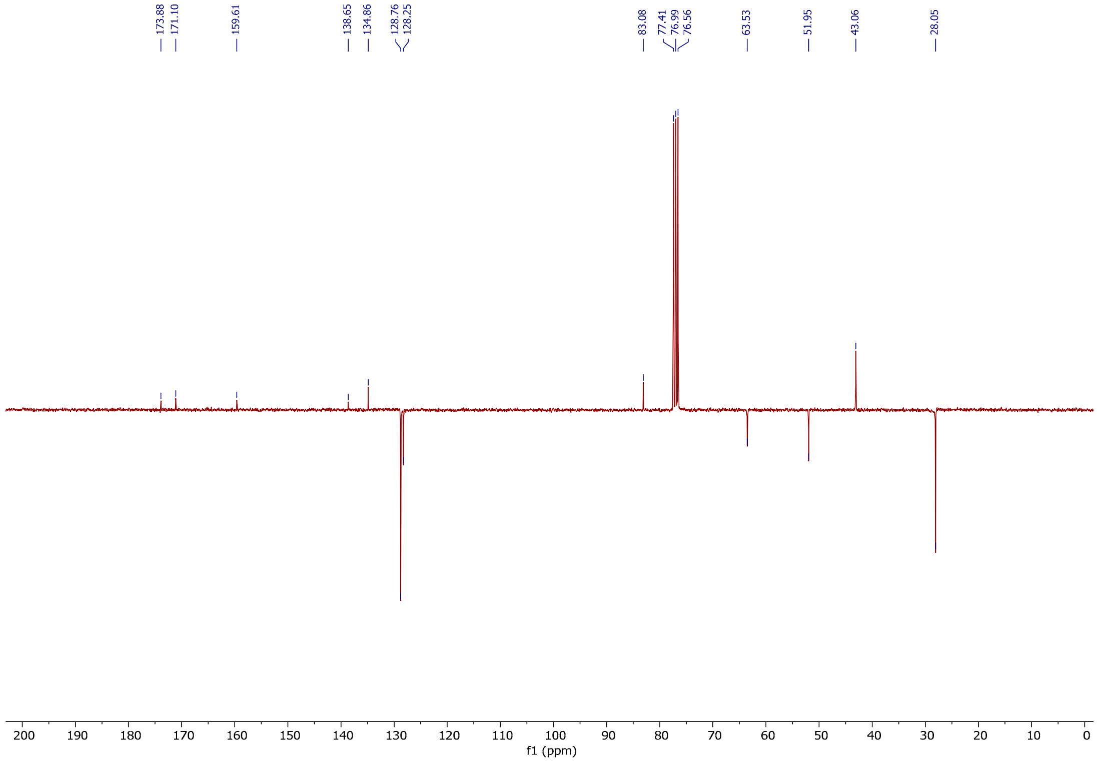

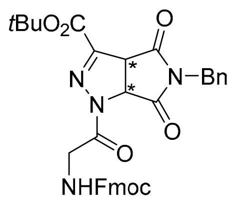

4c

^{1}H NMR (DMSO-d$_6$, 300 MHz, 300 K)

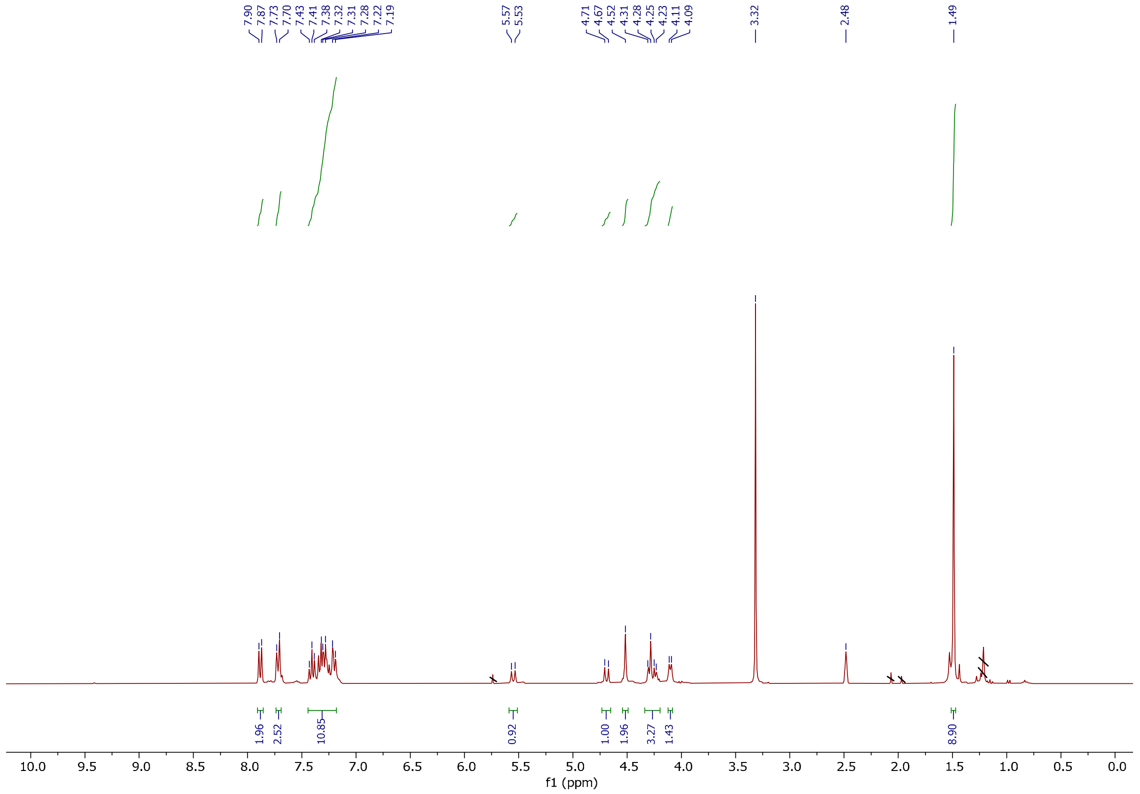

^{13}C NMR (DMSO-d$_6$, 75 MHz, 300 K)

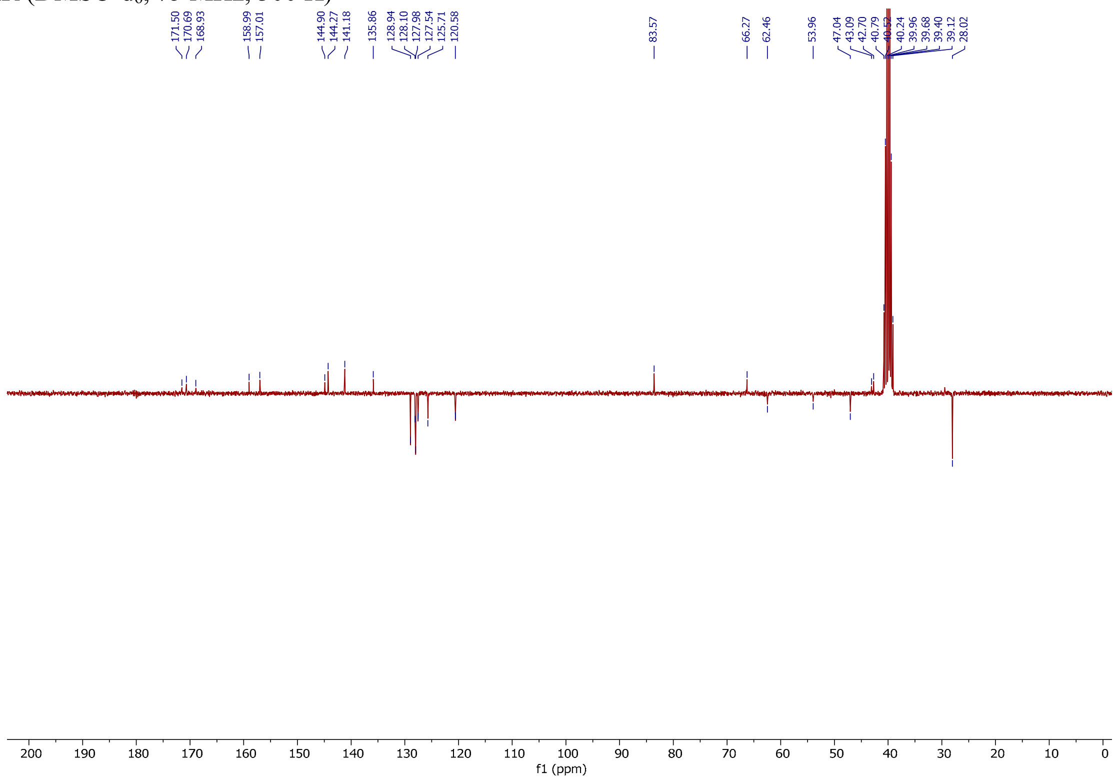

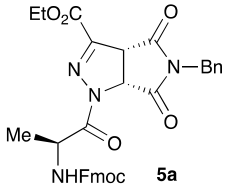

5a

^{1}H NMR (CDCl$_3$, 300 MHz, 300 K)

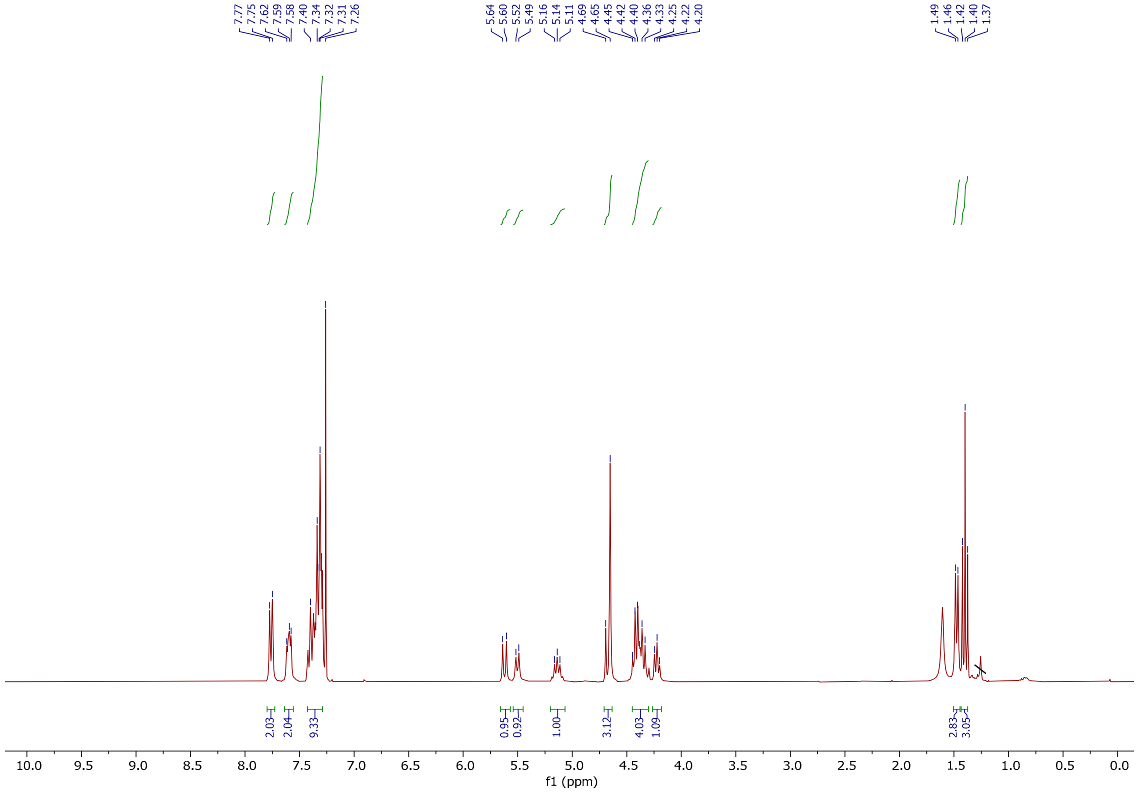

^{13}C NMR (CDCl$_3$, 100.7 MHz, 300 K)

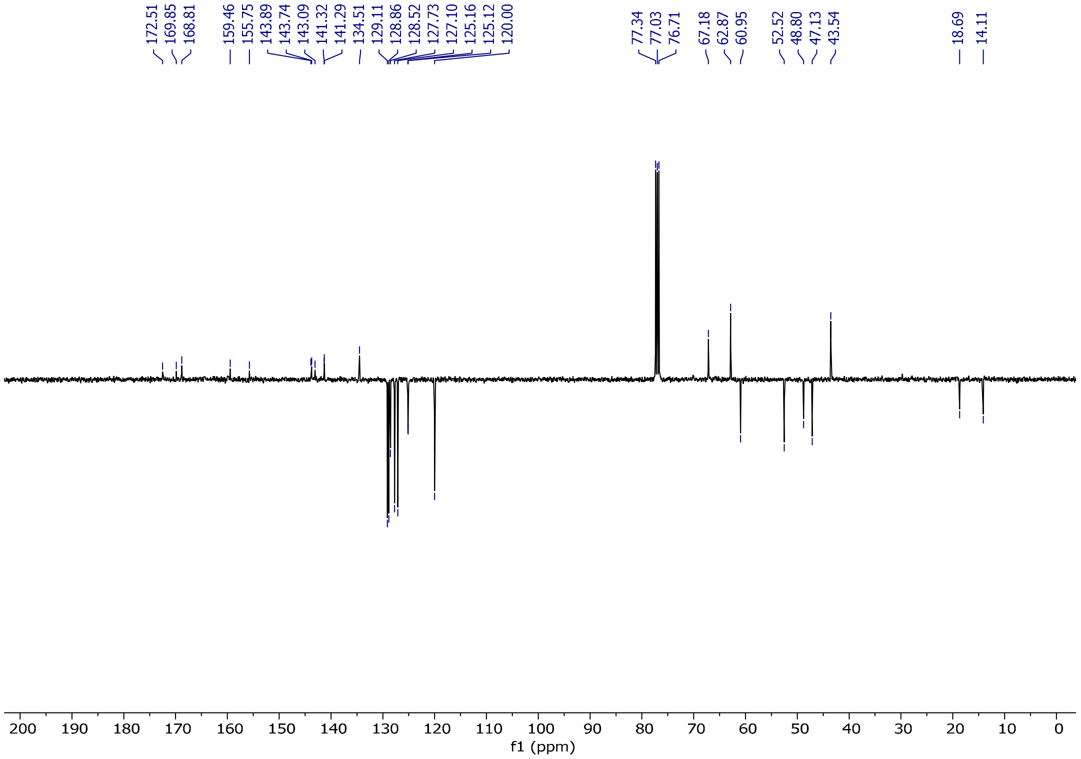

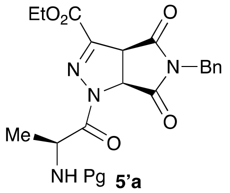

5’a

^{1}H NMR (CDCl$_3$, 300 MHz, 300 K)

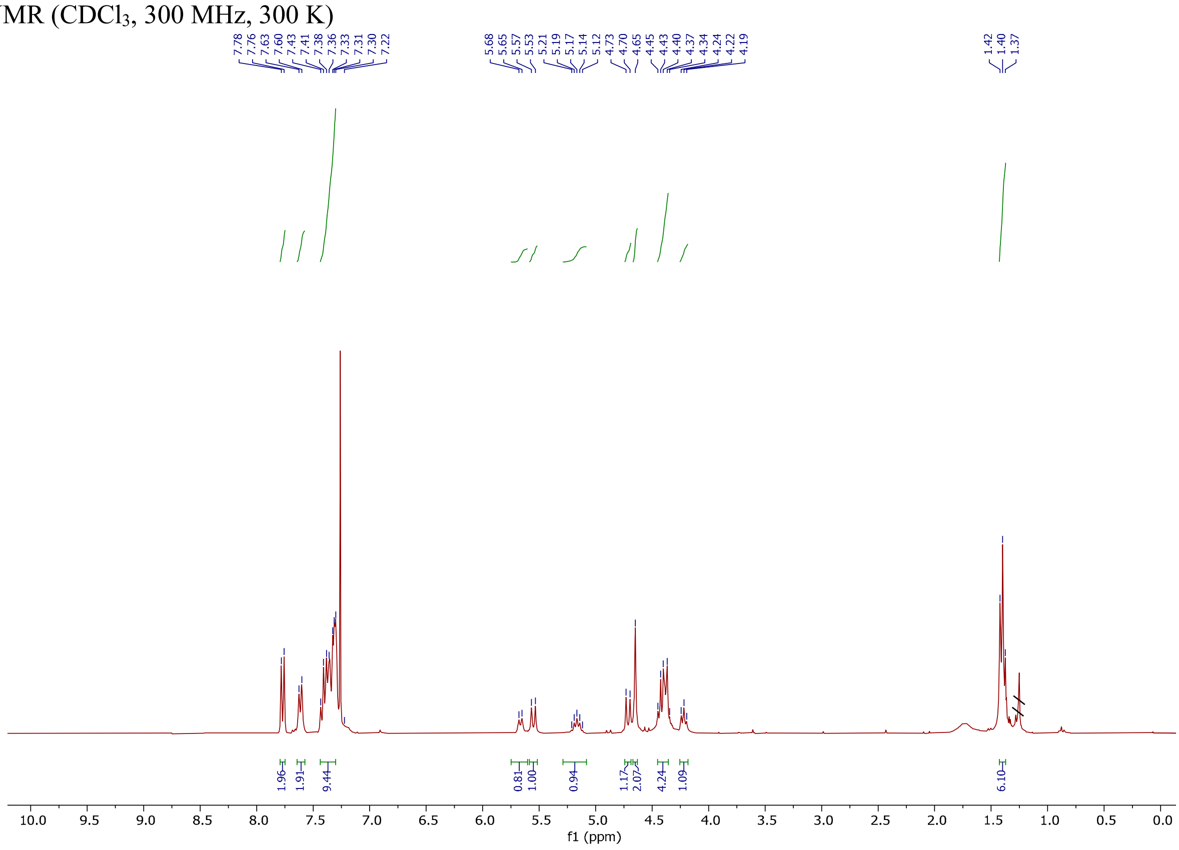

^{13}C NMR (CDCl$_3$, 75 MHz, 300 K)

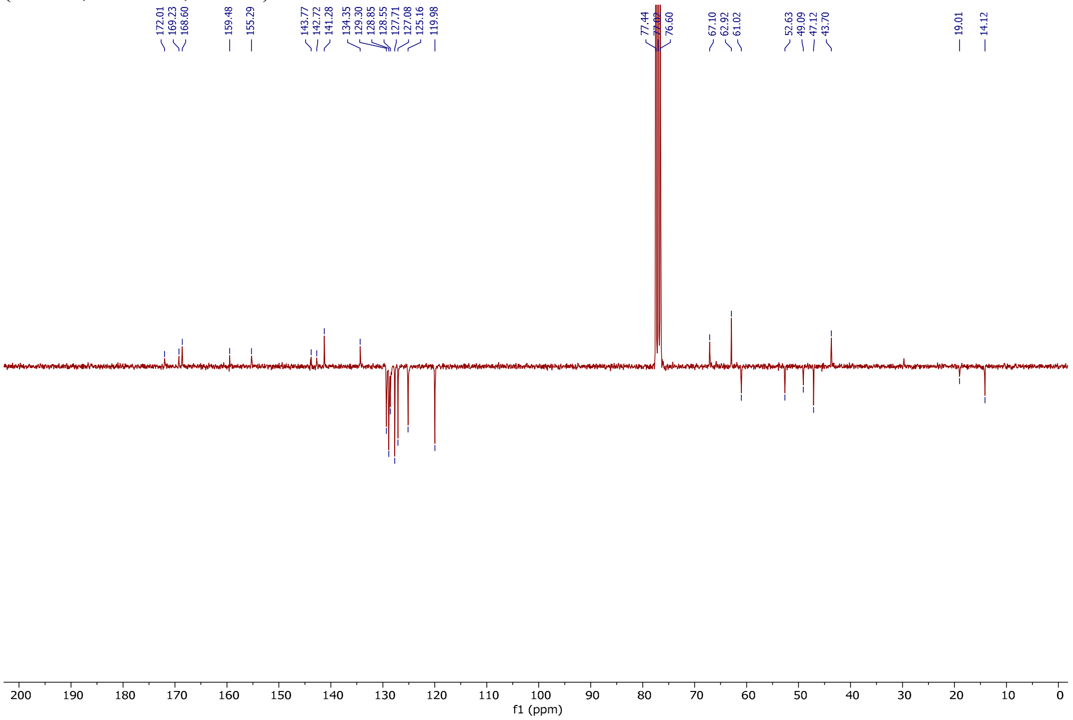

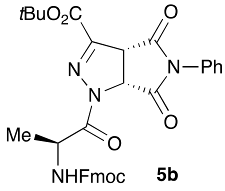

5b

^{1}H NMR ($CDCl_3$, 2% w/v, 300 MHz, 300 K)

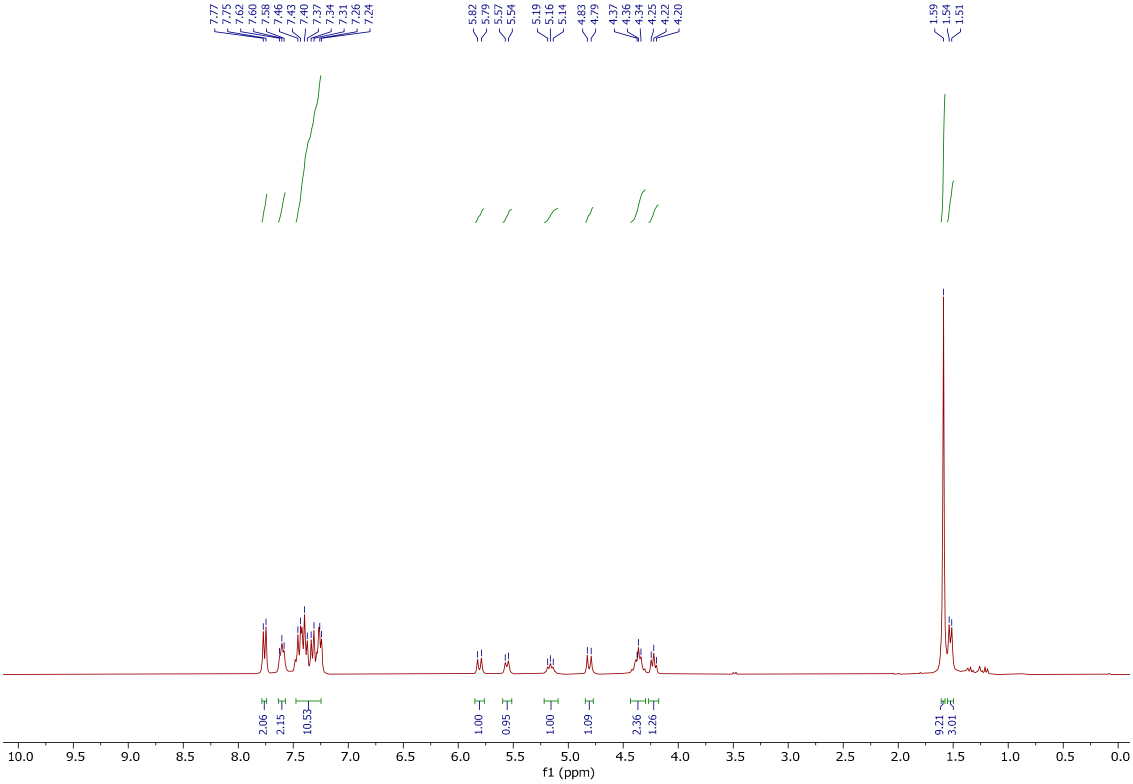

^{13}C NMR ($CDCl_3$, 2% w/v, 75 MHz, 300 K)

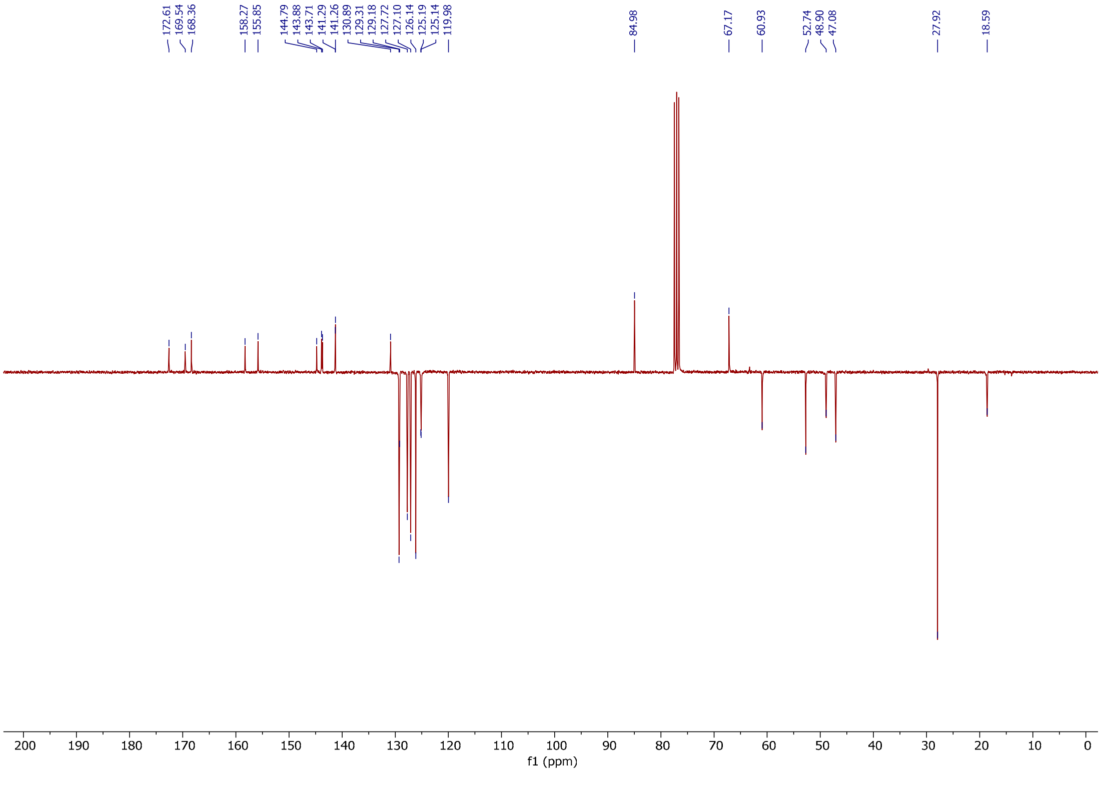

^{1}H NMR ($CDCl_3$, 1% w/v, 400 MHz, 300 K)

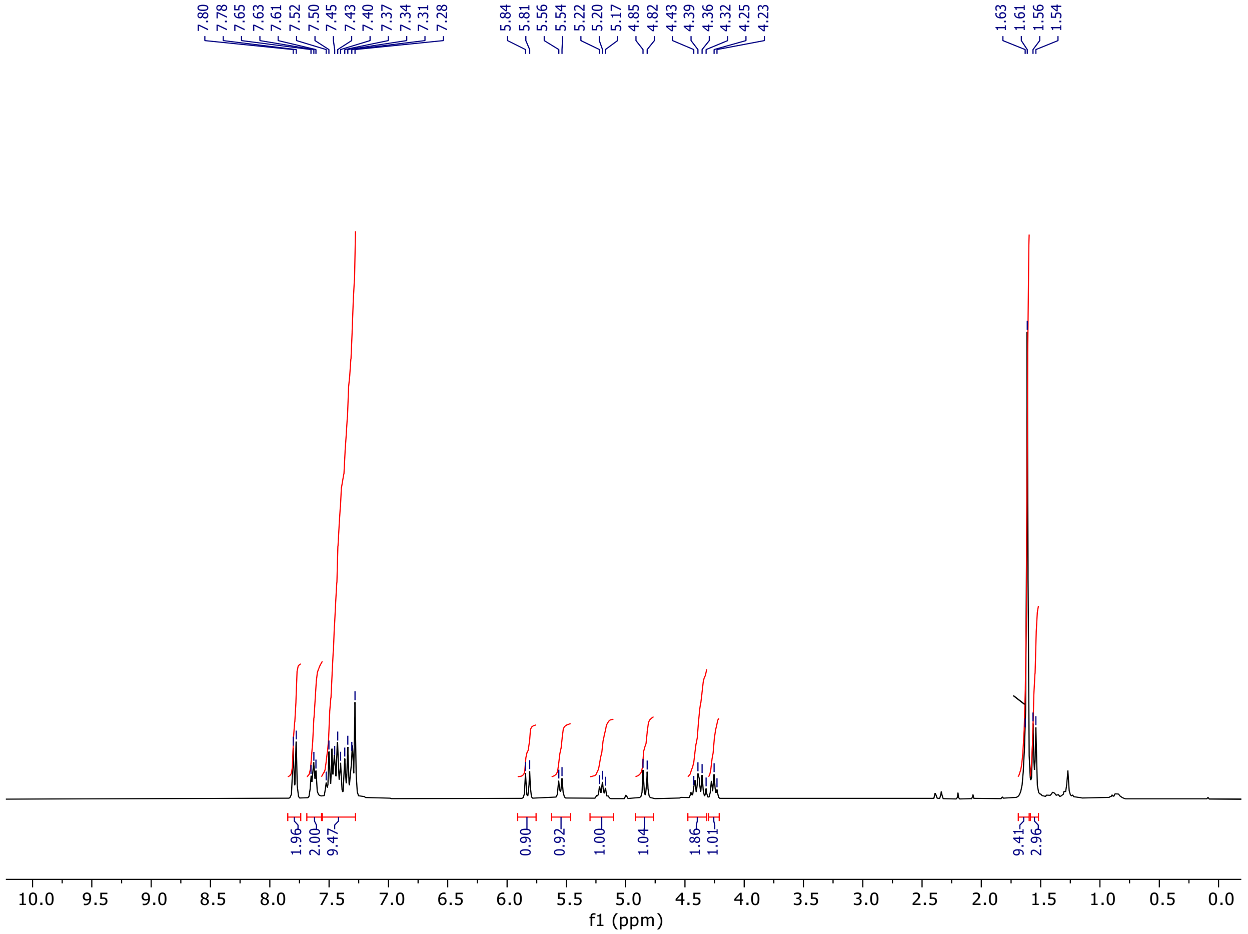

COSY NMR ($CDCl_3$, 1% w/v, 400 MHz, 300 K)

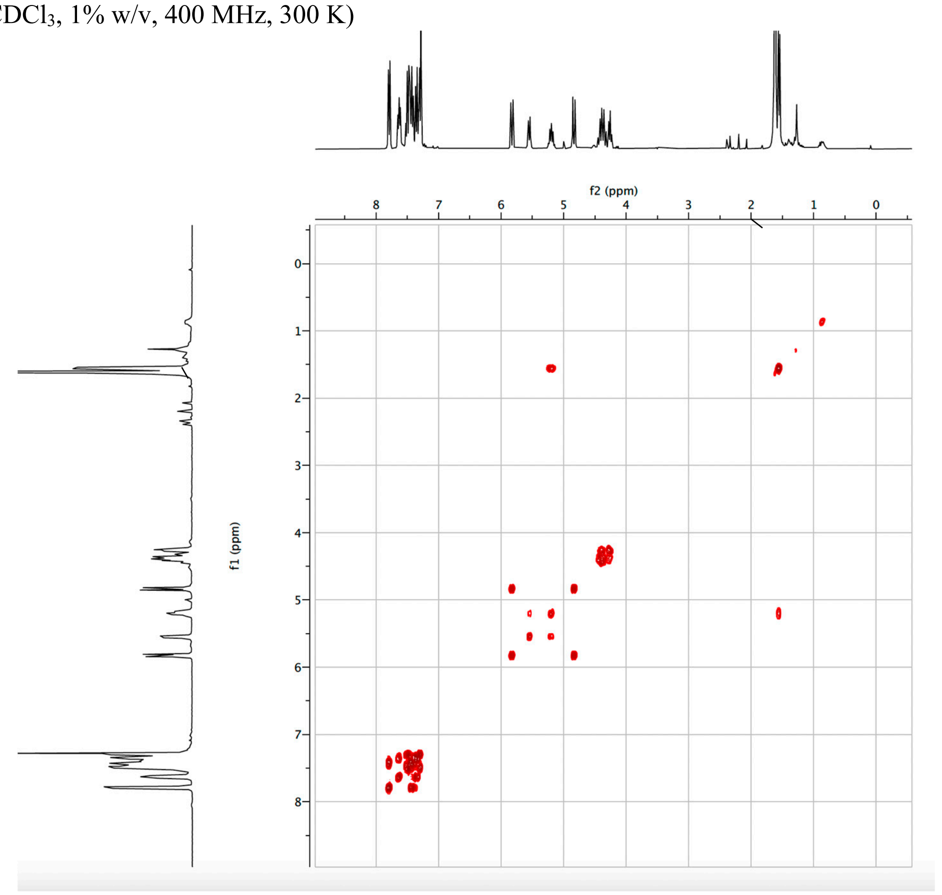

NOESY-NMR ($CDCl_3$, 1% w/v, 400 MHz, 300 K, t_{mix} = 800 ms)

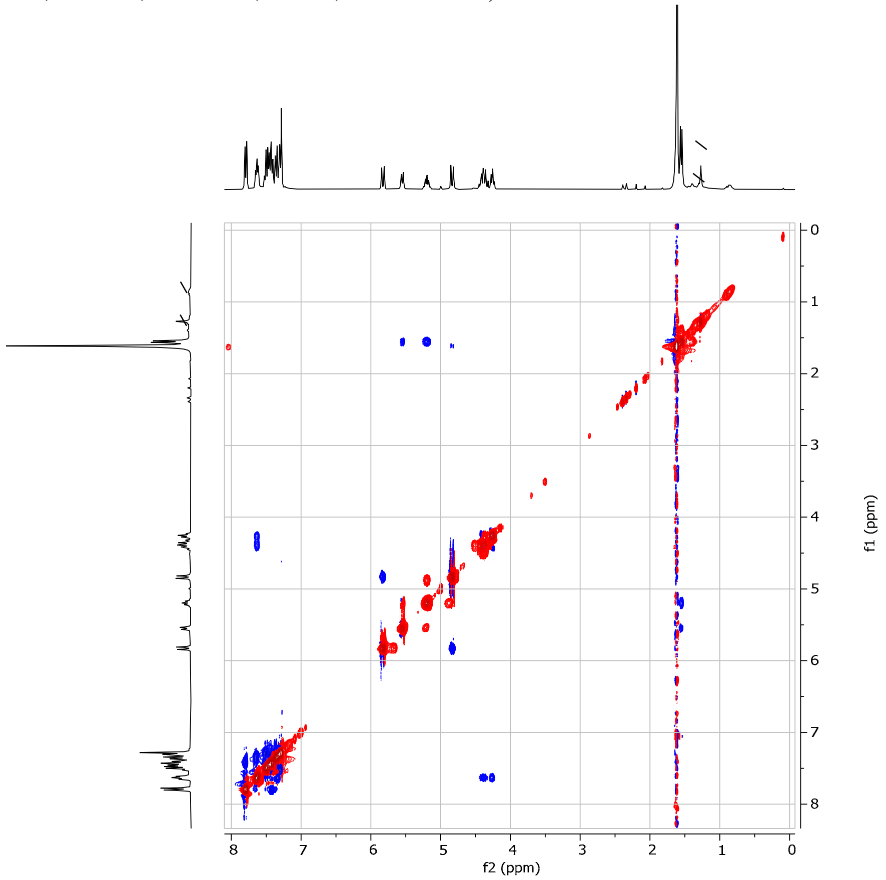

NOESY-NMR ($CDCl_3$, 1% w/v, 400 MHz, 300 K, t_{mix} = 1 s)

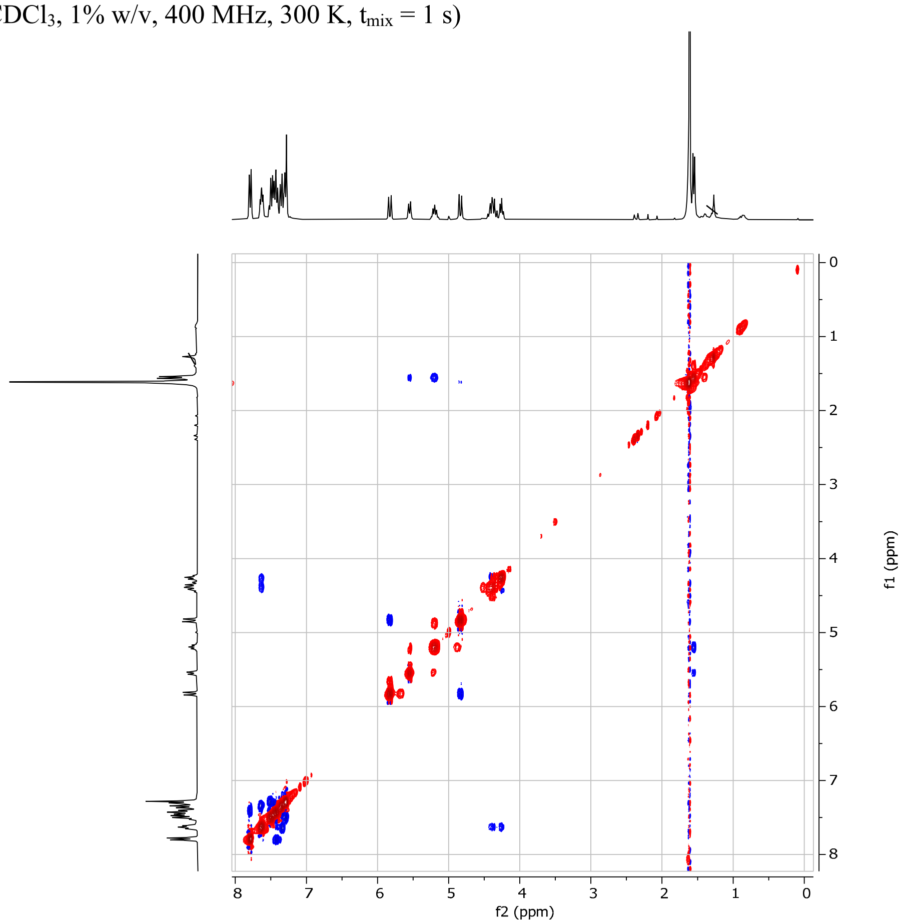

^{1}H NMR (C_6D_6, 1% w/v, 400 MHz, 300 K)

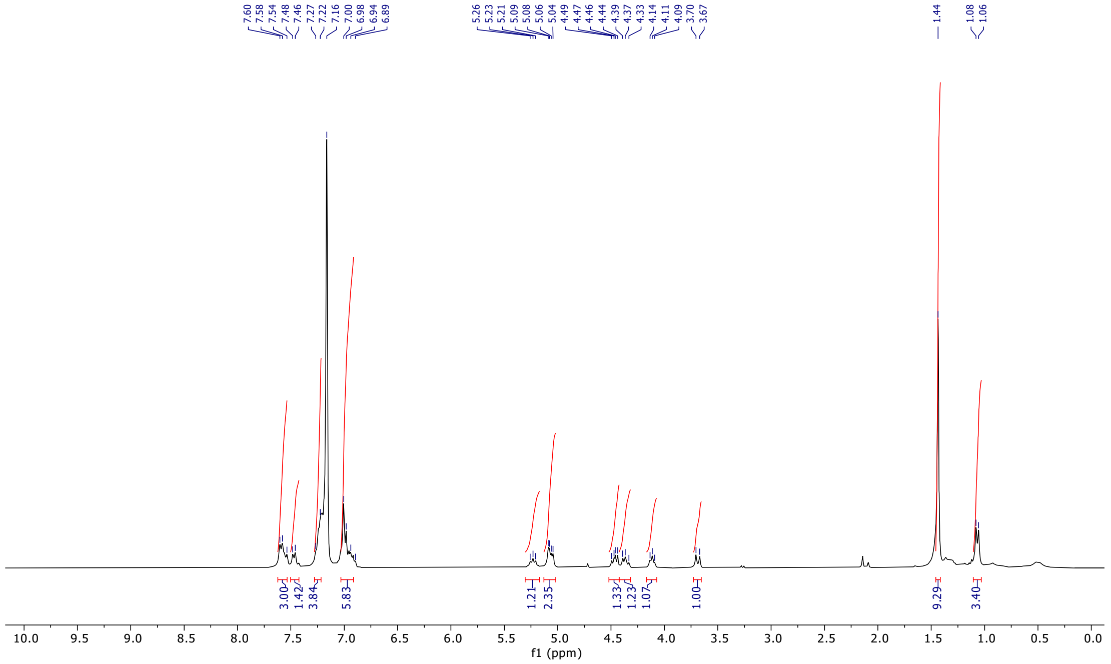

COSY-NMR (C_6D_6, 1% w/v, 400 MHz, 300 K)

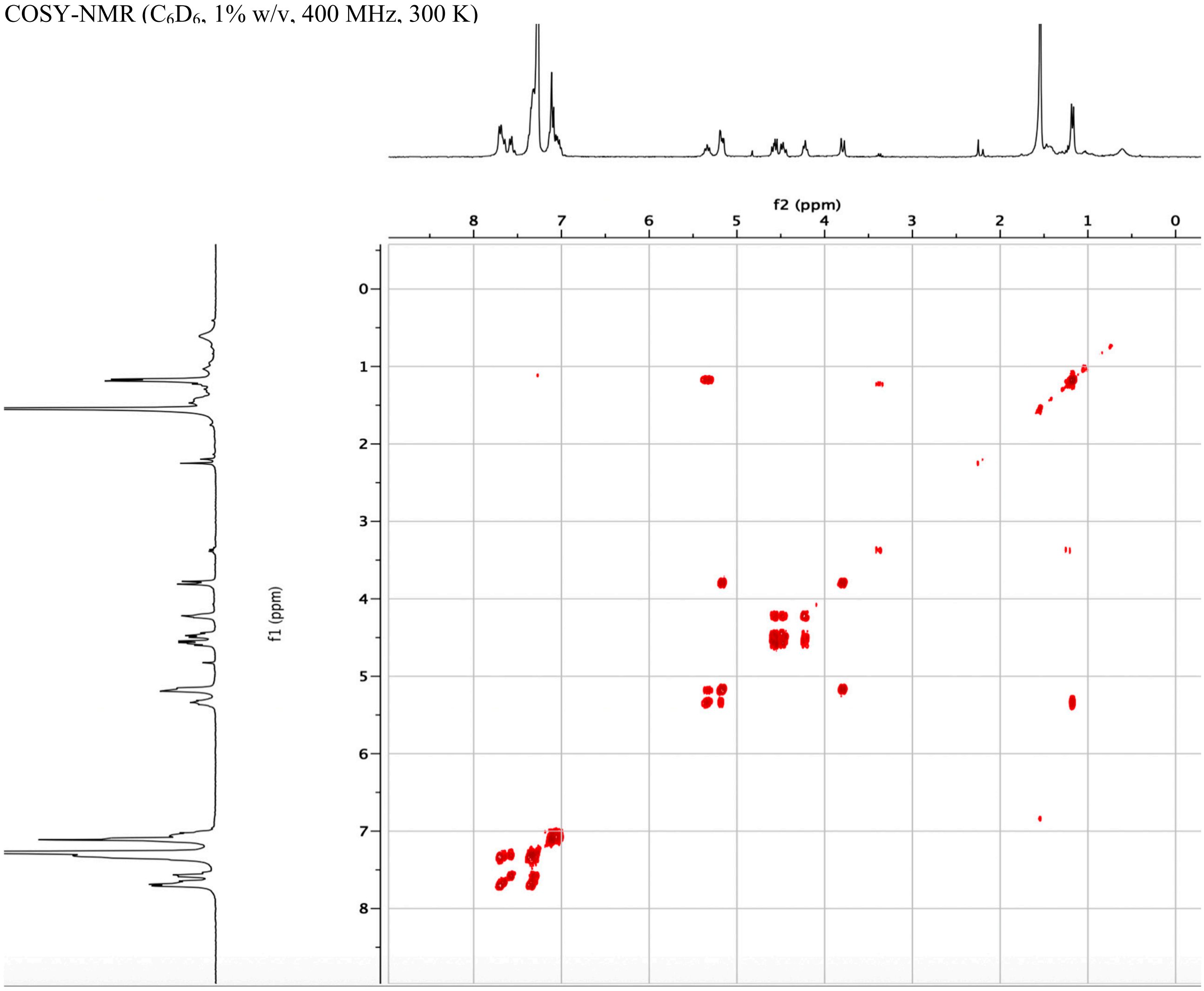

^1H NMR (toluene-d_8/dioxane-d_8 (10:1), 1% w/v, 400 MHz, 300 K)

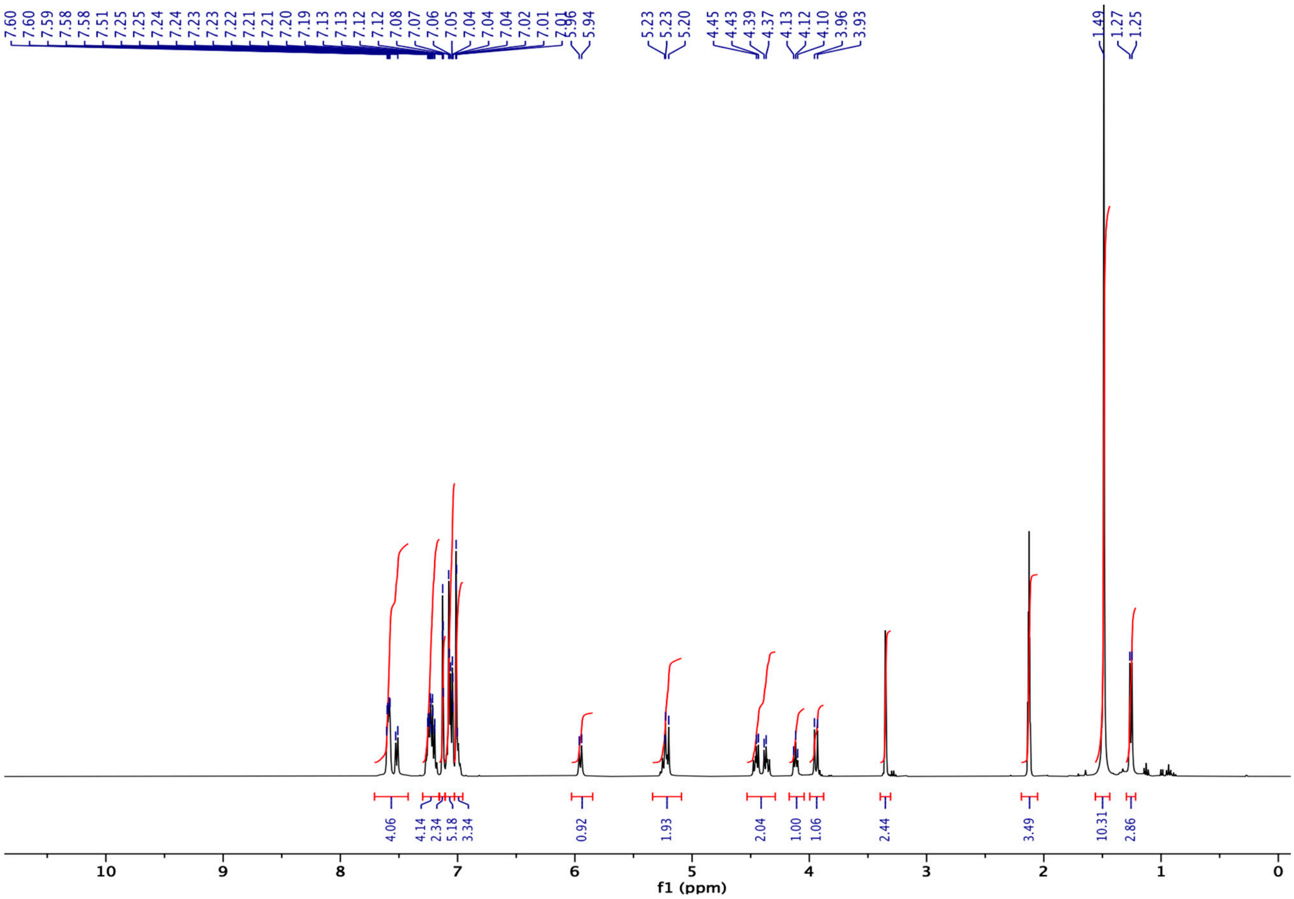

COSY-NMR (toluene-d_8/dioxane-d_8 (10:1), 1% w/v, 400 MHz, 300 K)

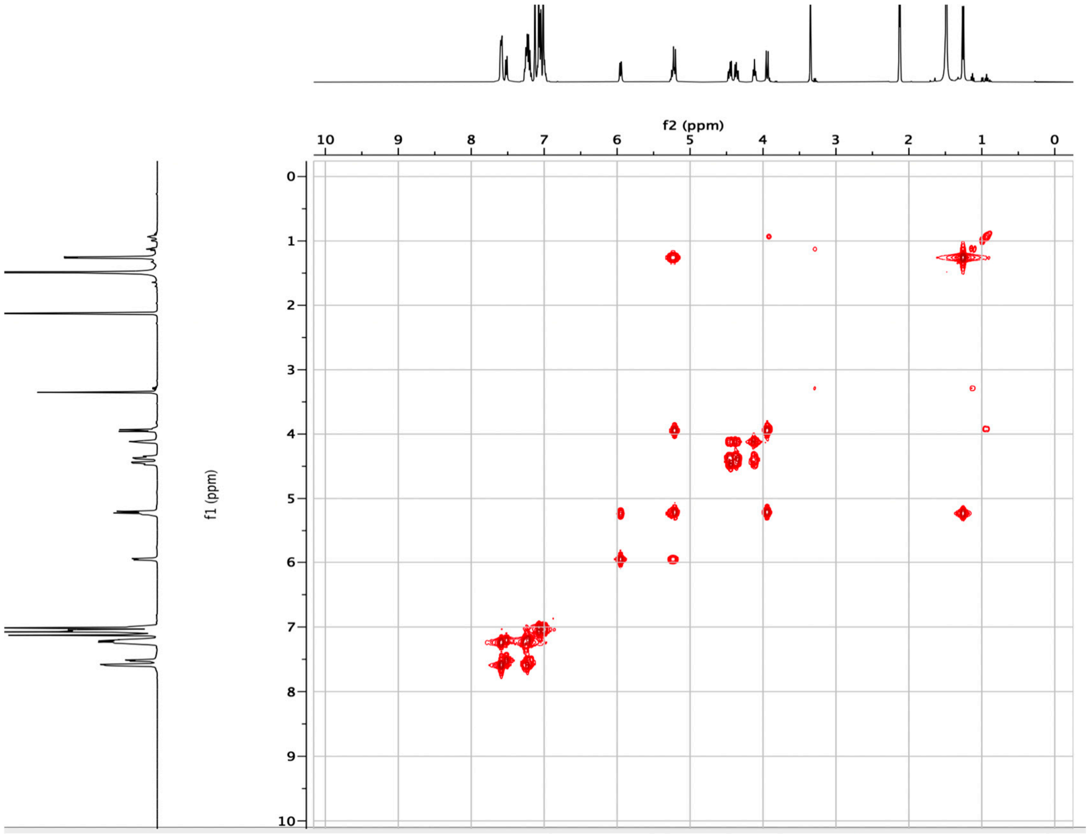

^{13}C NMR (toluene-d$_8$/dioxane-d$_8$ (10:1), 1% w/v, 100.7 MHz, 300 K)

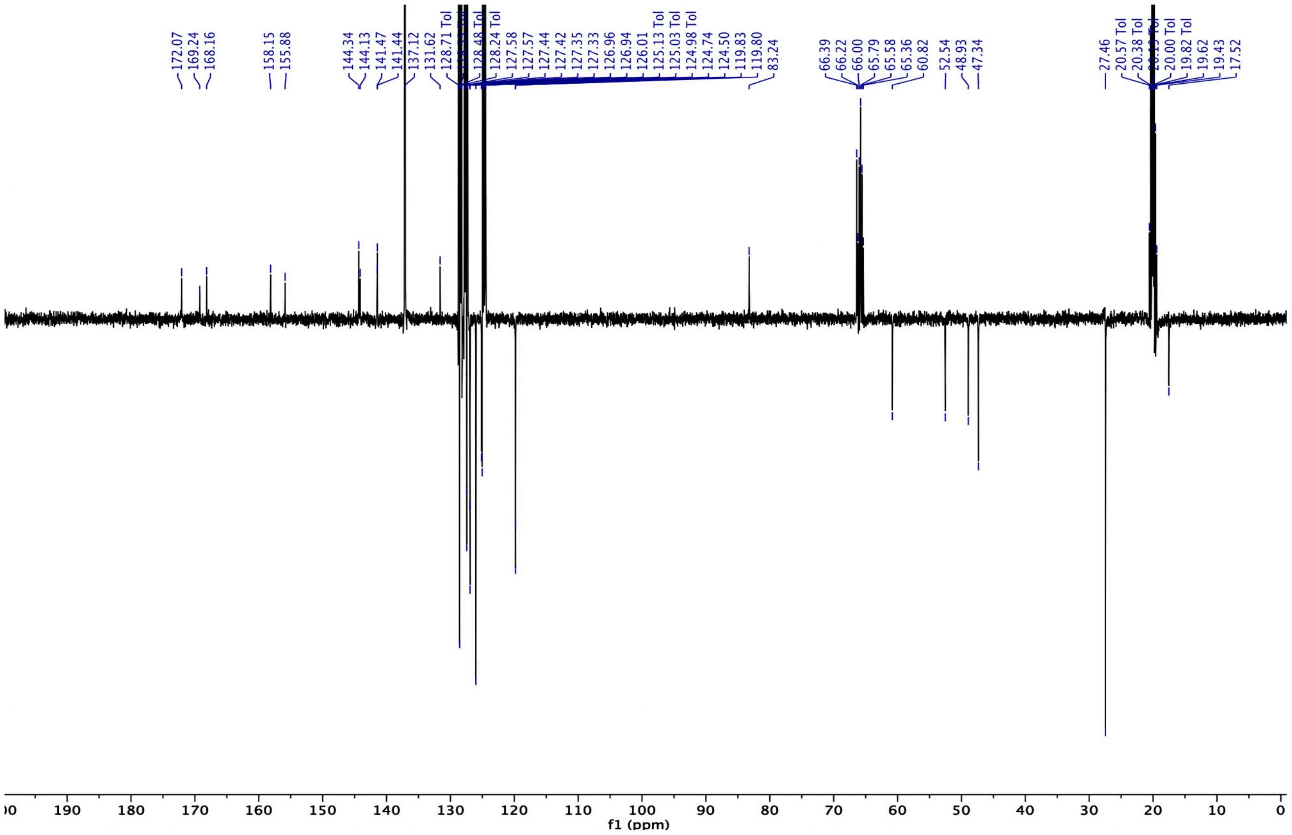

HSQC-NMR (toluene-d$_8$/dioxane-d$_8$ (10:1), 1% w/v, 400 MHz, 100.7 MHz, 300 K)

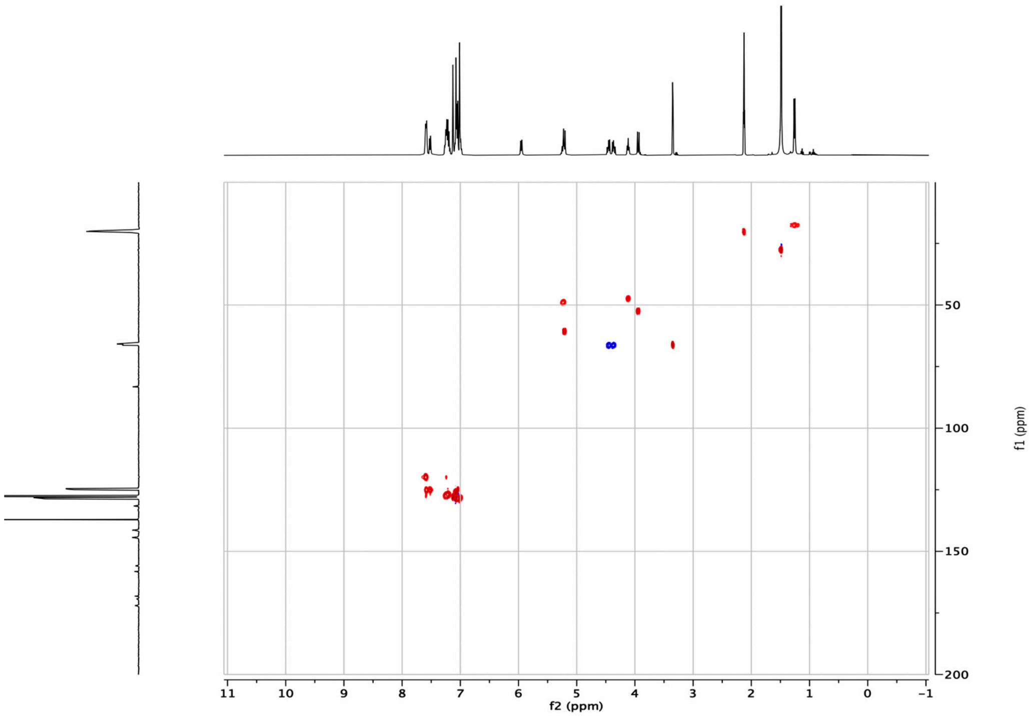

NOESY-NMR (toluene-d_8/dioxane-d_8 (10:1), 1% w/v, 400 MHz, 300 K, t_{mix} = 300 ms)

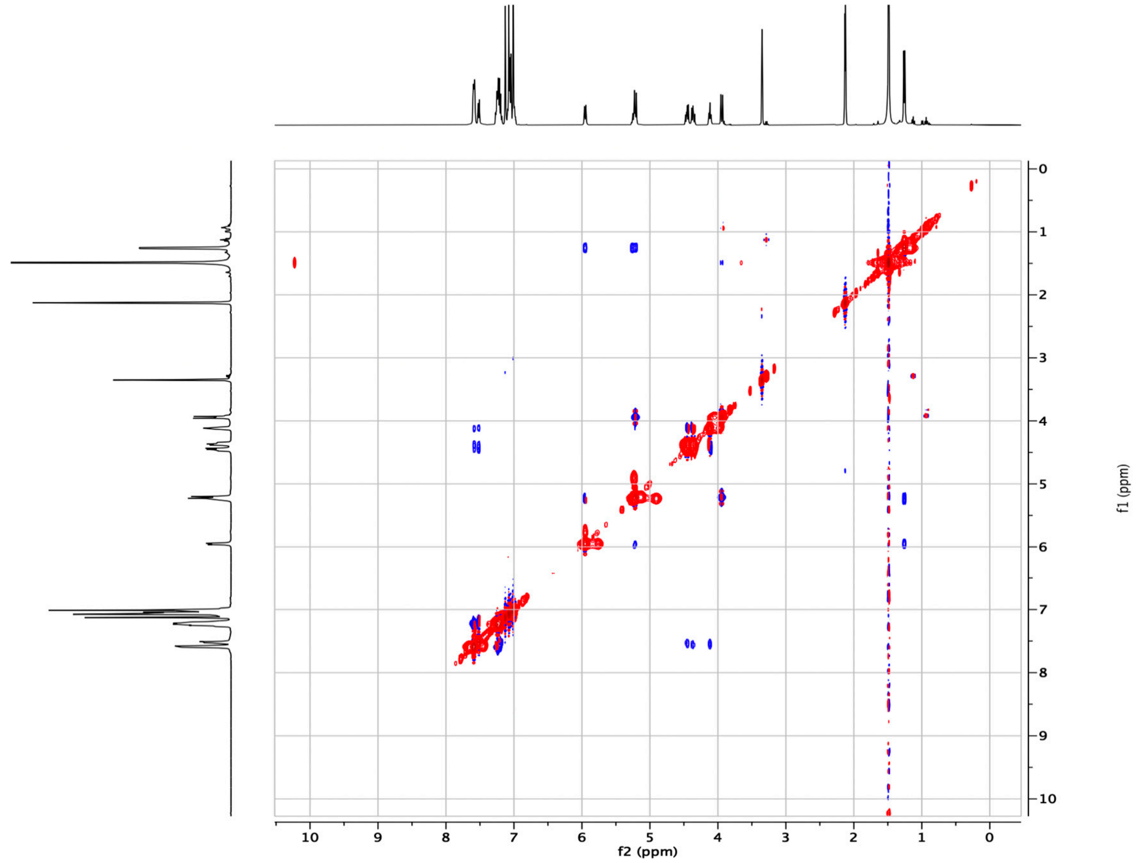

NOESY-NMR (toluene-d_8/dioxane-d_8 (10:1), 1% w/v, 400 MHz, 300 K, t_{mix} = 600 ms)

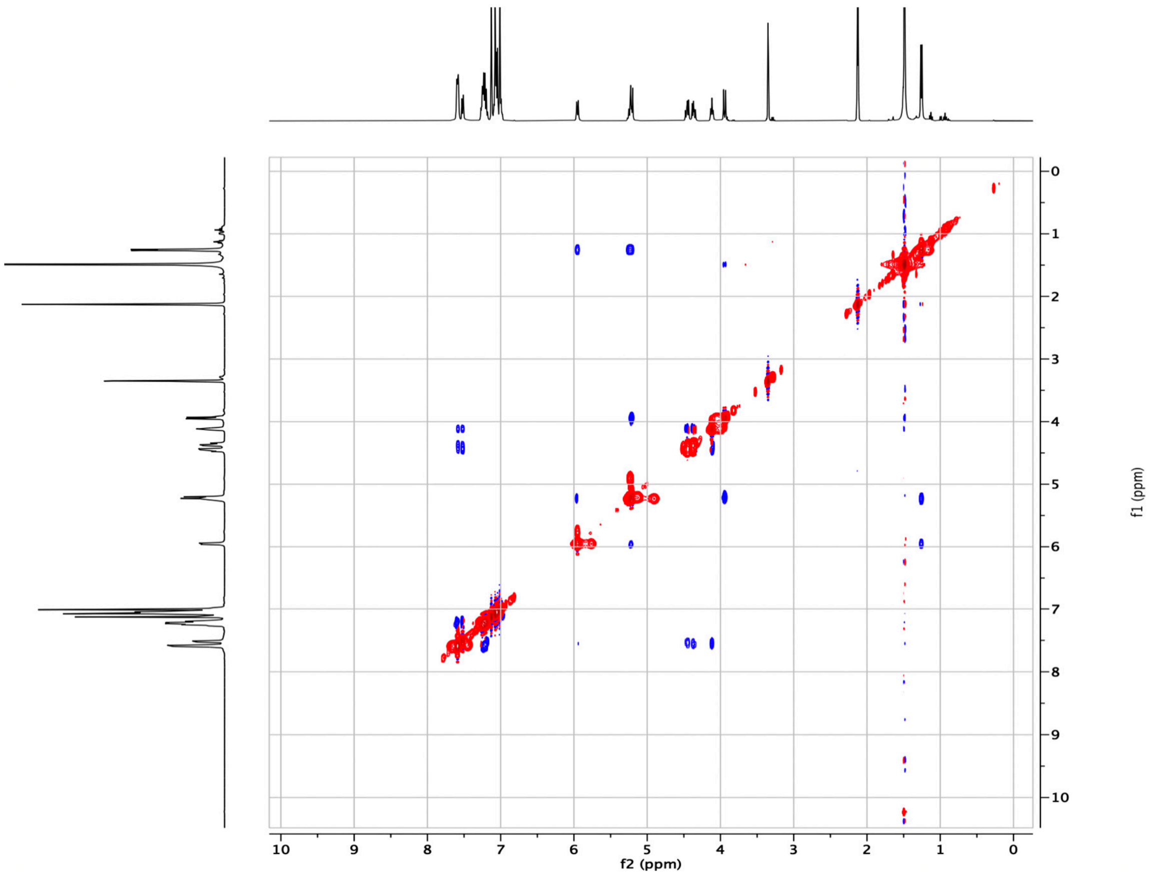

^{1}H NMR ($CDCl_3/C_6D_6$, 1:1, 1% w/v, 400 MHz, 300 K)

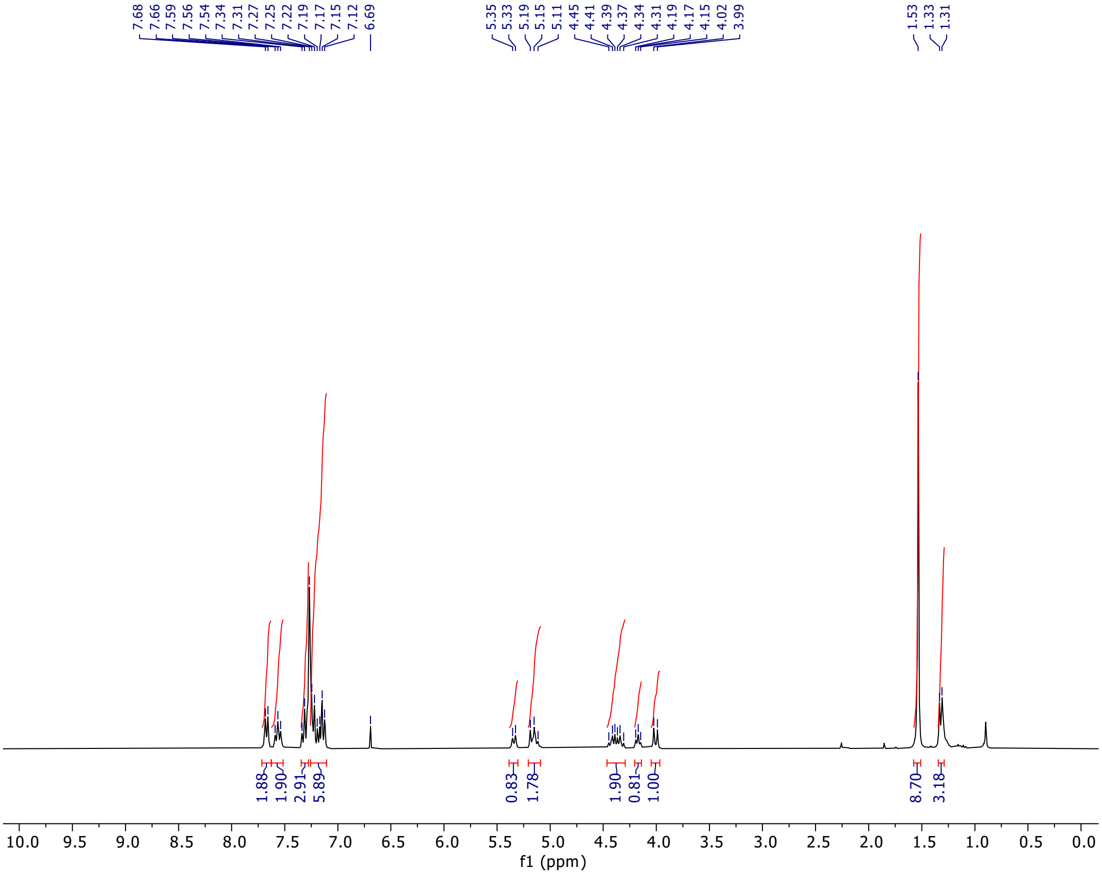

COSY-NMR ($CDCl_3/C_6D_6$, 1:1, 1% w/v, 400 MHz, 300 K)

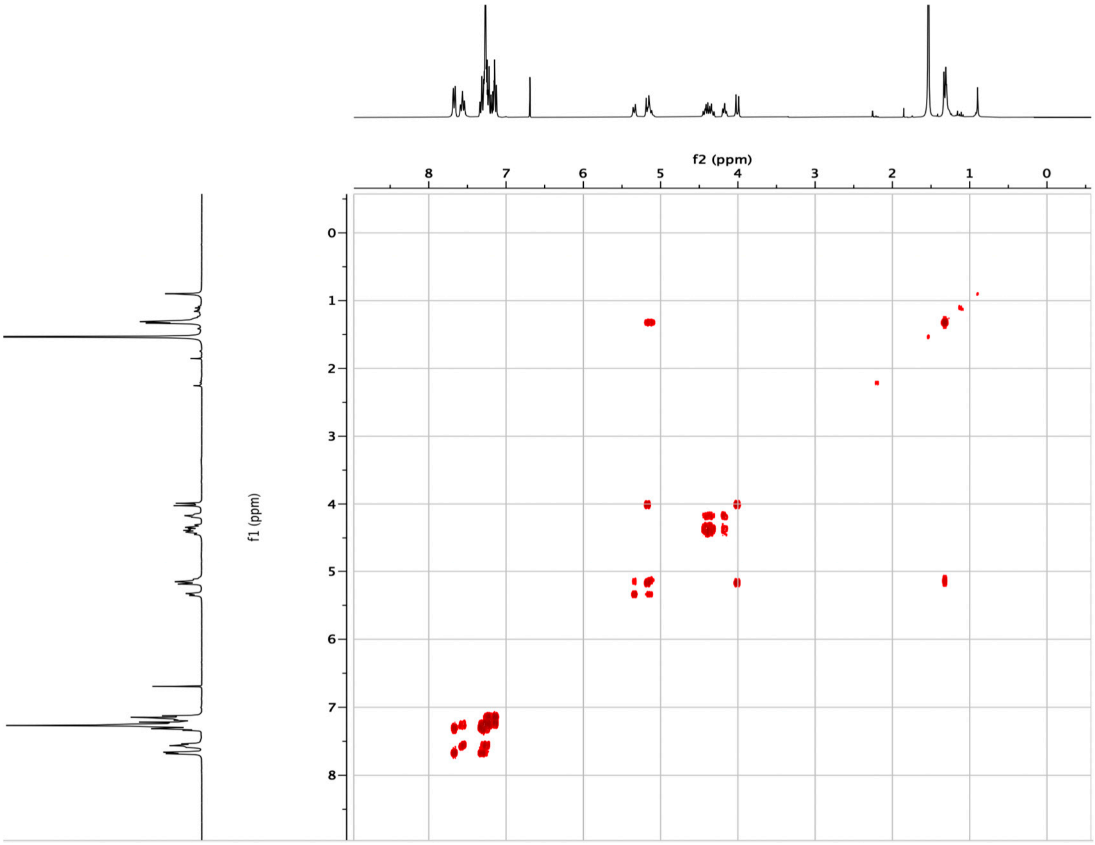

NOESY-NMR ($CDCl_3$/C_6D_6, 1:1, 1% w/v, 400 MHz, 300 K, t_{mix} = 400 ms)

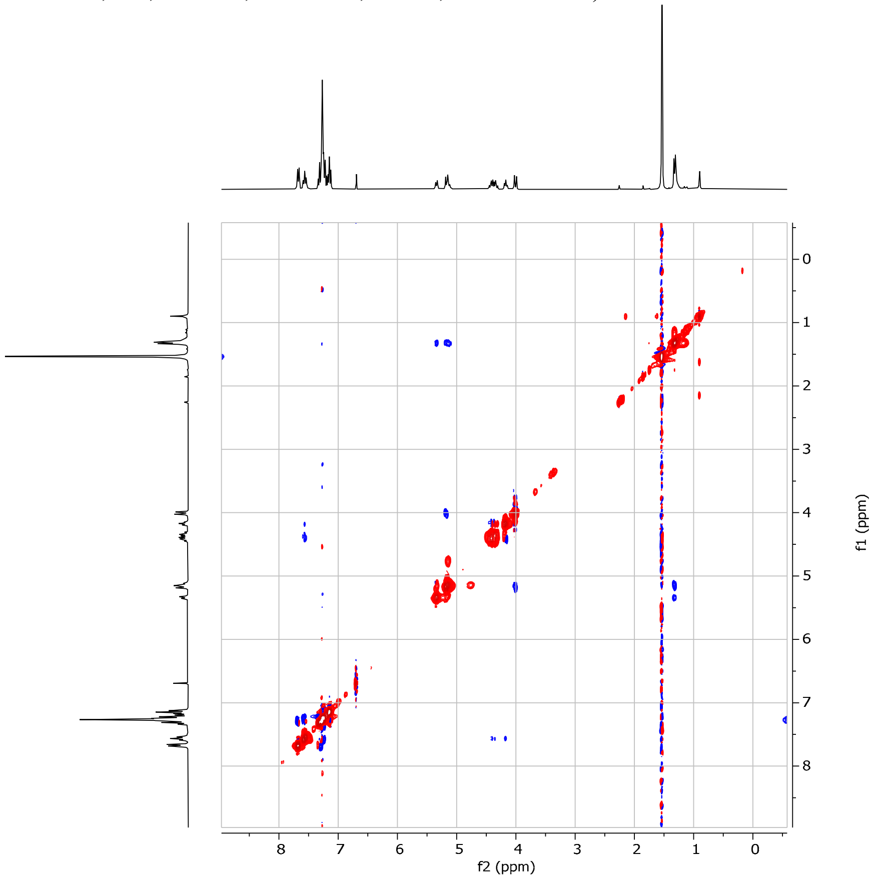

NOESY-NMR ($CDCl_3$/C_6D_6, 1:1, 1% w/v, 400 MHz, 300 K, t_{mix} = 600 ms)

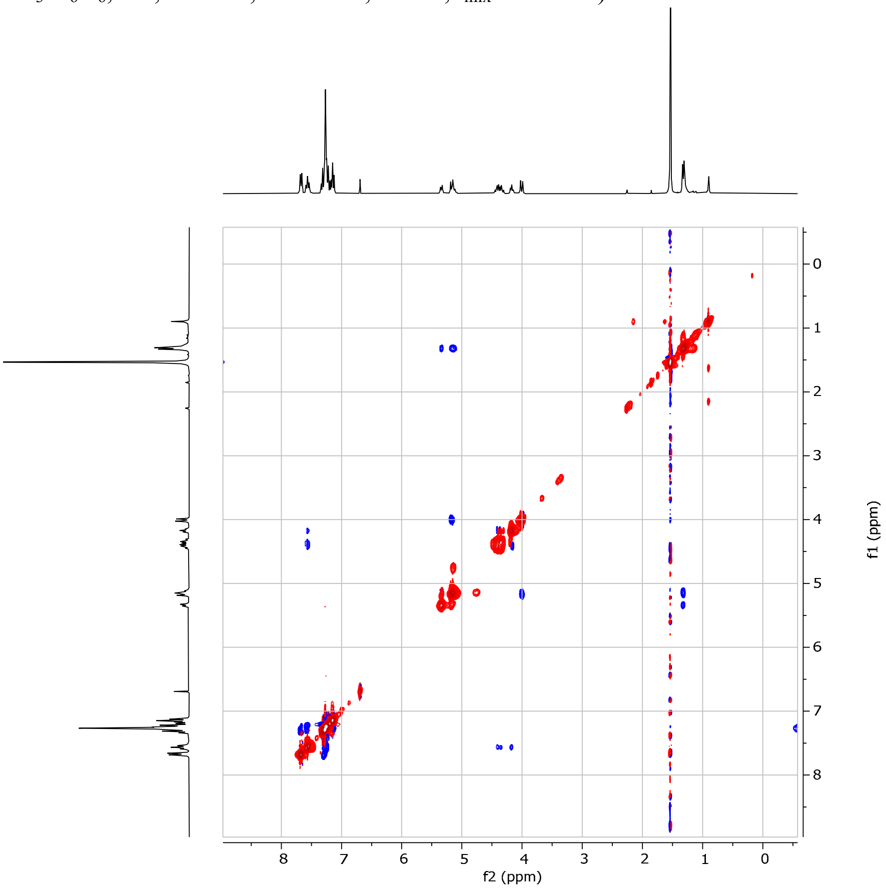

NOESY-NMR ($CDCl_3/C_6D_6$, 1:1, 1% w/v, 400 MHz, 300 K, t_{mix} = 1 s)

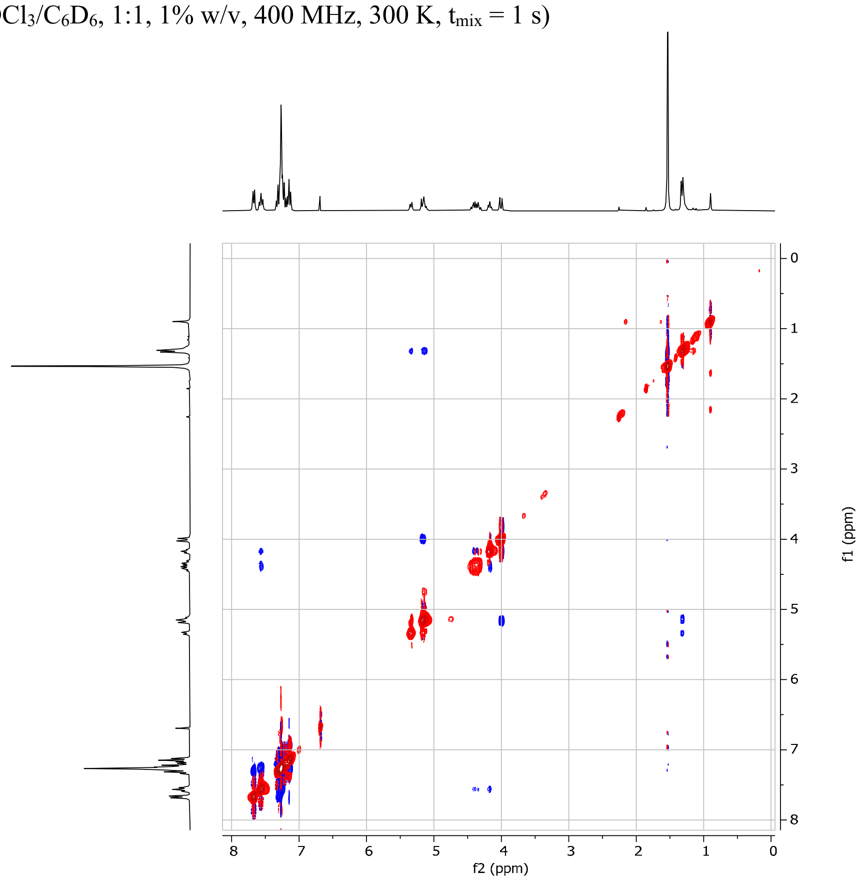

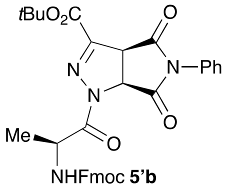

^{1}H NMR (CDCl$_3$, 1% w/v, 400 MHz, 300 K)

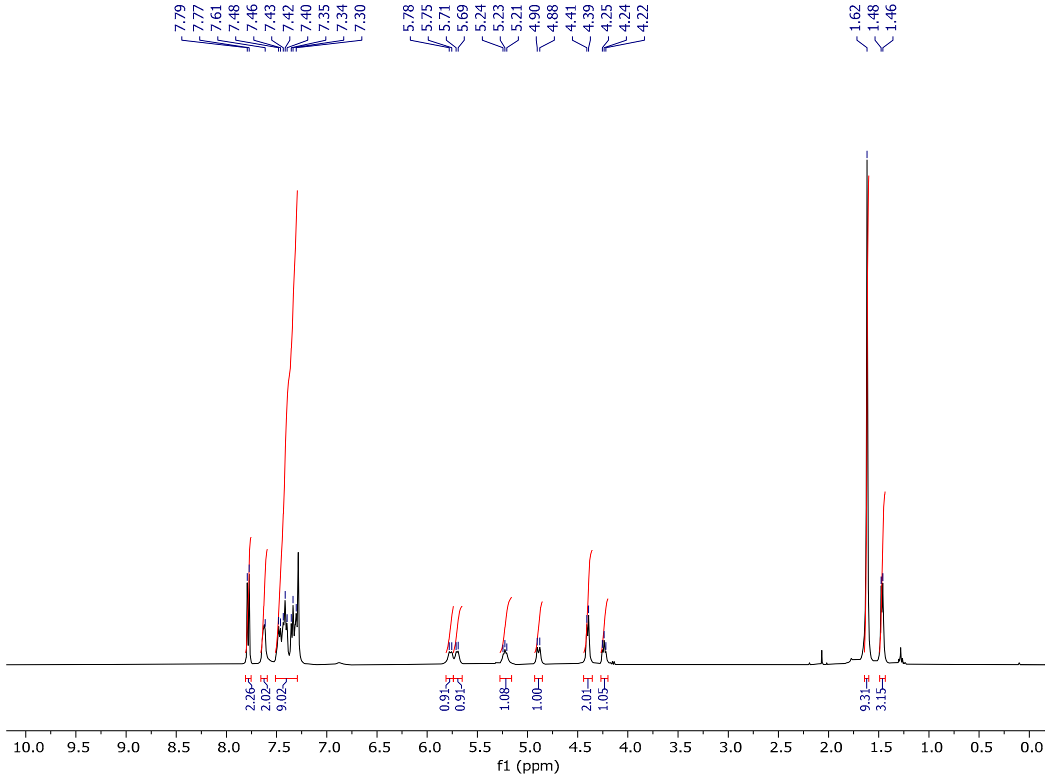

^{13}C NMR (CDCl$_3$, 1% w/v, 100.7 MHz, 300 K)

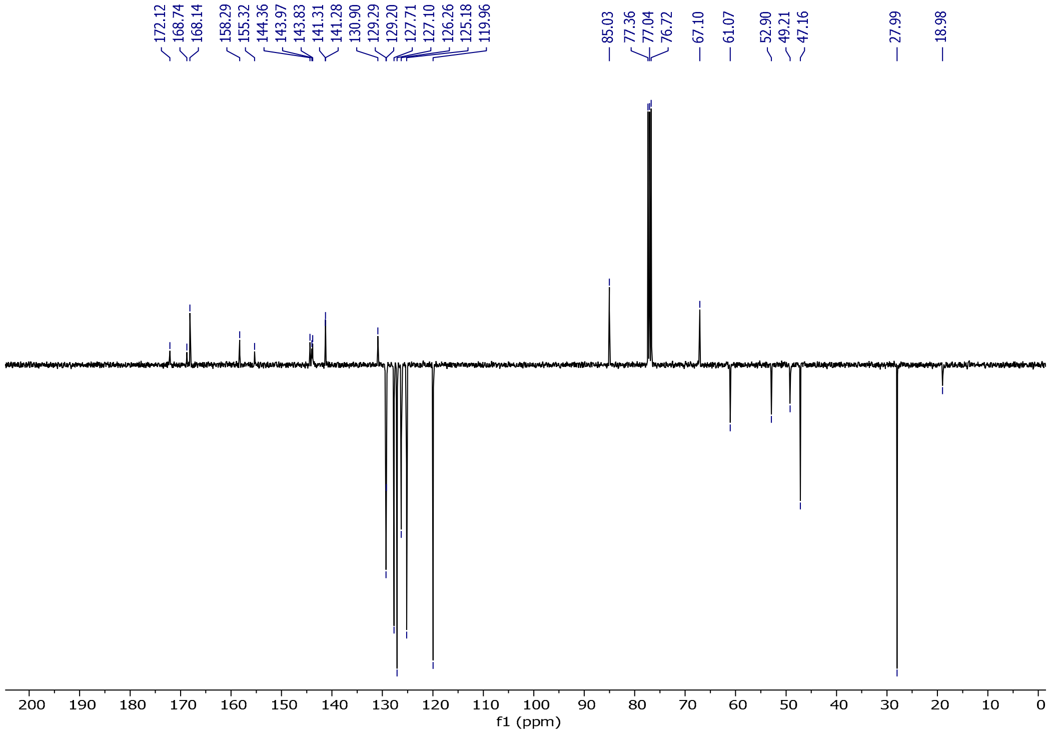

COSY-NMR ($CDCl_3$, 1% w/v, 400 MHz, 300 K)

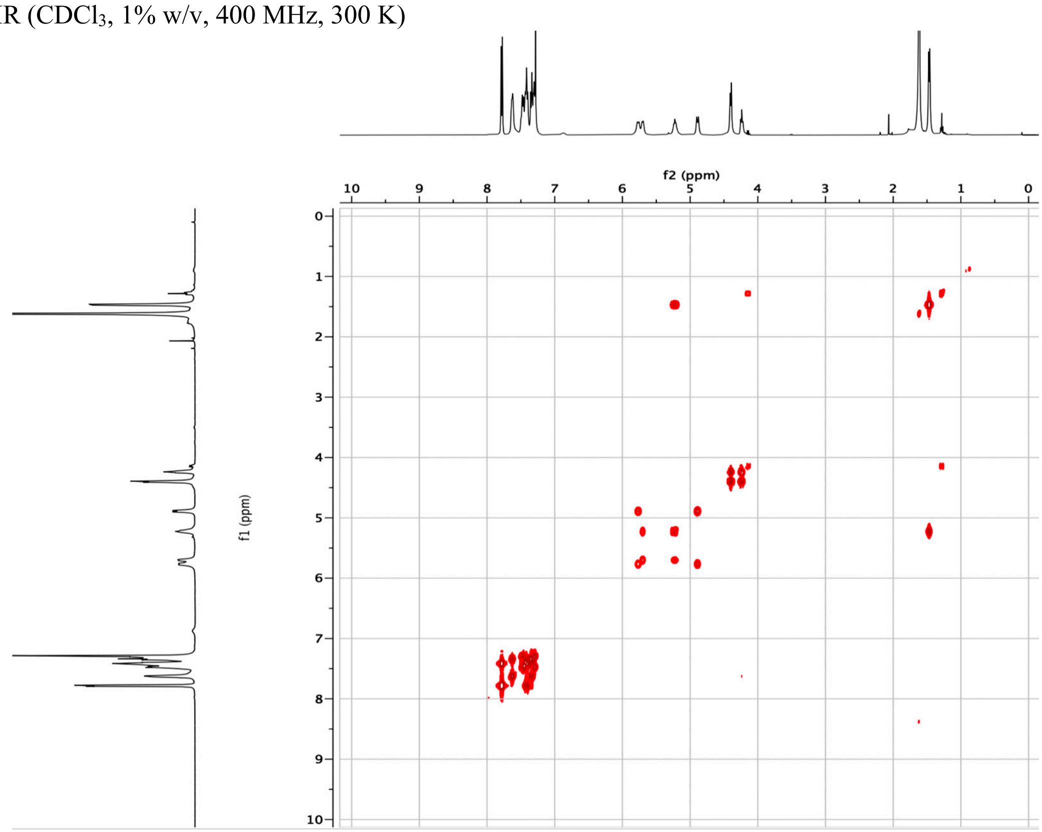

NOESY-NMR ($CDCl_3$, 1% w/v, 400 MHz, 300 K, t_{mix} = 300 ms)

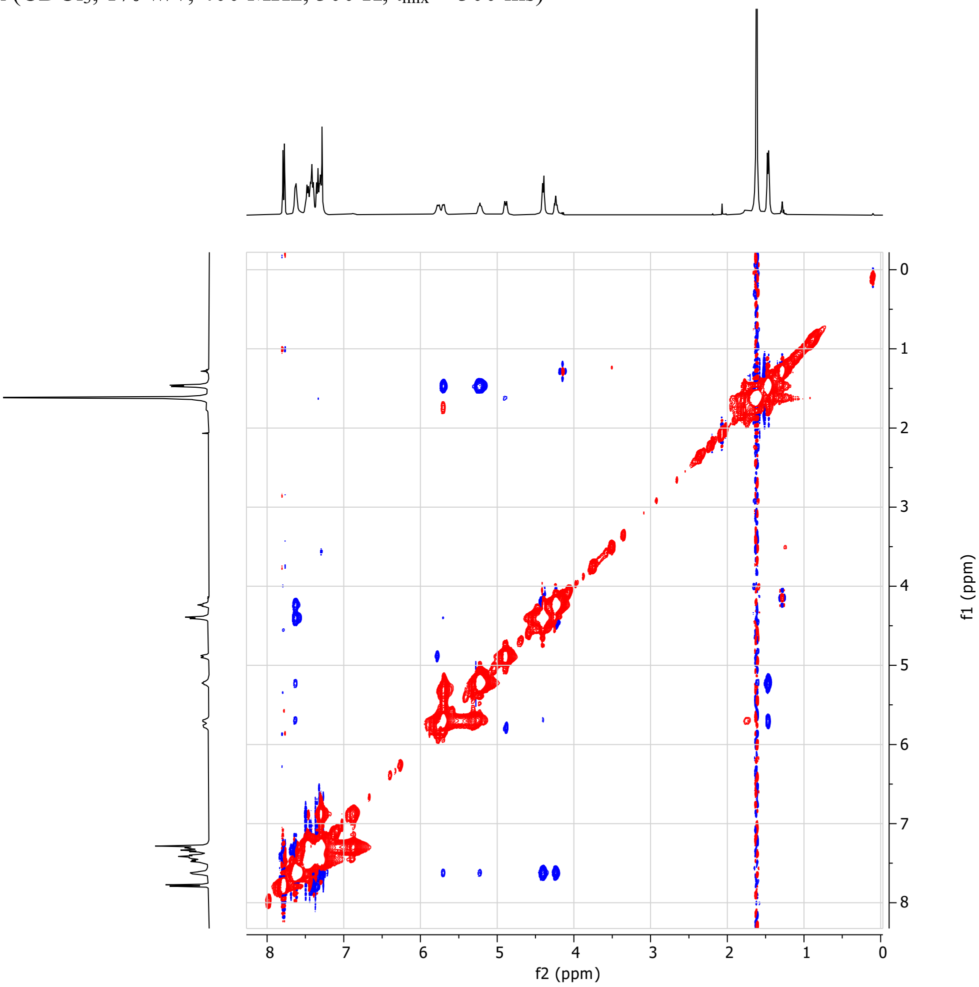

^{1}H NMR (C_6D_6, 1% w/v, 400 MHz, 300 K)

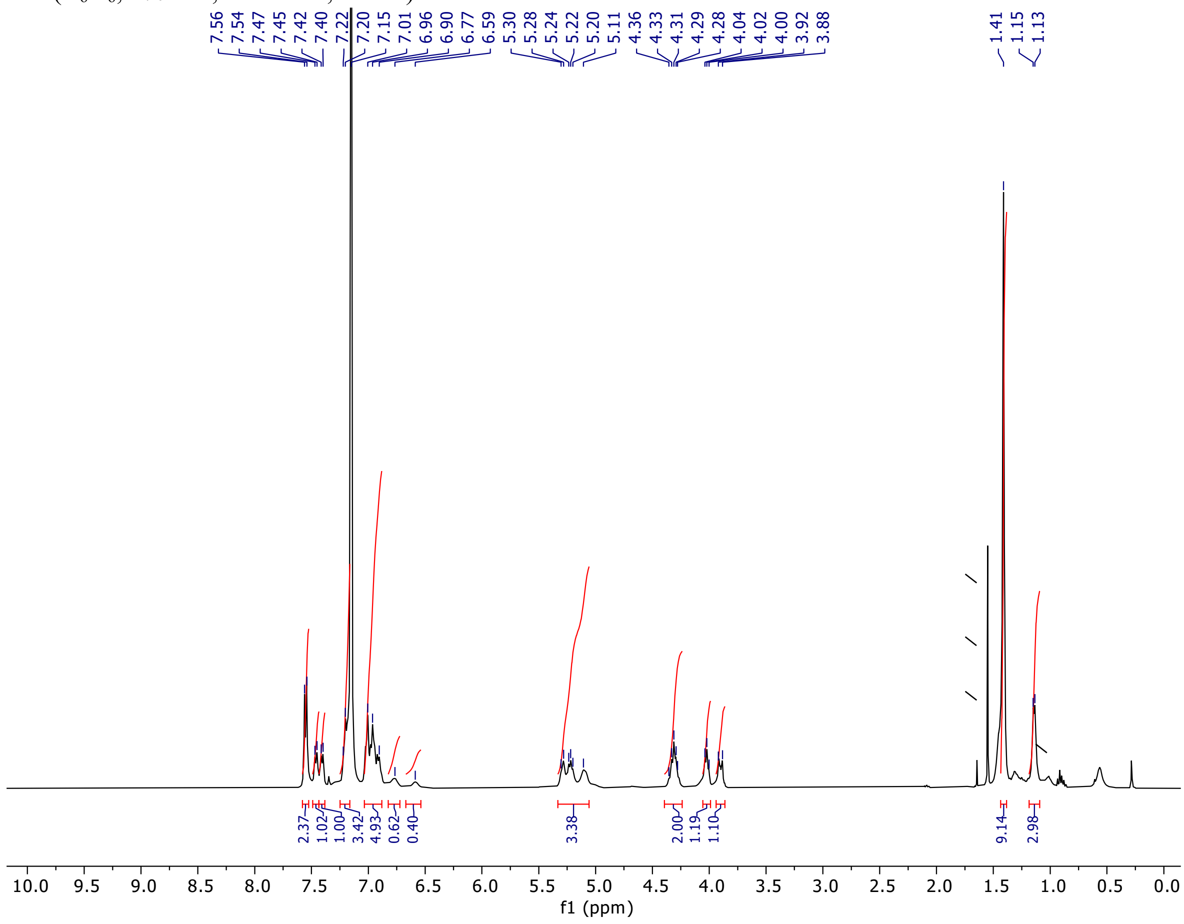

COSY-NMR (C_6D_6, 1% w/v, 400 MHz, 300 K)

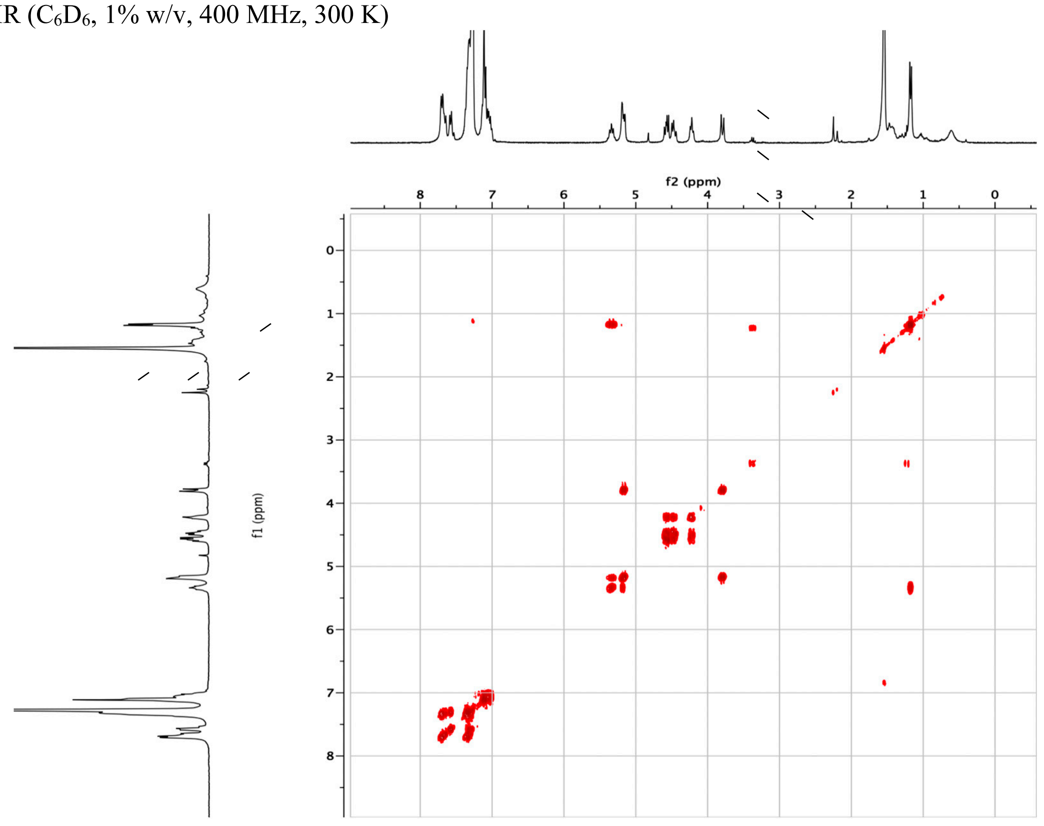

NOESY-NMR (C_6D_6, 1% w/v, 400 MHz, 300 K, t_{mix} = 300 ms)

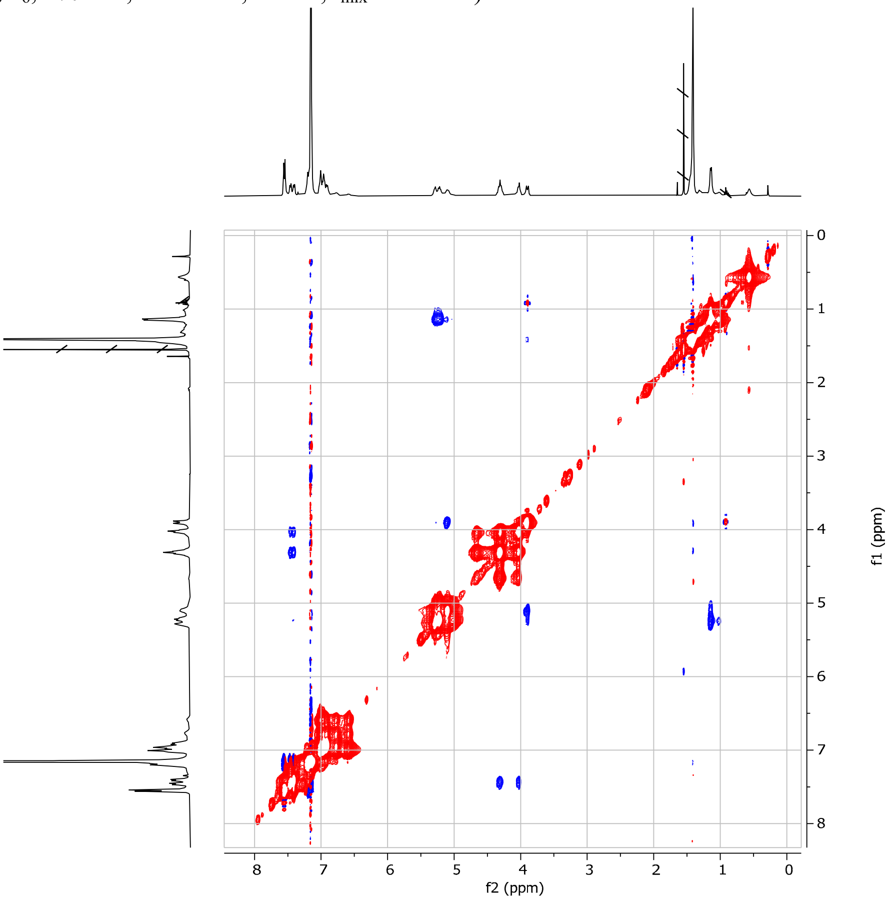

NOESY-NMR (C_6D_6, 1% w/v, 400 MHz, 300 K, t_{mix} = 800 ms)

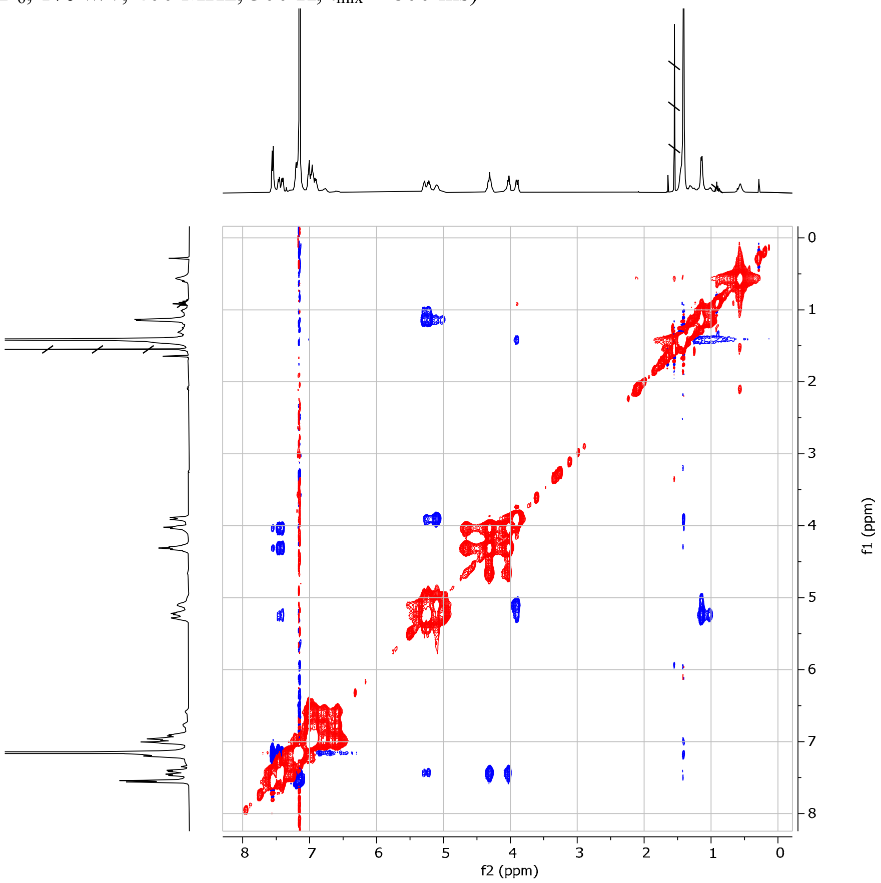

^{13}C NMR (C_6D_6, 1% w/v, 100.7 MHz, 300 K)

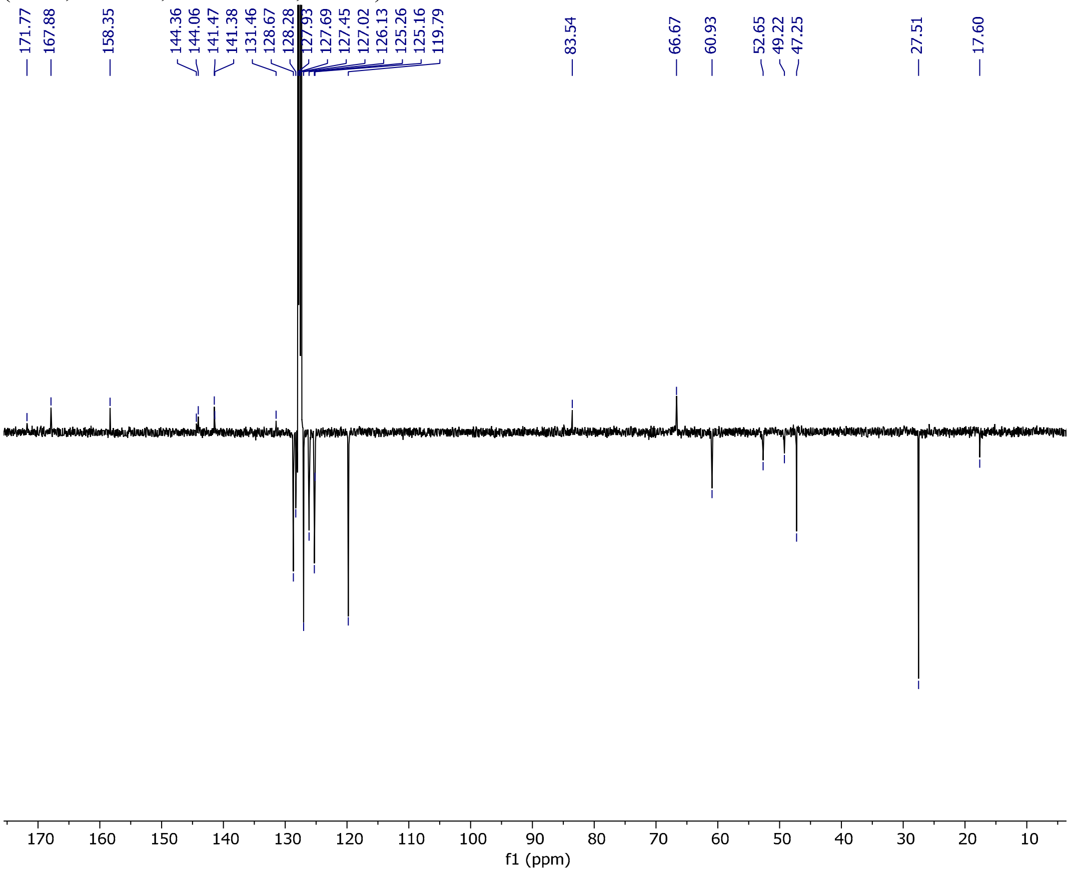

HSQC-NMR (C_6D_6, 1% w/v, 400 MHz, 100.7 MHz, 300 K)

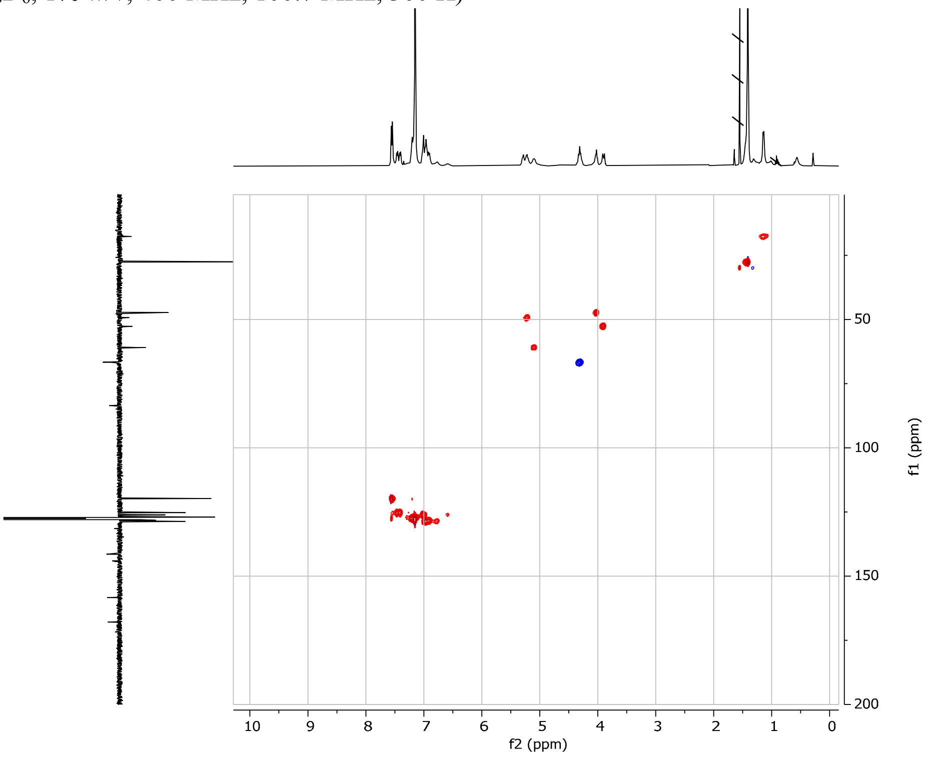

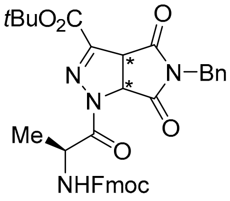

5c

^{1}H NMR ($CDCl_3$, 300 MHz, 300 K)

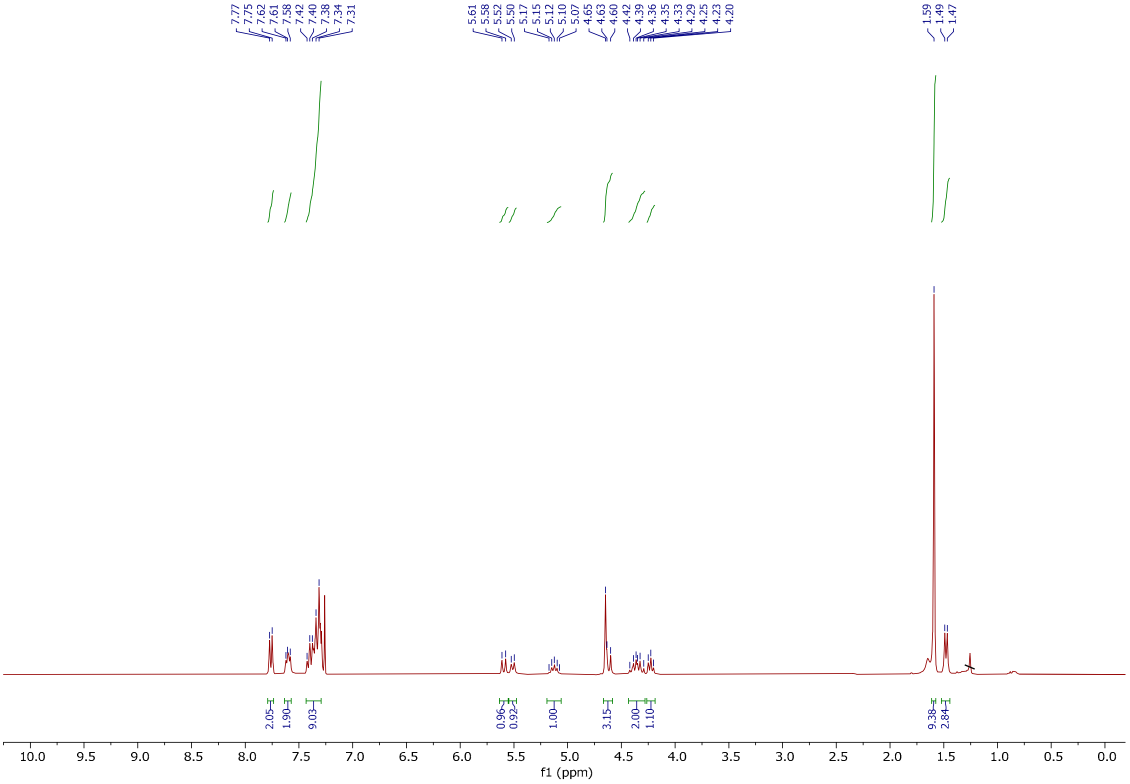

^{13}C NMR ($CDCl_3$, 75 MHz, 300 K)

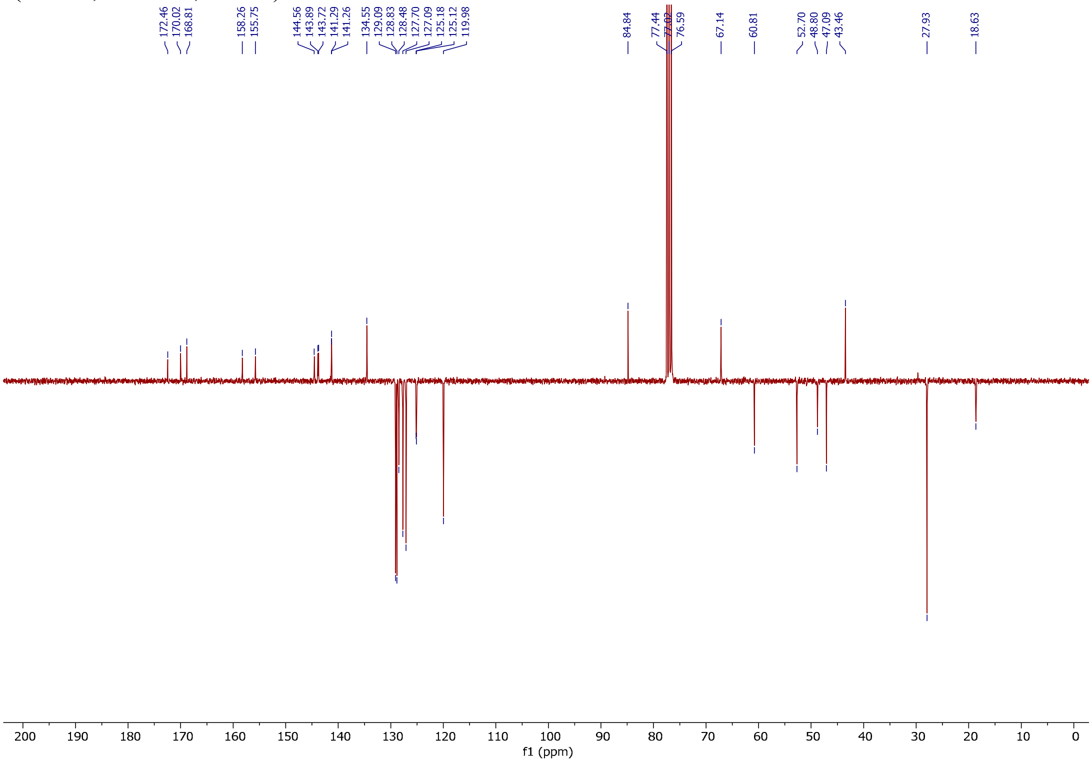

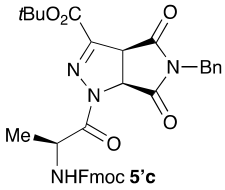

5'c

^{1}H-NMR (CDCl$_3$, 400 MHz, 300 K)

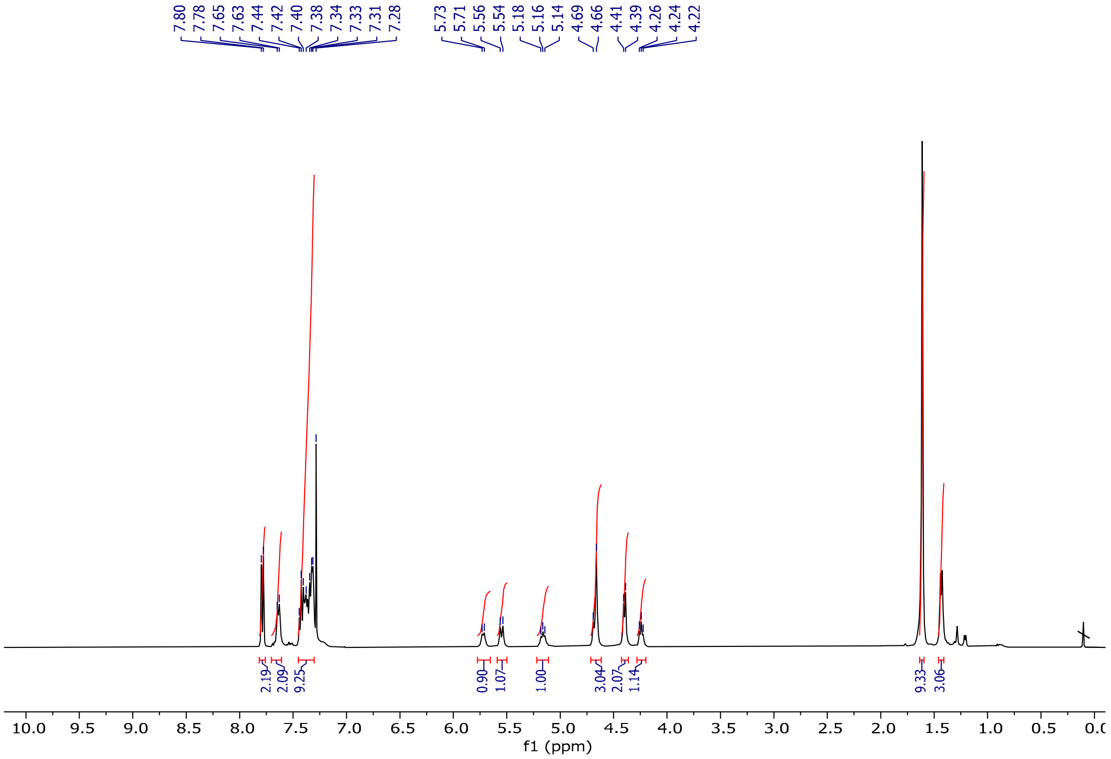

^{13}C-NMR (CDCl$_3$, 100.7 MHz, 300 K)

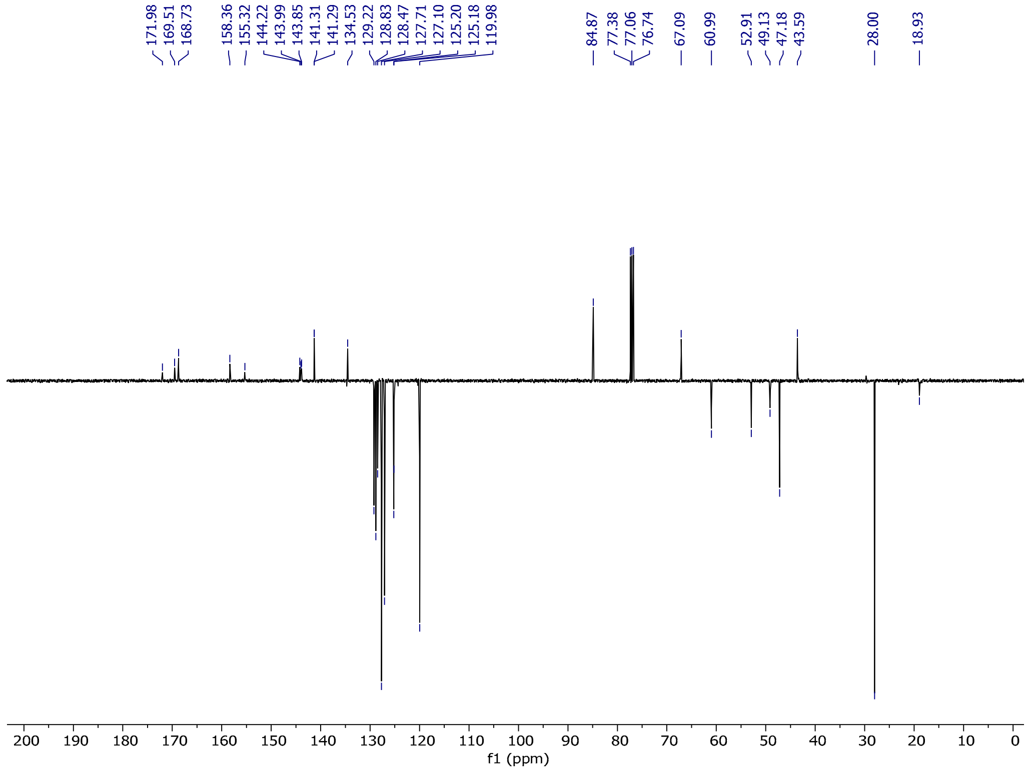

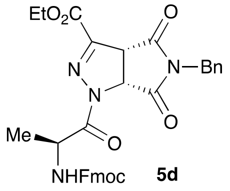

5d

^{1}H NMR ($CDCl_3$, 300 MHz, 300 K)

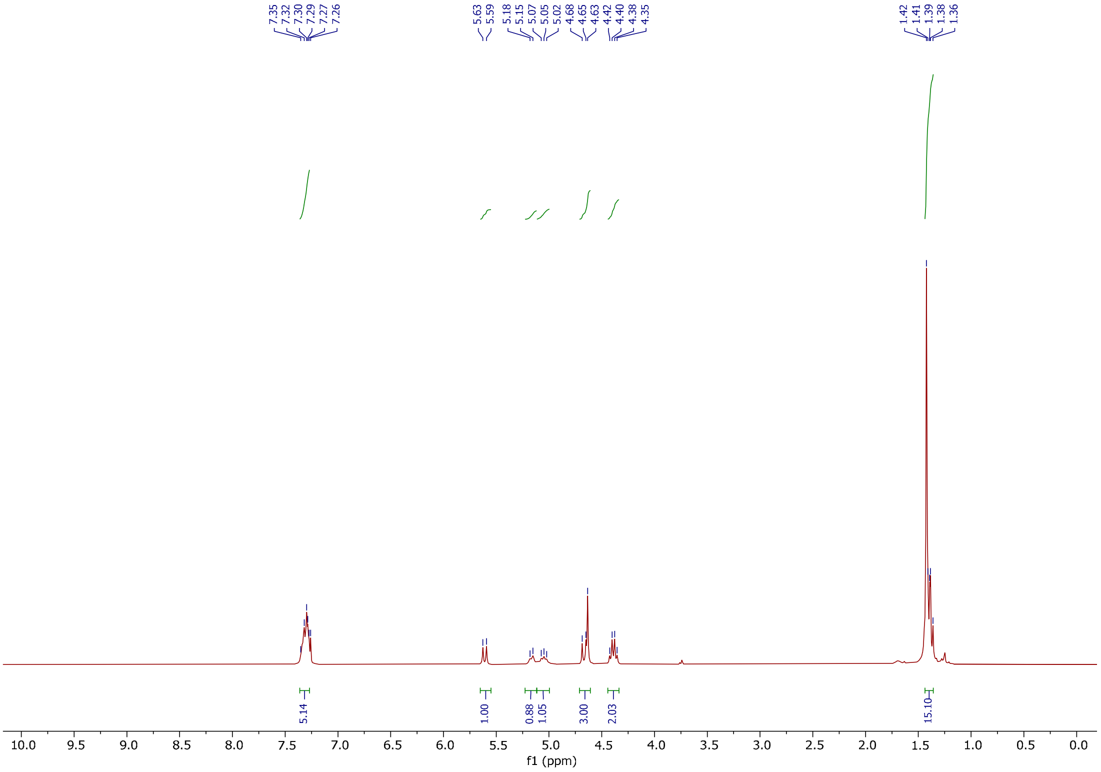

^{13}C NMR ($CDCl_3$, 75 MHz, 300 K)

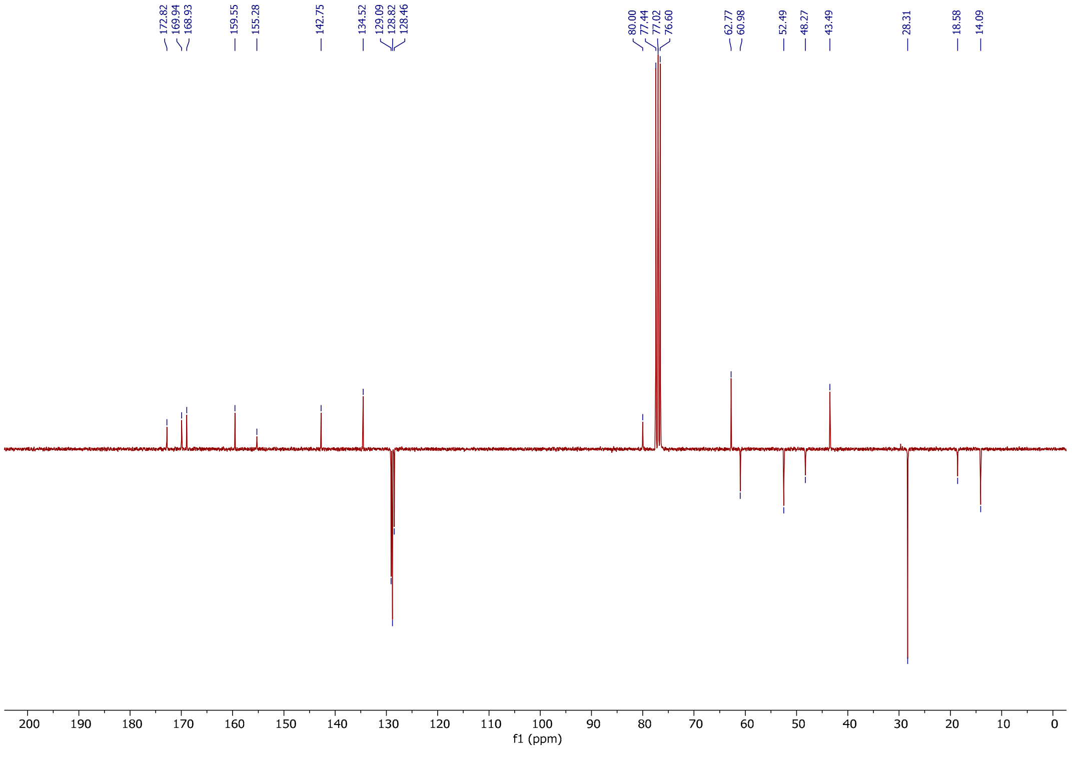

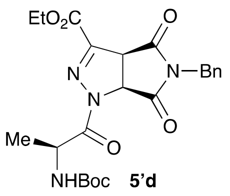

5’d

^{1}H NMR ($CDCl_3$, 400 MHz, 300 K)

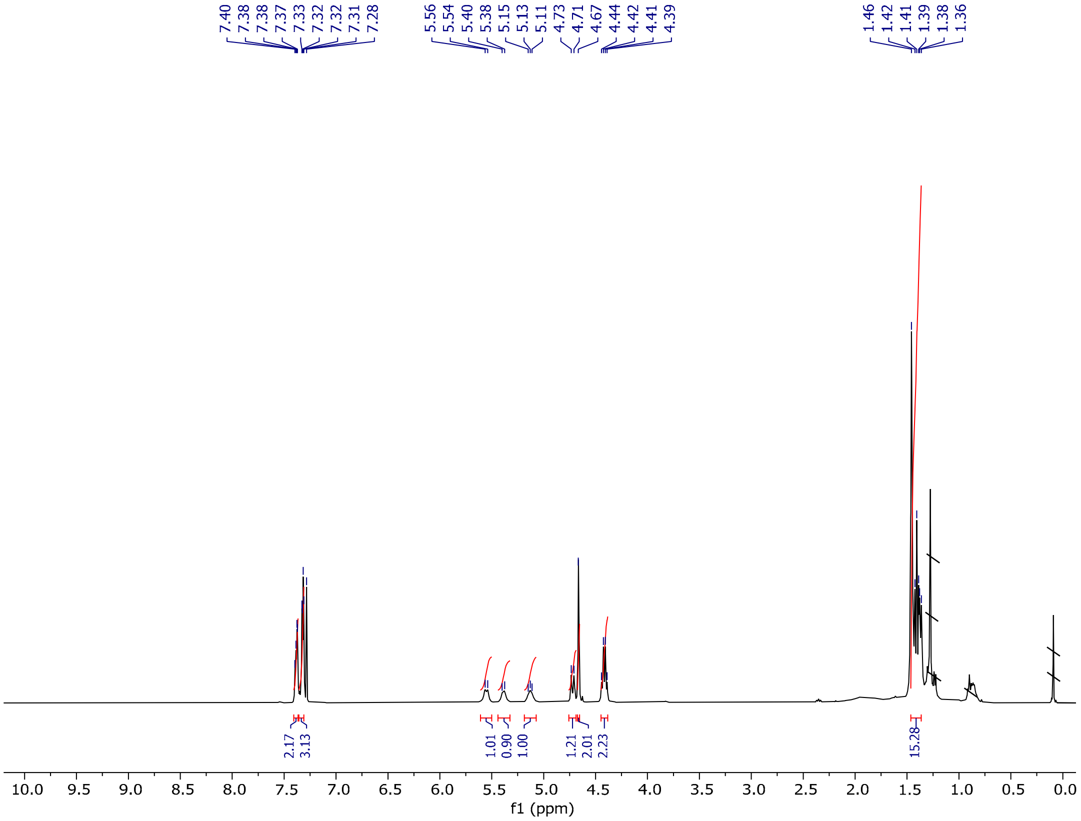

^{13}C NMR ($CDCl_3$, 100.7 MHz, 300 K)

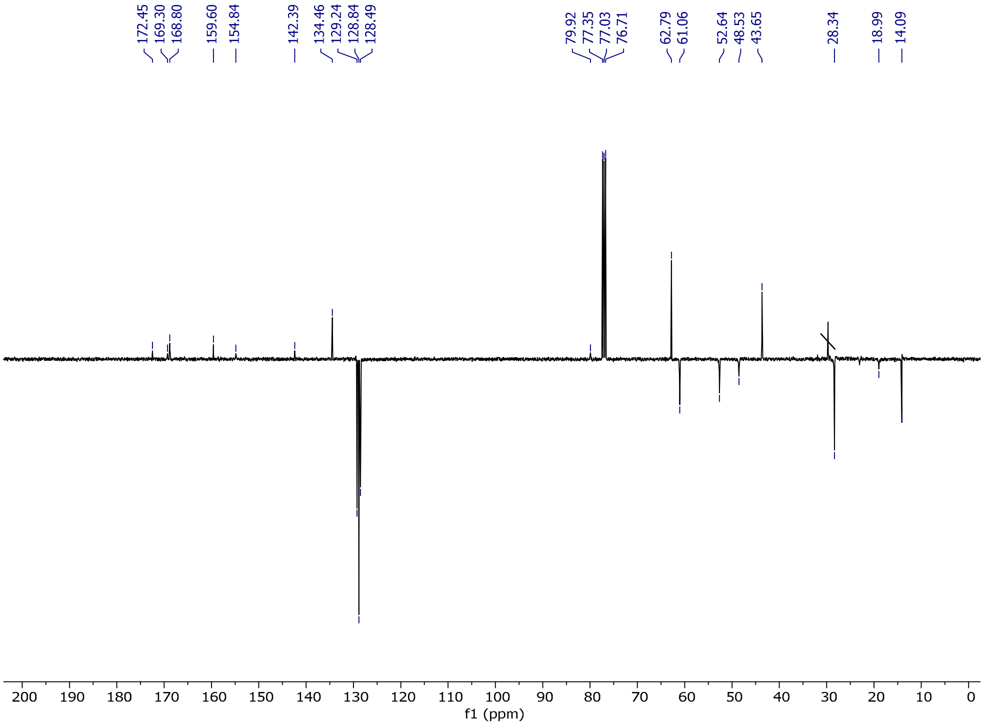

Supplement: Supplementary file 1 [file gels-10-00263-s001.zip › gels-2888454-supplementary.pdf]
